# Development of a *Prevotella bivia* PNA probe and a multiplex approach to detect three relevant species in bacterial vaginosis-associated biofilms

**DOI:** 10.1038/s41522-023-00411-6

**Published:** 2023-06-23

**Authors:** Lúcia G. V. Sousa, Carina Almeida, Christina A. Muzny, Nuno Cerca

**Affiliations:** 1 Centre of Biological Engineering (CEB), Laboratory of Research in Biofilms Rosário Oliveira (LIBRO), University of Minho, Braga, Portugal; 2 LABBELS – Associate Laboratory, Braga, Portugal; 3 INIAV, IP- National Institute for Agrarian and Veterinary Research, Vila do Conde, Portugal; 4 LEPABE - Laboratory for Process Engineering, Environment, Biotechnology and Energy, Faculty of Engineering, University of Porto, Rua Dr. Roberto Frias, Porto, Portugal; 5 Associate Laboratory in Chemical Engineering (ALiCE), Faculty of Engineering, University of Porto, Porto, Portugal; 6 Division of Infectious Diseases, University of Alabama at Birmingham, Birmingham, AL USA

**Keywords:** Biofilms, Infectious-disease diagnostics

## Abstract

Bacterial vaginosis (BV) is the most common vaginal infection worldwide. We developed a peptide nucleic acid (PNA) probe targeting *Prevotella bivia*, a common BV-associated bacteria, and optimized a multiplex approach for detection of *Gardnerella* spp., *P. bivia* and *Fannyhessea vaginae*. Our *P. bivia* PNA probe specifically detected the target species, and the optimized multiplex approach was able to detect the presence of the three species in multi-species BV biofilms.

## Introduction

The most common vaginal dysbiosis among women of reproductive age is bacterial vaginosis (BV)^1^. BV is characterized by a decrease in *Lactobacillus* species and an increase in facultative and strictly anaerobic bacteria which form a polymicrobial biofilm on the vaginal mucosa^2^. *Gardnerella* is the most common BV-associated bacteria (BVAB).This microorganism, as a facultative anaerobe, may displace vaginal lactobacilli on the vaginal mucosa, adhere to vaginal epithelial cells, and initiate BV biofilm formation^3,4^. Due to its virulence factors, *Gardnerella* may have a key role in the development of incident BV (iBV). *Prevotella bivia* is another common BVAB found in high concentrations in women with BV^5^, and has a known symbiotic relationship with *G. vaginalis*^6^. *Fannyhessea vaginae* (previously *Atopobium vaginae*) is also frequently seen in women with BV^7^. It is highly specific, rarely occurs in the absence of *G. vaginalis*, and is found less frequently in healthy women^8^.

A prior study found that the mean relative abundance of *P*. *bivia* and *G. vaginalis* became significantly higher in women with iBV 4 days and 3 days before the day of iBV, respectively. The mean relative abundance of *F. vaginae* became significantly higher on the day of iBV^9^. Thus, *G. vaginalis*, *P. bivia*, and *F. vaginae* may be key BVAB in the pathogenesis of iBV.

Peptide nucleic acid (PNA) probes are polymeric neutral charged probes which bind to nucleic acids without repulsion. PNA-fluorescence in situ hybridization (PNA-FISH) consists of the hybridization of a fluorescent-labeled PNA probe with a complementary sequence of DNA and/or RNA for the detection and identification of microorganisms^10^. We designed a PNA probe for the detection of *P. bivia* and optimized a multiplex approach combining this probe with two previously developed PNA probes specific for *Gardnerella* spp.^11^ and *F. vaginae*^12^, as a new method for detection of these three common BVAB.

A total of sixteen 16 S and ten 23 S *P. bivia* sequences were retrieved from the Arb-Silva database (Version 138.1) and used for alignment in the probe development. The selected 23 S rRNA sequences showed better results than the 16 S rRNA sequences, with several conserved regions with potential to be used in the probe for *P. bivia*. Final probe selection was based on a greater number of perfect matches with *P. bivia* and a lower number of matches with non-target bacterial species. Theoretical sensitivity and specificity were determined by testing a total of 157,859 sequences from the 23 S Arb-Silva REF collection, using the TestProbe tool, from which only ten sequences corresponded to *P. bivia* strains. The theoretical sensitivity was 100% and specificity was 99.9%, since the probe also targeted one out of four *P. denticola* sequences present in the database. The Gibbs free energy and the melting temperature were −17.92 kcal/mol and 56.9 °C, respectively. This *P. bivia* probe was subsequently named PbivPNA1454: Alexa Fluor 488-OO-ATTAAACGTCCATGTCC (Supplementary Fig. 1). Optimizations in the temperature and incubation time were performed (Supplementary Table 1), and the best signal-to-noise ratio was obtained at 58 °C for 90 min.

**Fig. 1:**
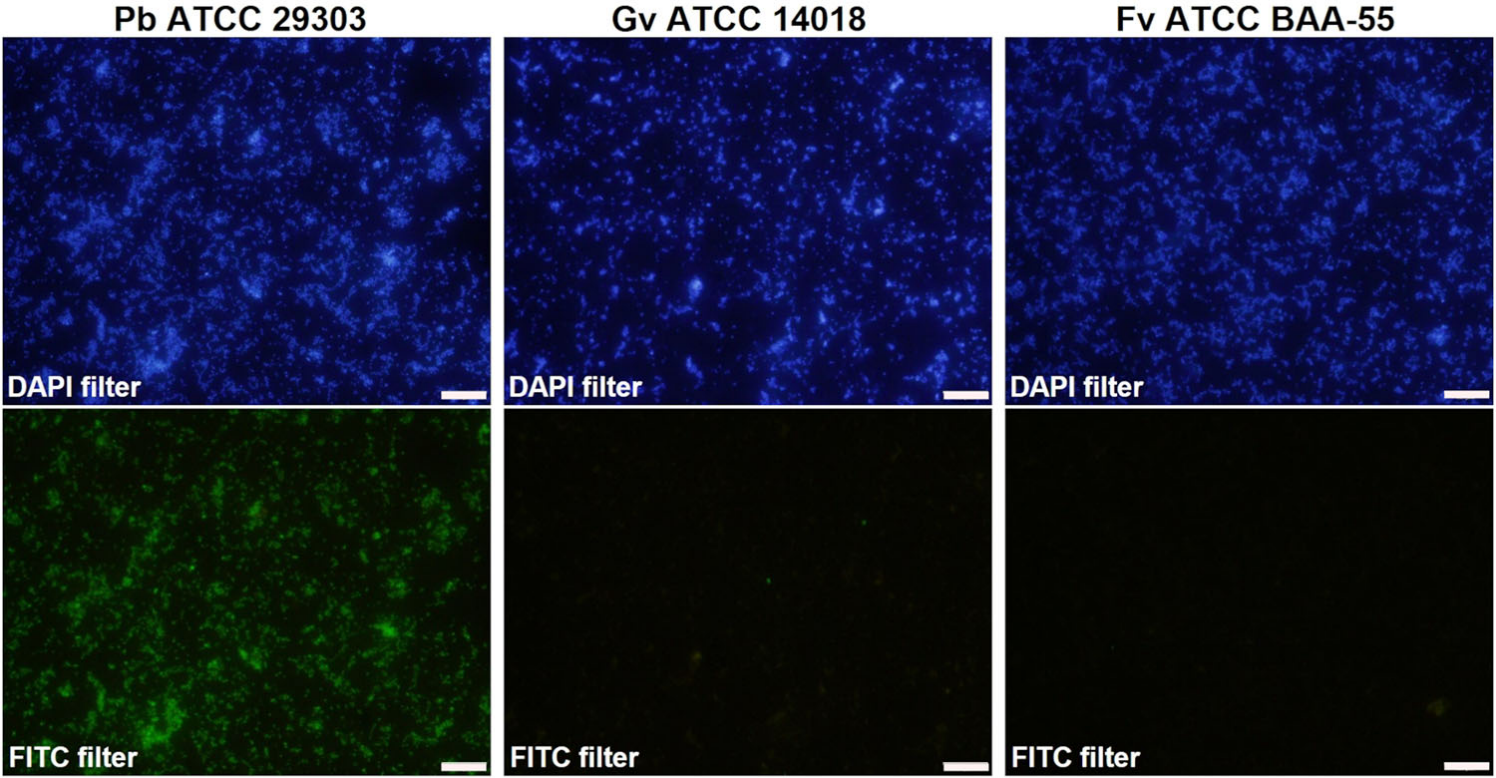
Fluorescence microscopy results of *P. bivia* PbivPNA1454 probe hybridization. The images show examples of DAPI staining (DAPI filter) and *P. bivia* probe hybridization (FITC filter) with the strain *P. bivia* ATCC 29303 (good hybridization), *G. vaginalis* ATCC 14018 (absence of hybridization), and *F. vaginae* ATCC BAA-55 (absence of hybridization). The images were acquired with a magnification of ×400; Scale bars represent 20 μm.

**Fig. 2:**
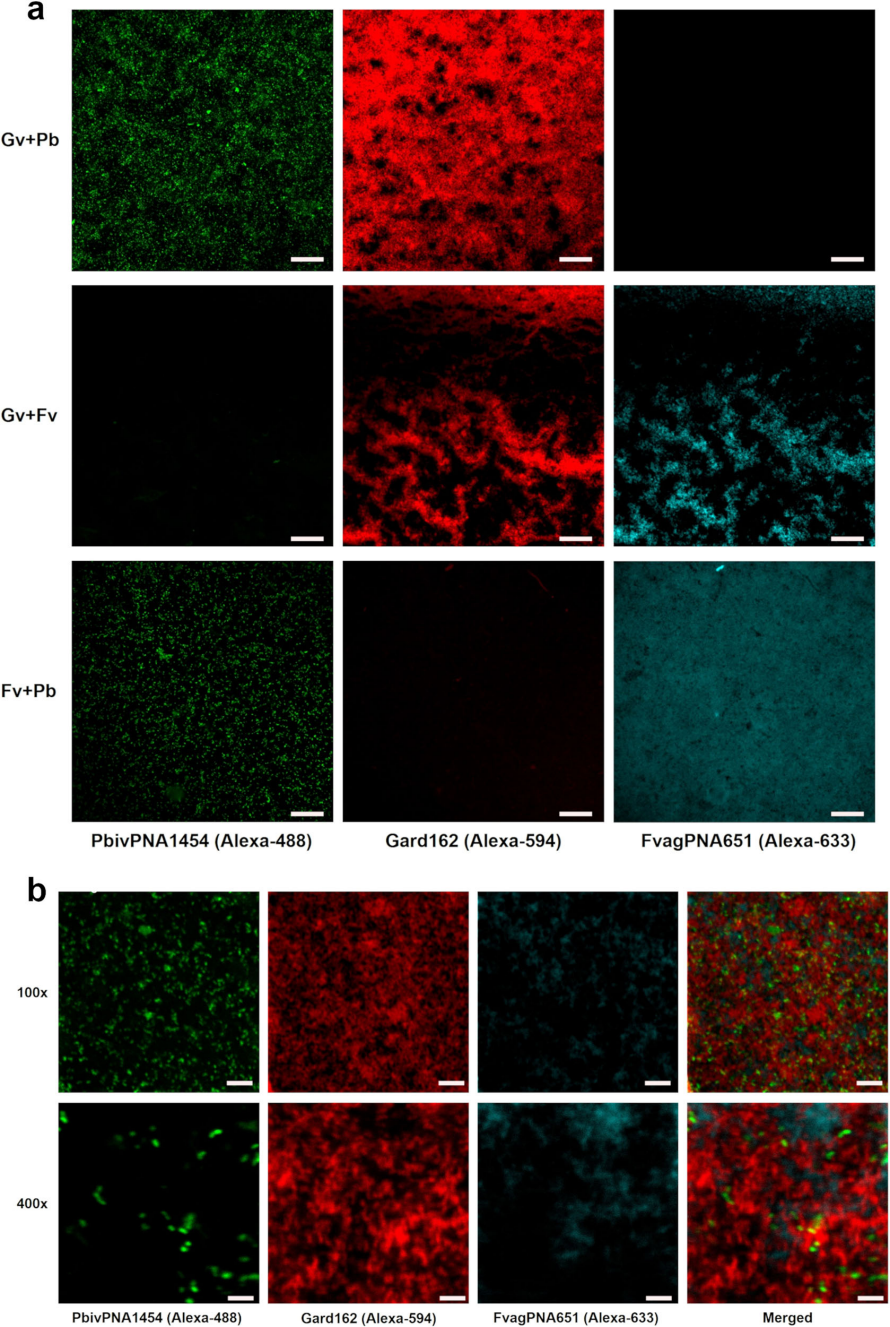
Confocal laser scanning microscopy images of dual and triple-species biofilms analyzed by PNA-FISH. The images, on (**a**), show the hybridization of the PNA probes PbivPNA1454 (green), Gard162 (red), and FvagPNA651 (cyan) in dual-species biofilms of *G. vaginalis* (Gv), *P. bivia* (Pb) and *F. vaginae* (Fv). The images were acquired with a magnification of ×100. **b** Shows images of a triple-species biofilm, acquired using two different magnifications (×100 and ×400). Scale bars represent 10 μm.

The results of hybridization were qualitatively classified based on a previously used four-level scale^11^, including the absence of hybridization (−), poor (+), moderate (++), and good (+++) hybridization (Supplementary Fig. 3). Despite showing distinct levels of hybridization, all the *P. bivia* strains hybridized with the *P. bivia* PNA probe (Supplementary Table 2), which indicates an experimental sensitivity of 100%, 95% confidence interval (CI) [79.4%, 100.0%]. None of the other related species showed a signal of hybridization with the probe (Supplementary Table 3 and Supplementary Fig. 4) indicating an experimental specificity of 100%, 95% CI [92.6%, 100.0%]. Figure 1 shows examples of microscopic images from the hybridization of the *P. bivia* probe with *G. vaginalis*, *P. bivia*, and *F. vaginae*. We then developed a PNA multiplex protocol for the combined detection of these three key BVAB. Keeping in mind that these bacterial species are frequently observed in women with BV, their detection could be an improvement for BV molecular diagnosis and research. For these experiments, dual and triple-species biofilms of *G. vaginalis*, *F. vaginae*, and *P. bivia* were analyzed using PNA probes. Since each probe had different optimal hybridization conditions, a compromise was made to provide the best possible hybridization conditions for all the probes. As such, based on our previous optimization experiments for both the *Gardnerella*^11^ and *F. vaginae*^12^ probes and, considering the optimal conditions for each of the probes, we selected the conditions of 60 °C and 90 min. Before analysis of the triple-species biofilms, the efficiency of the *P. bivia* PNA probe was determined in biofilms at the hybridization conditions used in the multiplex protocol. Cell counts of *P. bivia* were performed using DAPI staining and the PNA probe, as presented in Supplementary Fig. 2, resulting in an efficiency of 91.7%. Figure 2a represents examples of hybridization of the three probes in dual-species biofilms. In all experiments, the probes were able to hybridize with the corresponding species, while not showing significant cross-hybridization with the other species. When analyzing triple-species biofilms at higher magnifications (Fig. 2b), it was also possible to observe the distribution of the three species in the biofilm. The probes were capable of differentiating between the spatial localization of each species. The composition of the triple-species biofilms was determined by cell counts using the specific PNA probes for *G. vaginalis*, *F. vaginae*, and *P. bivia*, which resulted in a biofilm composition (mean ± SD) of 49.7% ( ± 4.2%) of *G. vaginalis*, 45.7% ( ± 3.4%) of *F. vaginae*, and 4.6% ( ± 3.6%) of *P. bivia*. It was possible to distinguish the signal of the 3 species and the proportions found are in line with other studies that show that, in triple-species biofilms formed with the same species, *P. bivia* is usually outnumbered by other two species^13,14^.

PNA-FISH is currently being used in several applications primarily due to the fact that it is a simple and rapid technique to perform, compared to traditional culture methods for infectious diseases diagnosis^10^. However, this technique also presents some disadvantages, and the major one relies on the fact that it requires specialized technicians to perform and interpret microscopy results. Also, PNA-FISH sensitivity, in terms of number/concentration of cells that can be detected in a sample, is usually lower than in other molecular techniques, such as polymerase chain reaction (PCR)^15^, and the analysis of samples requires high/stable concentrations of ribosomal contents, which can compromise the analysis of biological samples^10^. Moreover, the cost of PNA probes remains higher than other methods^10^.

In summary, we have developed a PNA approach for the detection of three key BVAB, including the development of a *P. bivia* PNA probe. This method represents a useful tool for future research studying the composition and structure of BV biofilms. This will contribute to additional knowledge about the etiology of this common vaginal infection.

## Methods

### PNA probe in-silico design

A PNA probe specific for the detection of *P. bivia* was designed following the protocol of previous studies^11,12^. A set of sequences from 16 S and 23 S rRNA collections was selected from the Arb-Silva database (Version 138.1) (https://www.arb-silva.de/search/) with lengths >1200 bp or >1600 bp, respectively, and quality scores >90. The *P. bivia* rRNA sequences available in this database, as well as sequences from closely related bacterial species, were then aligned using the Clustal Omega tool (https://www.ebi.ac.uk/Tools/msa/clustalo/). Conserved regions of the sequences were chosen as potential probes, based on perfect matches with sequences of interest and mismatches with sequences of non-interest. The theoretical sensitivity and specificity of the probes were determined, as previously demonstrated^16^, and the probes were tested using the TestProbe tool from Arb-Silva with no mismatches allowed. The chosen sequences were then classified based on the number of target strains/species, the position of mismatches in closely related strains/species, the percentage of GC content between 40% and 60%, melting temperature >50 °C^17^, Gibbs free energy between −13 kcal/mol and −20 kcal/mol^18^, and theoretical values of sensitivity and specificity. The Gibbs free energy and the melting temperature were calculated, based on previous studies^19–21^. The sequence with the best theoretical results was selected for probe synthesis (Eurogentec, Seraing, Belgium) and the probe was linked to an Alexa Fluor molecule via a double 8-amino-3,6-dioxaoctanoic acid linker.

### Bacterial growth conditions

Several strains of *P. bivia* and other BVAB were used to determine the experimental sensitivity and specificity of the *P. bivia* probe. The bacteria were grown on Columbia Agar Base (Liofilchem, Roseto degli Abruzz, Italy) supplemented with 5% (v/v) of defibrinated horse blood (Oxoid, Basingstoke, UK), excluding *S. sanguinegens* that was grown on chocolate agar supplemented with 10% of inactivated horse serum (Biowest, Nuaillé, France). The plates were incubated at 37 °C and 10% CO_2_ for 48 h. For anaerobic bacteria (i.e., *Actinomyces urogenitalis*, *Aerococcus christensenii*, *Bifidobacterium bifidum*, *Campylobacter ureolyticus*, *F. vaginae*, *L. iners*, *Megasphaera micronuciformis*, *Mobiluncus curtisii*, *M. mulieris*, *Mycoplasma hominis*, *Peptostreptococcus anaerobius*, *Porphyromonas asaccharolytica*, *Prevotella* spp., *Propionibacterium acnes*, *S. sanguinegens* and *Veillonella parvula*), the plates were maintained under anaerobic conditions using anaerobic gas-generating packs (AnaeroGen Atmosphere Generation system, Oxoid). *Acinetobacter baumannii* was grown at 30 °C for 24 h.

### PNA-FISH procedure

For the PNA-FISH procedure, a bacterial suspension of each strain was prepared in 1× phosphate buffered saline (PBS) solution and with the optical density (OD) adjusted to 0.1, followed by a twofold dilution. Twenty µL of the suspension was then spread on epoxy-coated microscope glass slides with ten wells (Thermo Fisher Scientific, Lenexa, KS) and left to dry at 37 °C. After this time, the fixation and permeabilization step was performed using 10 µL of 100% (v/v) methanol (Thermo Fisher Scientific) for 15 min, followed by 20 µL of 4% (w/v) paraformaldehyde (Thermo Fisher Scientific) for 10 min, and 10 µL of 50% (v/v) ethanol (Thermo Fisher Scientific) for 15 min. Afterward, the slides were left to dry at room temperature. The hybridization step was then performed by adding 10 μL of hybridization solution^12^ containing 200 nM of PNA probe to each well of the slides and covering the slides with a coverslip. The slides were then incubated in moist and opaque containers at different temperatures and times. Time and temperature optimizations for hybridization were performed using the strain *P. bivia* ATCC 29303. Temperatures between 53 °C and 63 °C and times of 60–90 min were evaluated. The optimized time and temperature were used for sensitivity and specificity determination experiments. After this time, the slides were removed and immersed in washing solution and incubated again for an additional 30 min at the same temperature. After washing, the slides were allowed to air dry and protected from light until microscopic analysis.

### Microscopic analysis

Microscopic analysis was performed using an Olympus BX51 epifluorescence microscope (Olympus, Lisbon, Portugal) equipped with the FITC filter (BP 470-490, FT500, LP 516 sensitive to the Alexa Fluor 488 molecule). The fluorescence signal of the probe was observed using the filter FITC and the other filters were used to discard any autofluorescence signal from the cells. A negative control was included in the experiments with no probe added to the hybridization solution. The images were acquired with a 40× objective (Numerical aperture: 0.75) and using the same time of exposure for target and non-target species.

### Experimental sensitivity and specificity determination

For determination of the experimental sensitivity and specificity of the *P. bivia* PNA probe, sixteen different strains of *P. bivia* and forty-eight other species associated either with BV or vaginal microbiome were used, respectively. The procedure of PNA-FISH hybridization was performed and the values of sensitivity and specificity were calculated, as described previously^16^. The experiments were repeated with, at least two, independent assays for each species/strain evaluated.

### Determination of *P. bivia* PNA probe efficiency in biofilm

Single-species biofilms of *P. bivia* were performed on 24-wells plates (Orange Scientific, Braine L’Alleud, Belgium) for 24 h. Briefly an inoculum of *P. bivia* ATCC 29303 was prepared in New York City III (NYCIII) broth medium^22^ supplemented with 10% (v/v) of inactivated horse serum and incubated in anaerobic conditions for 24 h. After that, the inoculum concentration was adjusted to 10^7^ CFU/mL,^14^ and 1 mL dispensed on each well. The plate was incubated at 37 °C in anerobic conditions. After 24 h, the medium was removed and the biofilms were washed once with PBS. The biofilms were then resuspended on 1 mL of PBS and four serial dilutions were performed. Twenty microliters of each dilution were spread on epoxy microscope glass slides and set to dry. The fixation and hybridization procedures were performed as described above, using a temperature of 60 °C and 90 min for hybridization with the PbivPNA1454 probe. Before microscopic analysis, the cells were stained with DAPI at 2.5 μg/µL. For each dilution, 20 fields were randomly visualized, and the cells were counted per image with the DAPI staining and the PNA probe. The experiments were performed with three independent assays.

### Multiplex approach for quantification of species in triple-species biofilms

To optimize the PNA-FISH multiplex approach for BV research, we used our *P. bivia* probe with two previously developed PNA probes: Gard162^11^ with an Alexa Fluor 594, and FvagPNA651^12^ with an Alexa Fluor 633 (Eurogentec).

Triple-species biofilms were grown in 24-well plates. For each experiment, an inoculum of each species of *G. vaginalis* ATCC 14018, *F. vaginae* ATCC BAA-55, and *P. bivia* ATCC 29303 was prepared in NYCIII supplemented with 10% (v/v) of inactivated horse serum and incubated at 37 °C and anaerobic conditions for 24 h. After 24 h, the bacterial concentration of the inoculums was determined by reading the optical density (OD) at 620 nm and adjusted to 10^7^ CFU/mL^14^ by a dilution in NYCIII broth. For each species, 111 µL of the adjusted suspension was combined on each plate well for a final volume of 1 mL. The biofilms were incubated for 24 h, at 37 °C in anaerobic conditions. After that time, the biofilms were washed once with PBS and then resuspended on 1 mL of PBS. Therefore, serial dilutions of triple-species biofilms were performed and 20 µL of each dilution was spread on epoxy glass slides and set to dry. The fixation and the hybridization steps were performed, using the 3 probes in the hybridization step, with the hybridization conditions of 60 °C and 90 min.

Using the fluorescence microscope, 20 images of each dilution were randomly taken using the filters appropriated for the observation of each probe. The cells from each species were counted and the composition of the triple-species biofilms was determined using the efficiency of each PNA probe. The experiments were performed with three independent assays.

### Multiplex approach for discrimination of biofilm species by CLSM

Dual and triple-species biofilms of *G. vaginalis*, *F. vaginae* and *P. bivia* were performed on 8-well chamber slides (Thermo Fisher Scientific™ Nunc™ Lab-Tek™). Each 24 h-inoculum, adjusted to 10^7^ CFU/mL, was then dispensed in the corresponding wells to perform the dual- and triple-species biofilm experiments, for a final volume of 400 μL in NYCIII. The chamber slides were then incubated at 37 °C, in anaerobic conditions for 24 h. After 24 h, the biofilms were washed once using 0.9% (w/v) NaCl and set to dry at room temperature. After the fixation step, as described above, 30 µL of hybridization solution containing the 3 PNA probes, at a concentration of 200 nM, were dispensed on each well and covered with a coverslip. The PNA-FISH hybridization was conducted at 60 °C for 90 min followed by the washing step for 30 min. The biofilms were visualized using an Olympus™ Fluo-View FV1000 (Olympus) confocal laser scanning microscope (CLSM). The experiments were repeated twice.

### Statistical analysis

Confidence intervals for experimental sensitivity and specificity were calculated based on the Clopper-Pearson method using the MedCalc software (Version 22.005, available at: https://www.medcalc.org/calc/diagnostic_test.php).

### Reporting summary

Further information on research design is available in the Nature Research Reporting Summary linked to this article.

## Supplementary information

Supplementary Information

Reporting Summary

## Data Availability

All data generated or analyzed during this study are included in this published paper and in the supplementary files.
