# Supplementary material for: Development of a Prevotella bivia PNA probe and a multiplex approach to detect three relevant species in bacterial vaginosis-associated biofilms
Source: NPJ Biofilms Microbiomes. 2023 Jun 23;9:42. doi: 10.1038/s41522-023-00411-6 (PMC10290050; doi:10.1038/s41522-023-00411-6)

Development of a *Prevotella bivia* PNA probe and a multiplex approach to detect three relevant species in bacterial vaginosis-associated biofilms

Lúcia G. V. Sousa[1,2], Carina Almeida[3,4,5], Christina A. Muzny[6], Nuno Cerca[1,2*]

[1]Centre of Biological Engineering (CEB), Laboratory of Research in Biofilms Rosário Oliveira (LIBRO), University of Minho, Braga, Portugal.

[2]LABBELS – Associate Laboratory, Braga, Portugal.

[3]INIAV, IP- National Institute for Agrarian and Veterinary Research, Vila do Conde, Portugal.

[4]LEPABE - Laboratory for Process Engineering, Environment, Biotechnology and Energy, Faculty of Engineering, University of Porto, Rua Dr. Roberto Frias, Porto, Portugal.

[5]Associate Laboratory in Chemical Engineering (ALiCE), Faculty of Engineering, University of Porto, Porto, Portugal.

[6]Division of Infectious Diseases, University of Alabama at Birmingham, Birmingham, AL, United States.

Supplementary Table 1: Optimization results of the hybridization of PbivPNA1454 probe with the strain *P. bivia* ATCC 29303 for the different temperatures and times tested

Temperature (ºC)	Time (min)	Hybridization results
**53**	60	++
	90	++
**56**	60	++
	90	+
**58**	60	++
	90	+++
**60**	60	++
	90	++
**63**	60	++
	90	+

Supplementary Table 2: Determination of experimental sensitivity of PbivPNA1454 probe. Results of hybridization of *P. bivia* PNA probe with different strains of *P. bivia*

Strain	Reference	Hybridization results
Prevotella bivia	ATCC 29303	+++
Prevotella bivia	CCUG 33360	++
Prevotella bivia	CCUG 33961	+++
Prevotella bivia	CCUG 33962	+++
Prevotella bivia	CCUG 34043	+++
Prevotella bivia	CCUG 34044	++
Prevotella bivia	CCUG 34045	++
Prevotella bivia	CCUG 34046	++
Prevotella bivia	CCUG 34047	+++
Prevotella bivia	CCUG 35221	++
Prevotella bivia	CCUG 35880	+++
Prevotella bivia	CCUG 36740	++
Prevotella bivia	CCUG 44195	+++
Prevotella bivia	CCUG 48913	+++
Prevotella bivia	CCUG 56865	+++
Prevotella bivia	CCUG 59496	+++

Hybridization results were evaluated qualitatively according to the classification: (-) Absence of hybridization; (+) Poor hybridization; (++) Moderate hybridization; (+++) Good hybridization.

Supplementary Table 3: Determination of experimental specificity of PbivPNA1454 probe. Results of hybridization of *P. bivia* PNA probe with distinct bacterial species

Species	Reference	Hybridization results
Acinetobacter baumannii	CCUG 59798	-
Actinomyces neuii	UM067	-
Actinomyces urogenitalis	CCUG 44038	-
Aerococcus christensenii	CCUG 28826	-[1]
Bacillus firmus	UM034	-[1]
Bifidobacterium bifidum	CCUG 59492	-
Brevibacterium ravenspurgense	CCUG 42923	-
Campylobacter ureolyticus	CCUG 44295	-
Corynebacterium tuscaniense	UM137	-
Enterococcus faecalis	UM035	-[1]
Escherichia coli	UM056	-
Fannyhessea vaginae	ATCC BAA-55	-[1]
Gardnerella leopoldii	UM034	-[1]
Gardnerella piotii	UM035	-[1]
Gardnerella swidsinskii	UM094	-[1]
Gardnerella vaginalis	ATCC 14018	-[1]
Gemella haemolysans	UM034	-[1]
Lactobacillus crispatus	EX533959VCO6	-
Lactobacillus gasseri	ATCC 9857	-[1]
Lactobacillus iners	ATCC 55195	-
Lactobacillus rhamnosus	CECT 288	-
Lactobacillus vaginalis	UM062	-
Megasphaera micronuciformis	CCUG 45952T	-

Mobiluncus curtisii	ATCC 35241	-
Mobiluncus mulieris	ATCC 35239	-[1]
Mycoplasma hominis	UM054	-[1]
Neisseria gonorrhoeae	CCUG 13281	-[1]
Nosocomiicoccus ampullae	UM121	-
Peptostreptococcus anaerobius	ATCC 27337	-
Porphyromonas asaccharolytica	CCUG 7834T	-
Prevotella buccalis	CCUG 44127	-
Prevotella copri	CCUG 58058T	-
Prevotella denticola	CCUG 29542T	-
Prevotella disiens	CCUG 59491	-
Prevotella intermedia	CCUG 31410	-
Prevotella melaninogenica	CCUG 65141	-[1]
Prevotella nigrescens	CCUG 25289	-[1]
Prevotella timonensis	CCUG 59487	-
Propionibacterium acnes	UM034	-
Shigella spp.	UM137	-
Sneathia sanguinegens	CCUG 66076	-[1]
Staphylococcus epidermidis	UM066	-
Staphylococcus haemolyticus	UM066	-
Staphylococcus hominis	UM224	-
Staphylococcus saprophyticus	UM121	-
Staphylococcus simulans	UM059	-
Streptococcus agalactiae	UM035	-
Veillonella parvula	CCUG 59474	-

Hybridization results were evaluated qualitatively according to the classification: (-) Absence of hybridization; (+) Poor hybridization; (++) Moderate hybridization; (+++) Good hybridization. [1]These species showed some autofluorescence signal detected in the FITC filter.

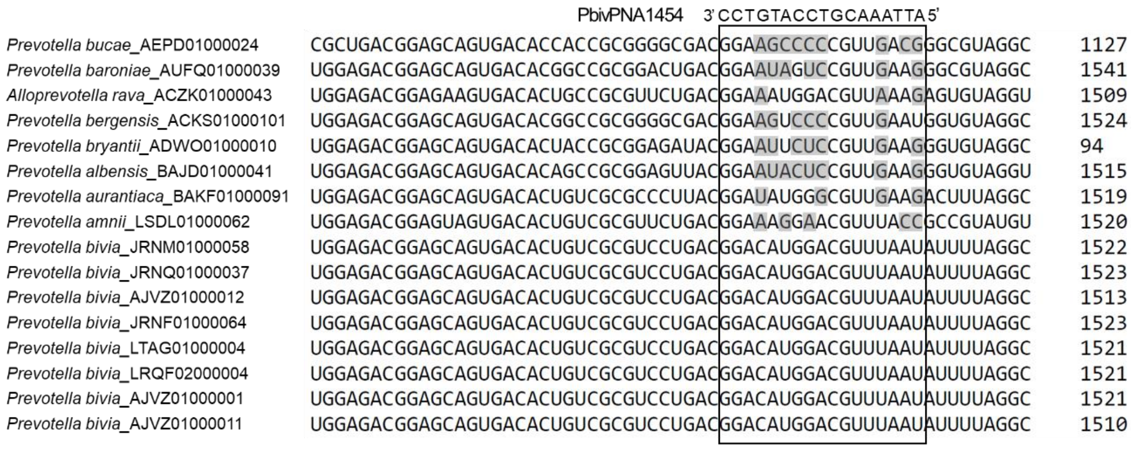

Supplementary Figure 1: Alignment of 23S rRNA sequences from species of interest and non-interest from Arb-Silva database. The indicated region corresponds to the sequence of the *P. bivia* PNA probe and mismatches with the non-interest sequences are highlighted. The complementary sequence of the probe is shown above.

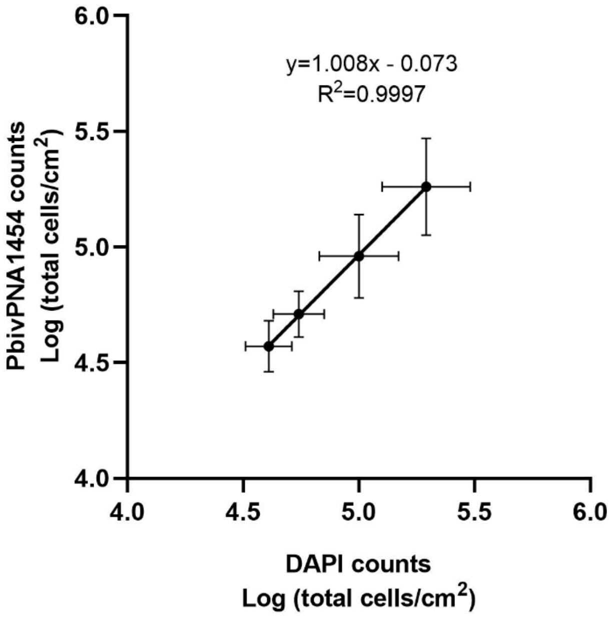

Supplementary Figure 2: Calibration curve of PbivPNA1454 probe efficiency in biofilm, determined by the correlation between cell counts with DAPI staining and the PNA probe. Each point represents average of counts from three independent experiments and error bars represent standard deviation.

Supplementary Figure 3: The following images present the fluorescence microscopy results of *P. bivia* PNA probe hybridization with all the tested species for sensitivity and specificity. The images were acquired using DAPI filter (left image, blue) and FITC filter (right image, green), sensitive to the Alexa fluor 488, with a magnification of 400x; scale bars represent 20 μm.

Prevotella bivia **CCUG 33360**

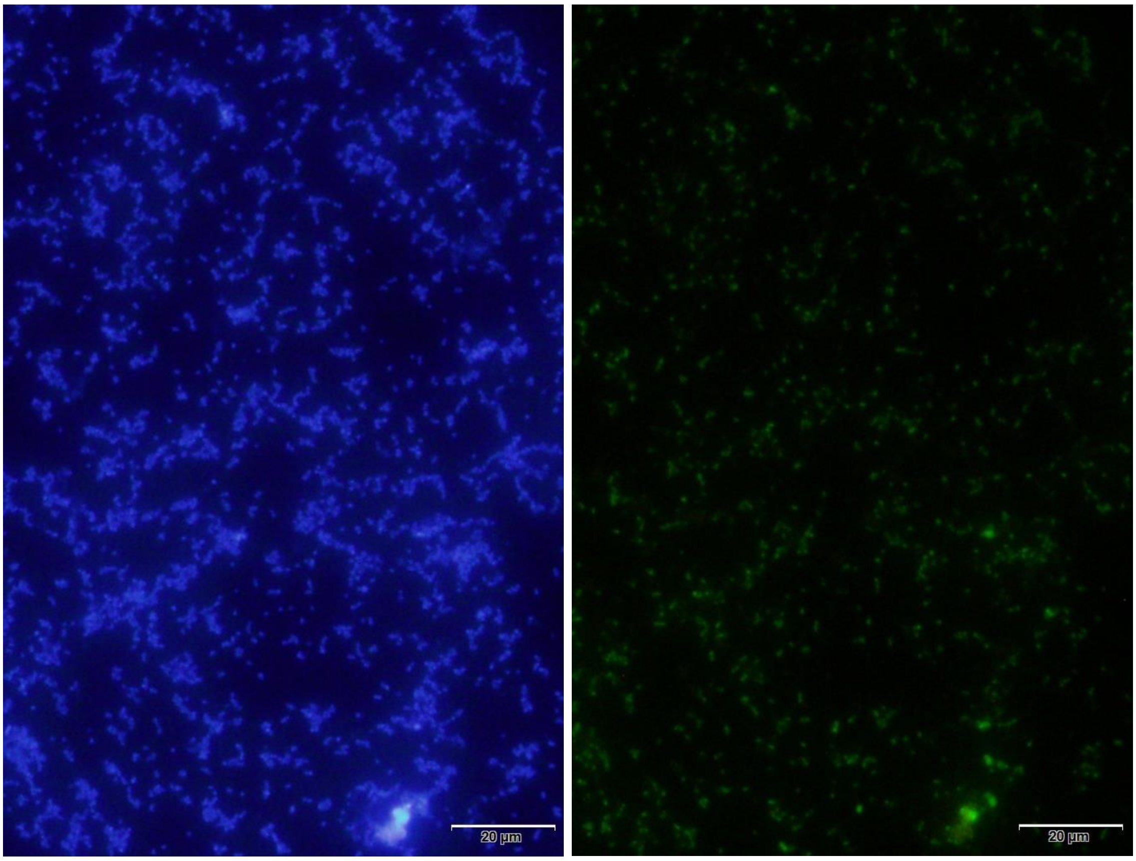

Prevotella bivia CCUG 33961

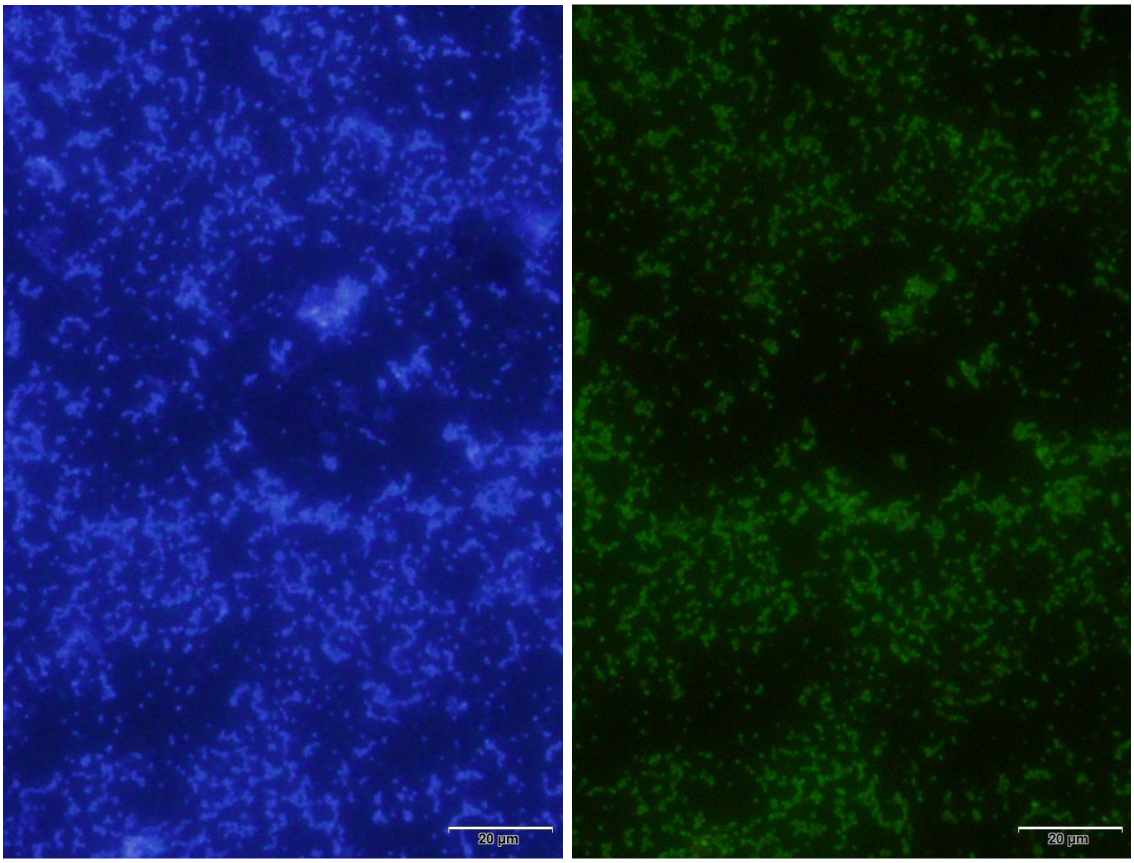

Prevotella bivia CCUG 33962

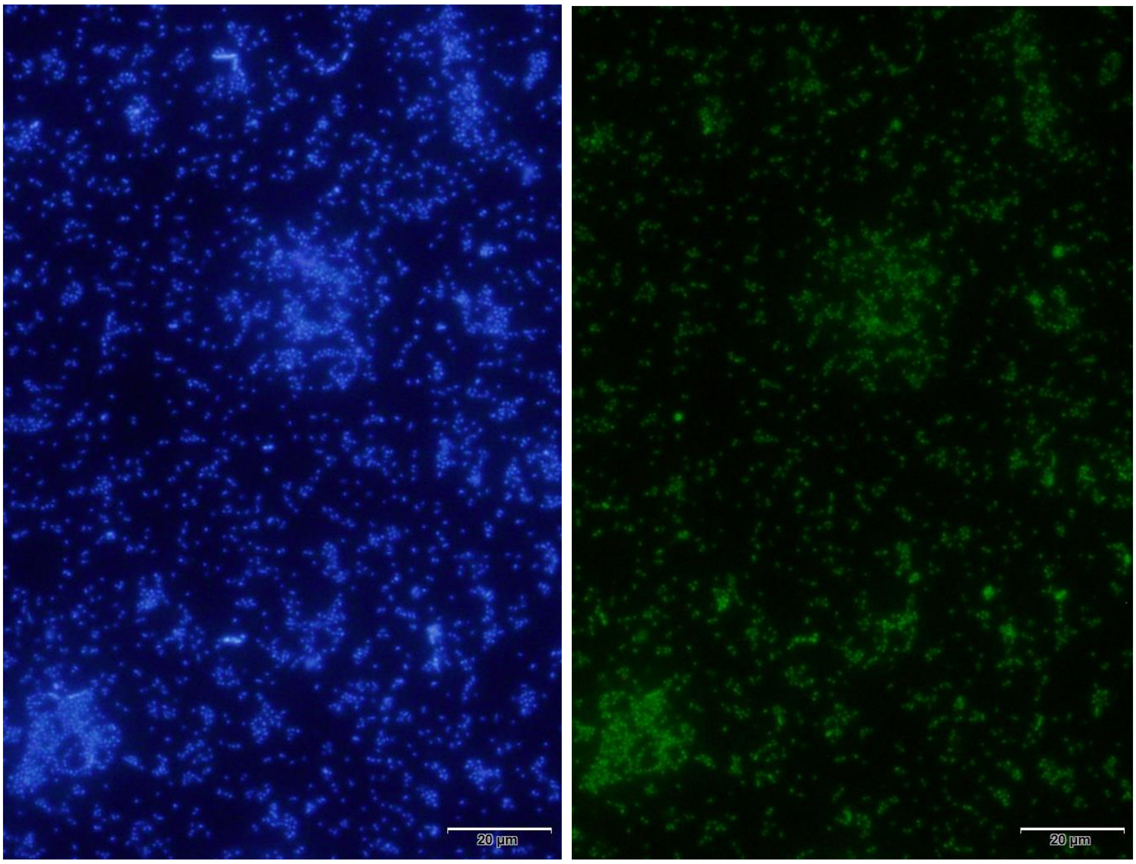

Prevotella bivia CCUG 34043

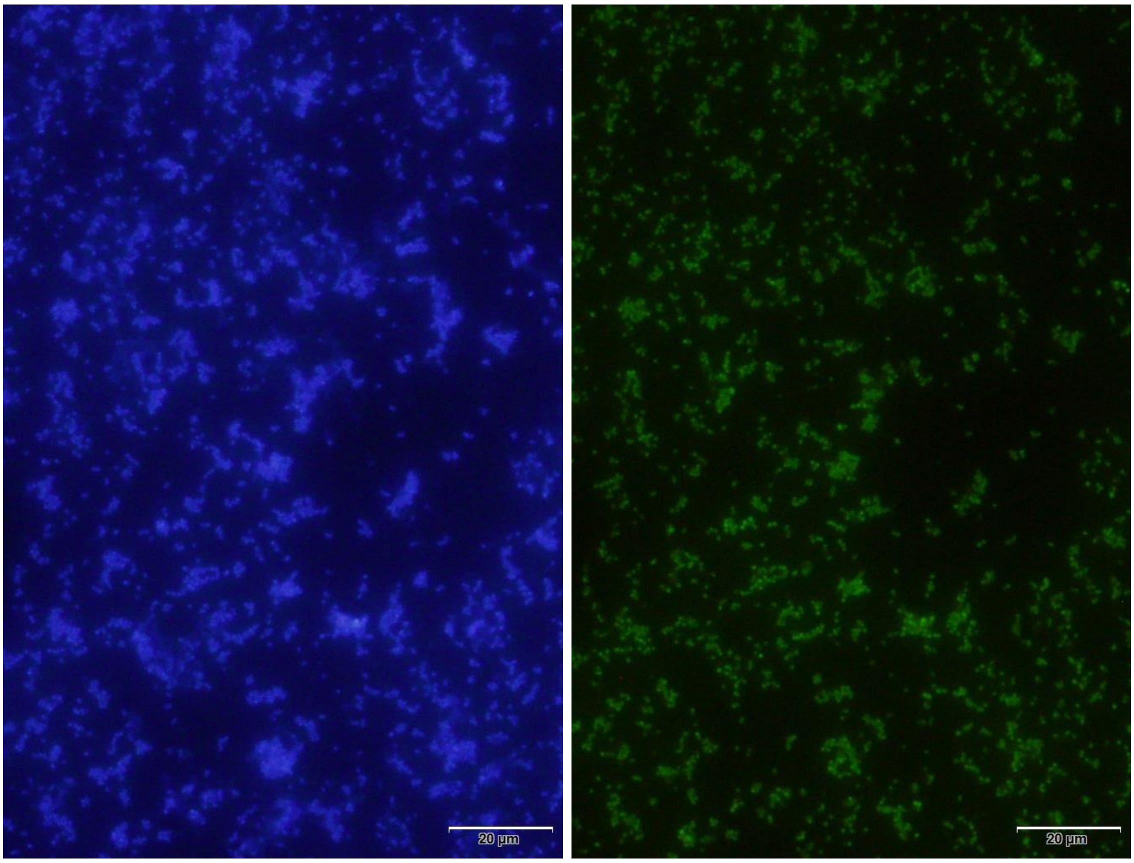

Prevotella bivia CCUG 34044

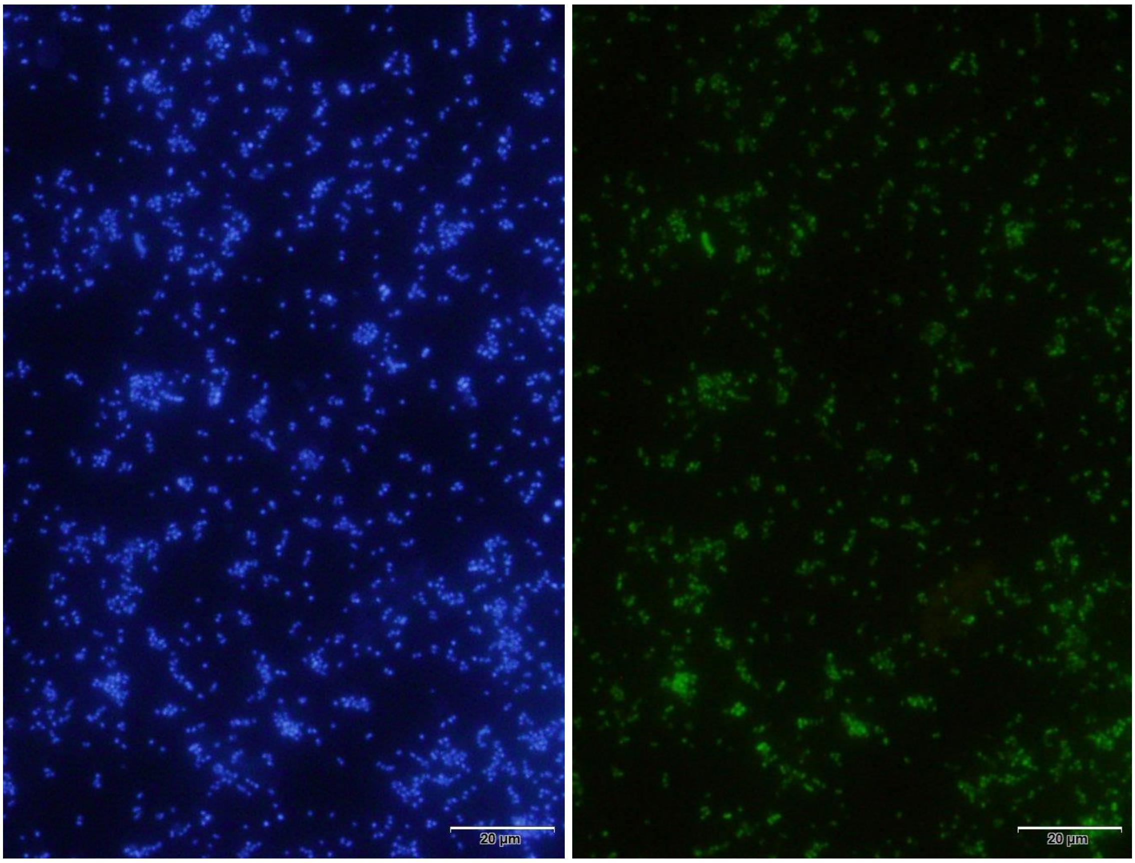

Prevotella bivia **CCUG 34045**

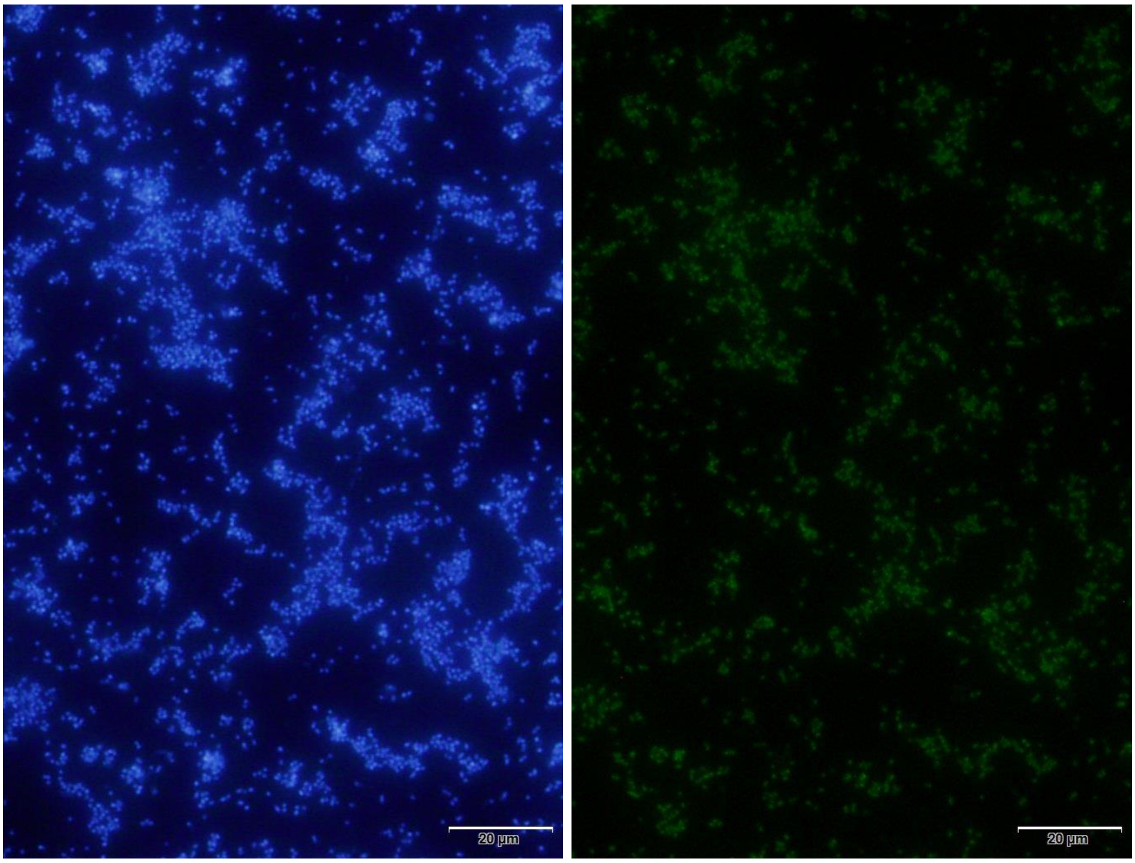

Prevotella bivia **CCUG 34046**

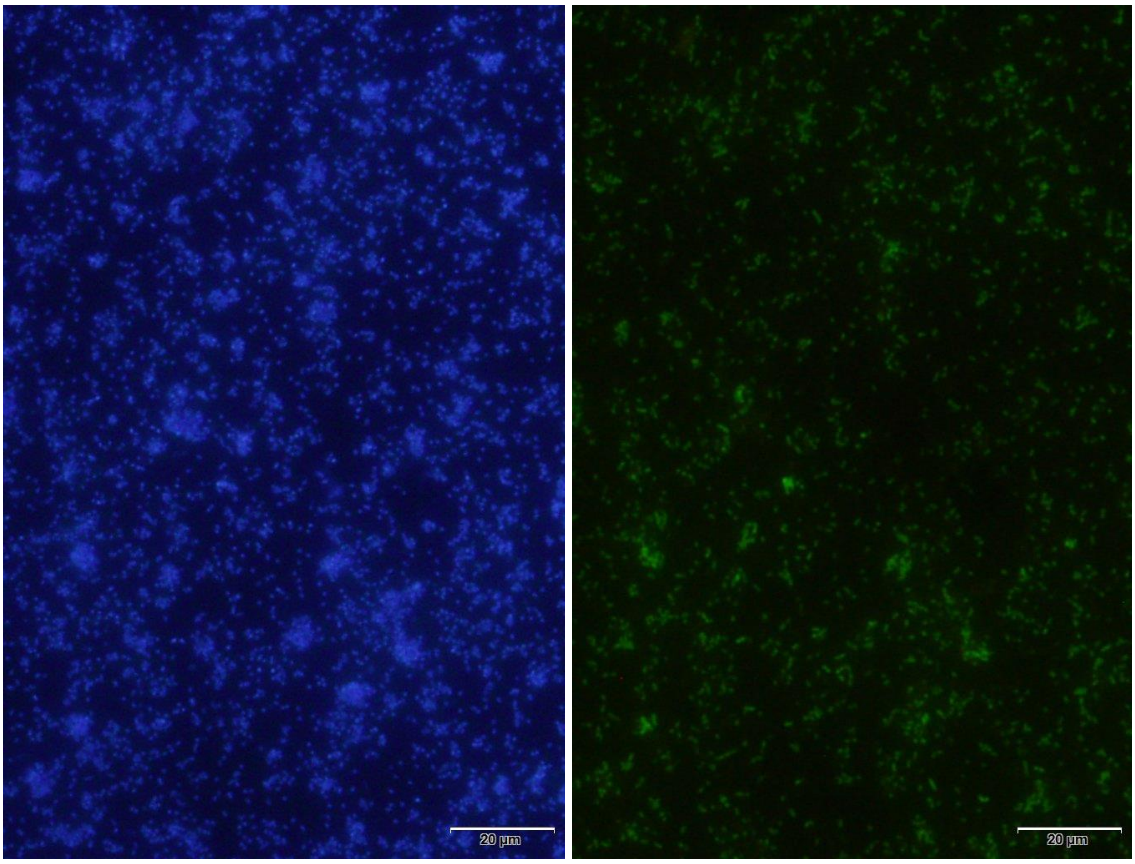

Prevotella bivia CCUG 34047

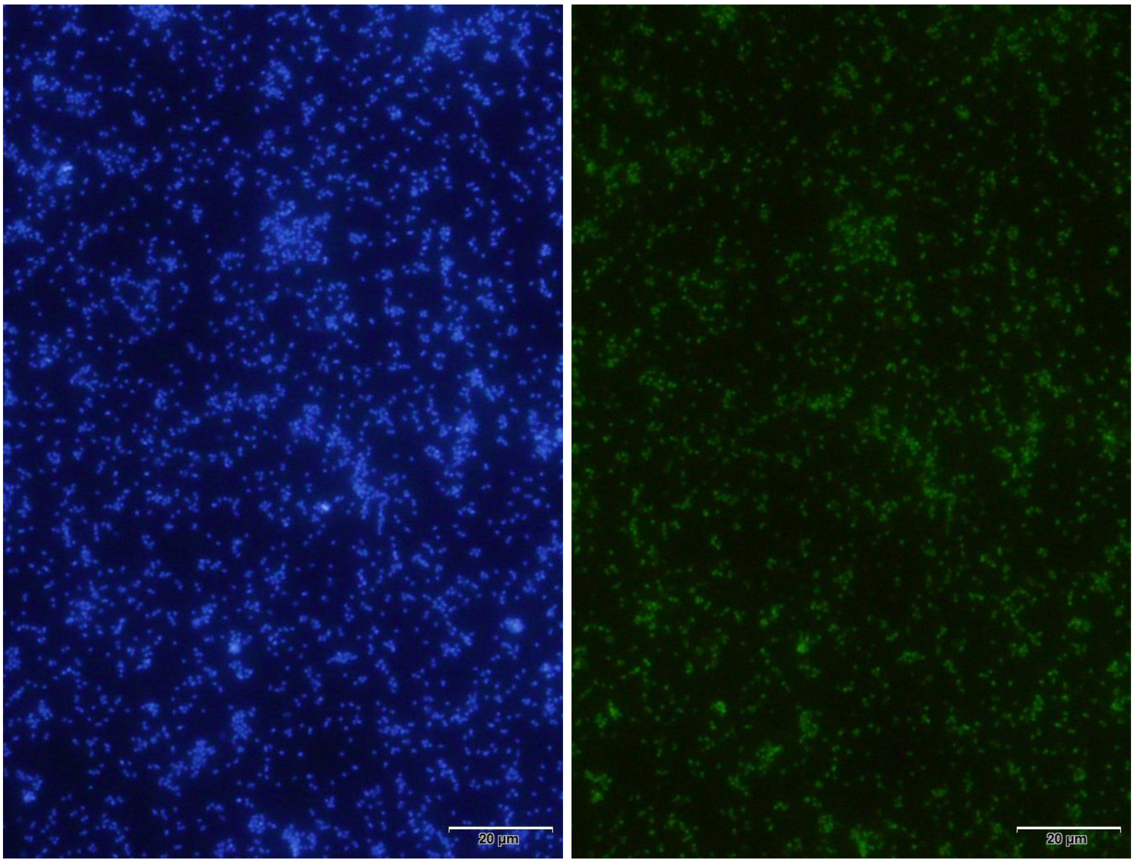

Prevotella bivia CCUG 35221

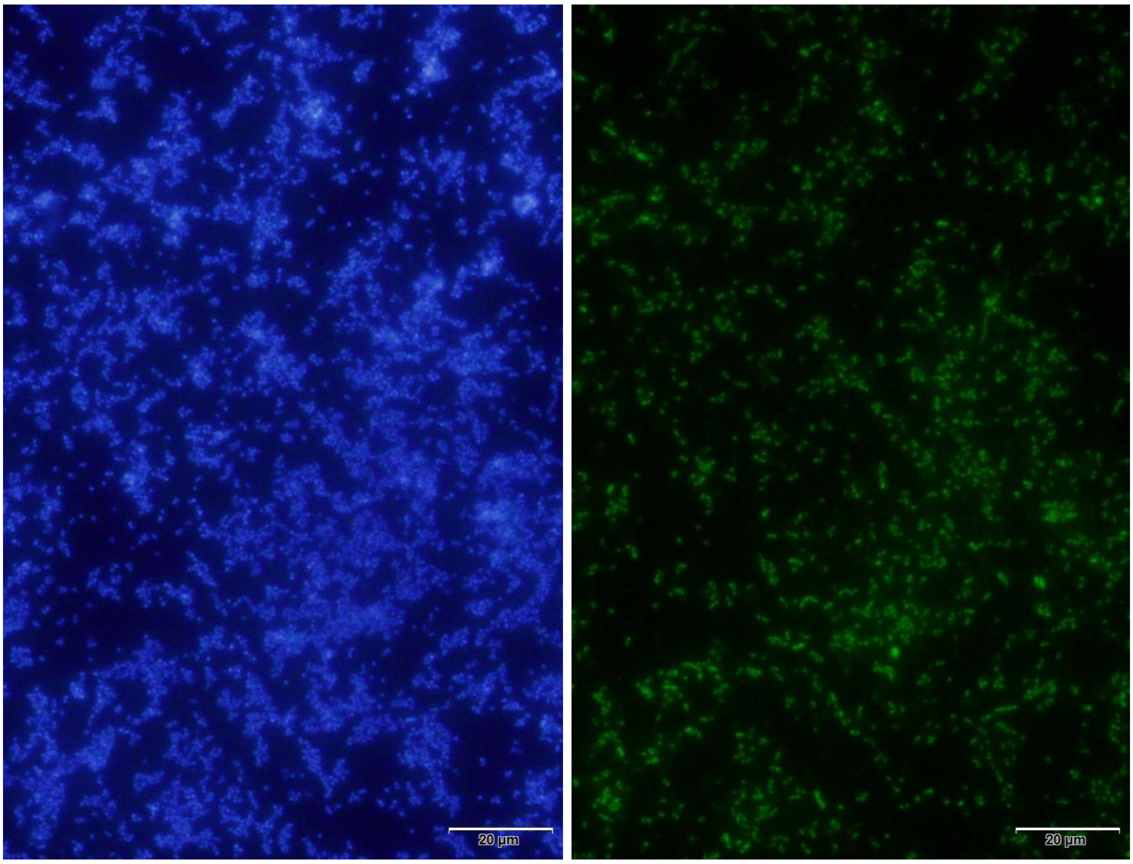

Prevotella bivia CCUG 35880

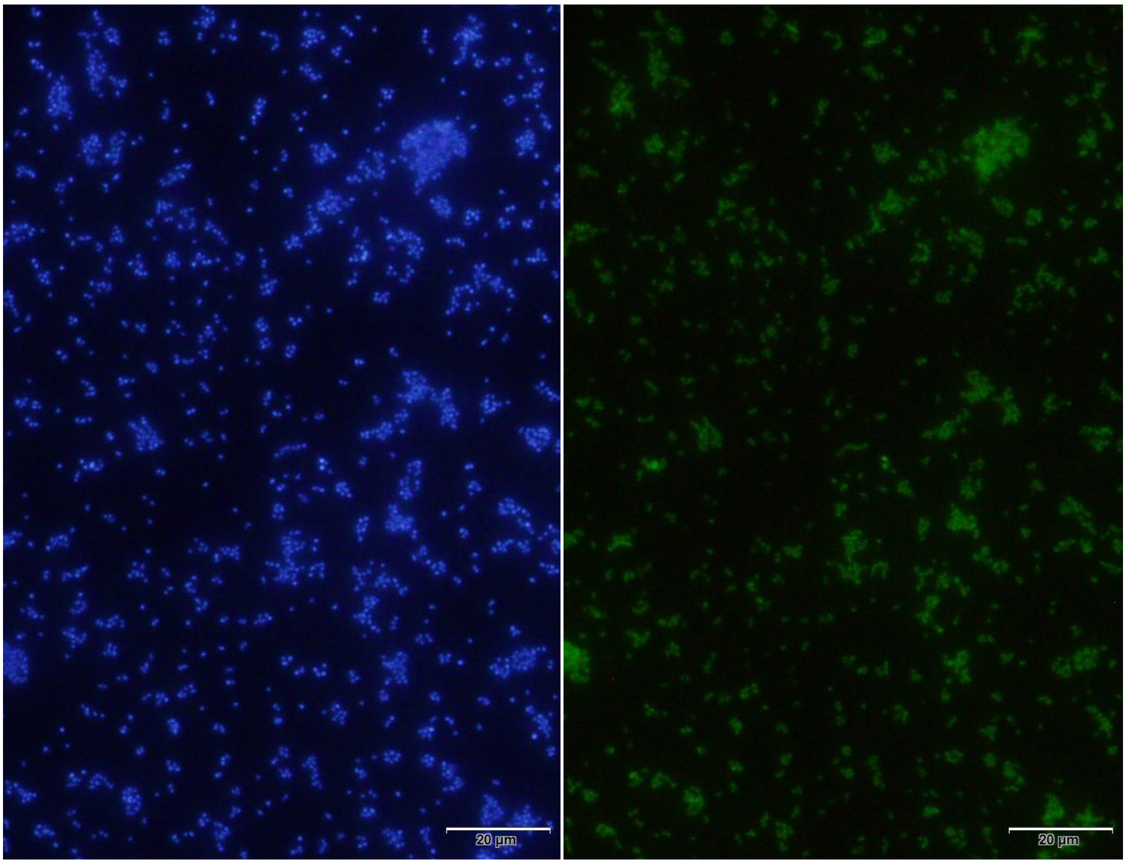

Prevotella bivia CCUG 36740

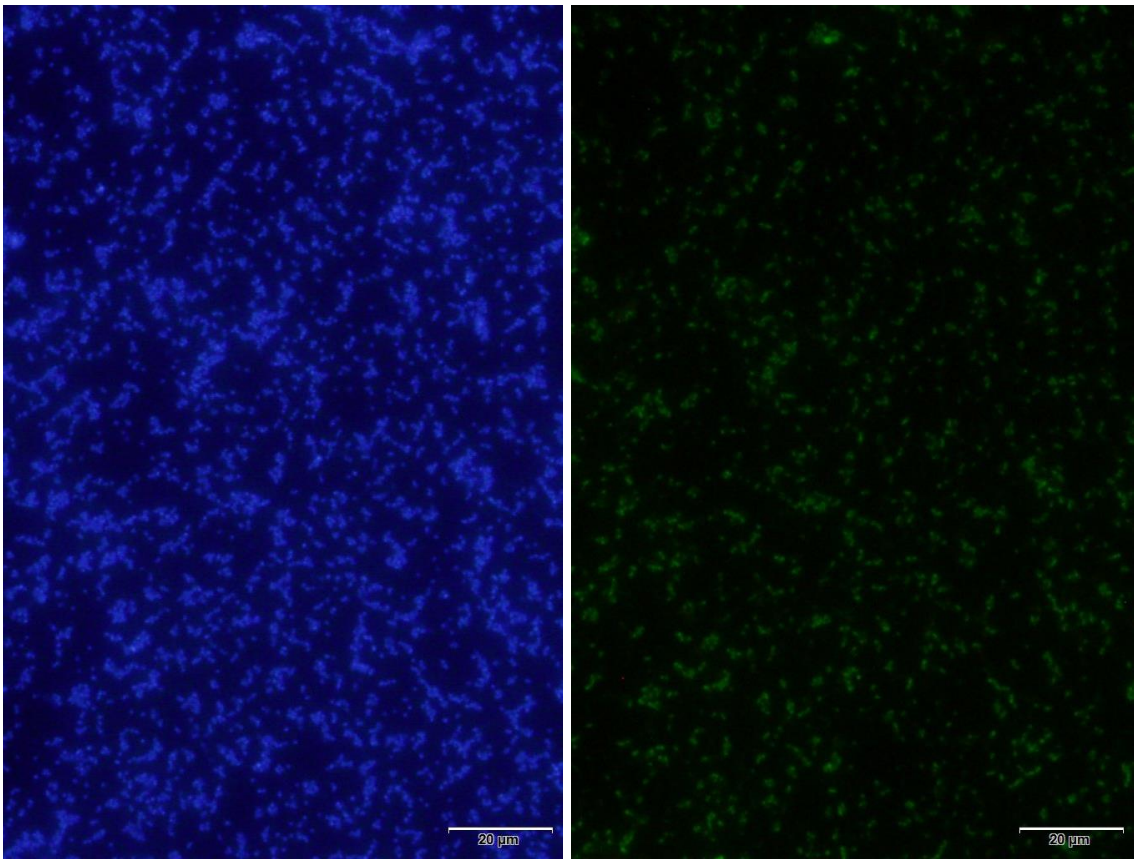

Prevotella bivia CCUG 44195

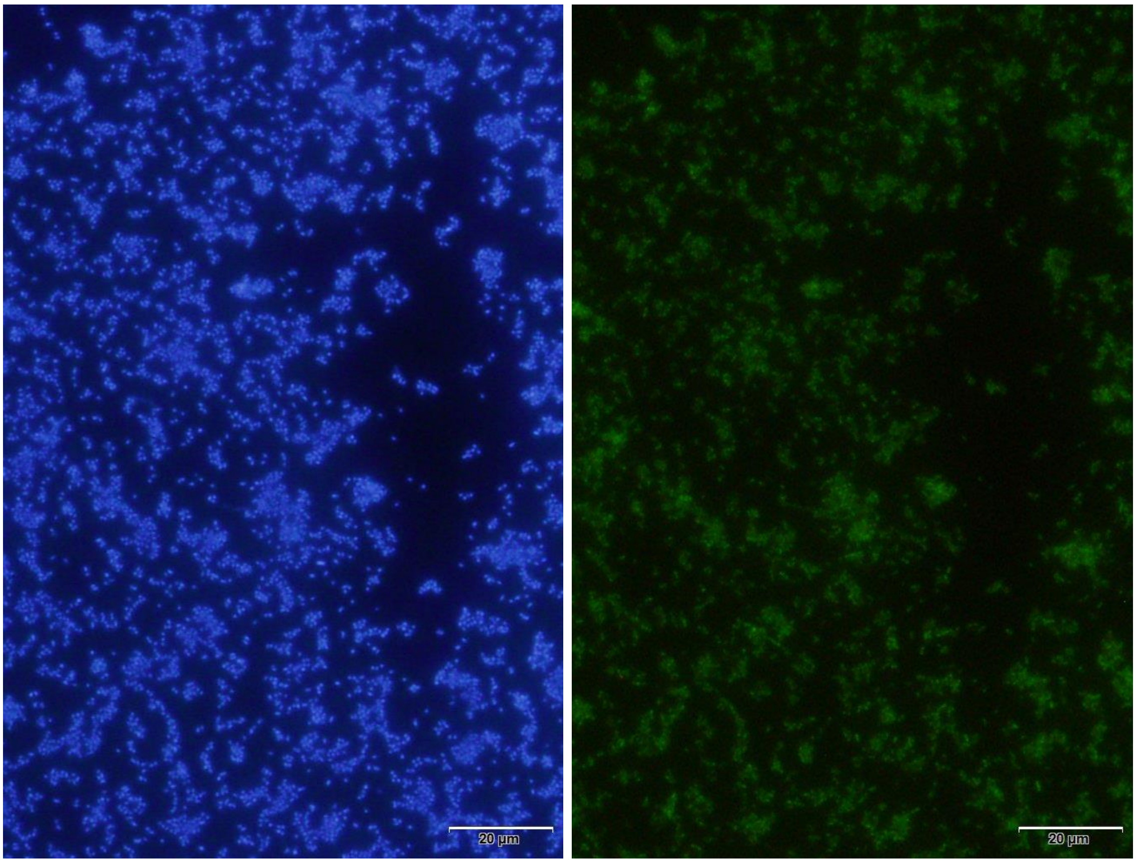

Prevotella bivia CCUG 48913

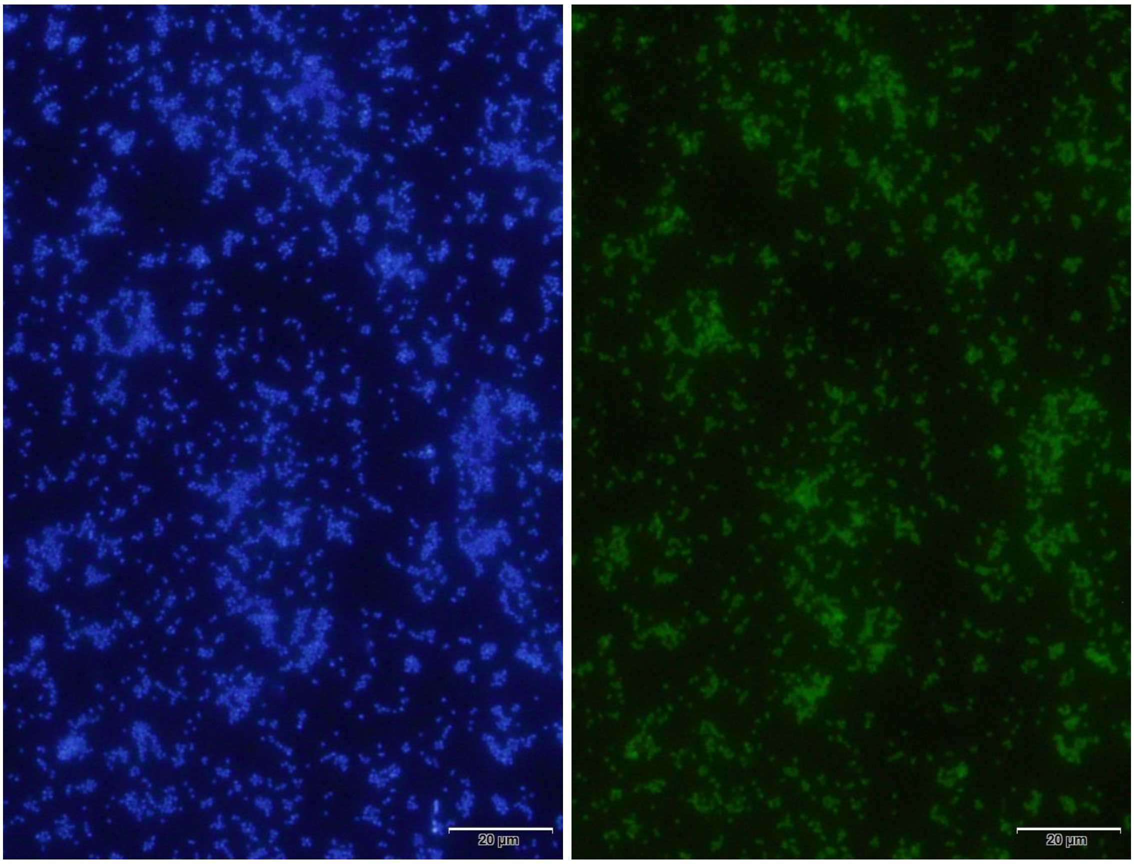

Prevotella bivia CCUG 56865

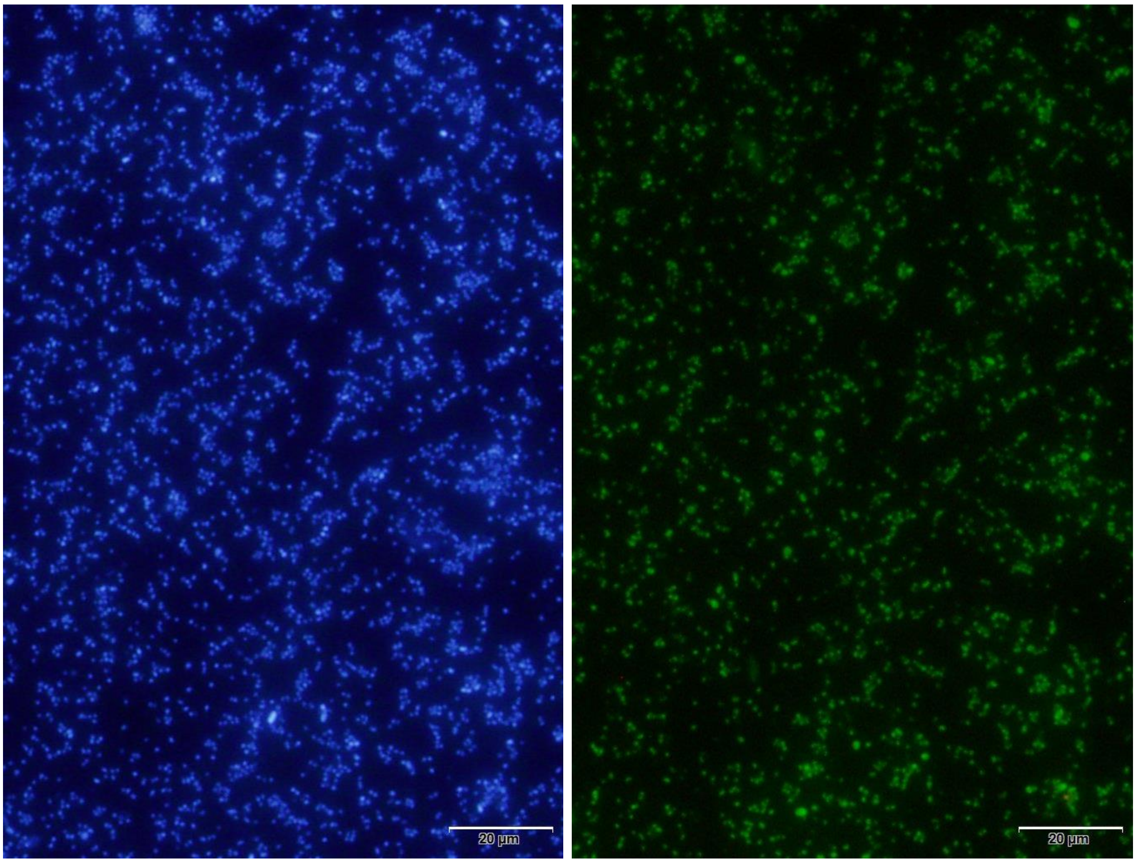

Prevotella bivia CCUG 59496

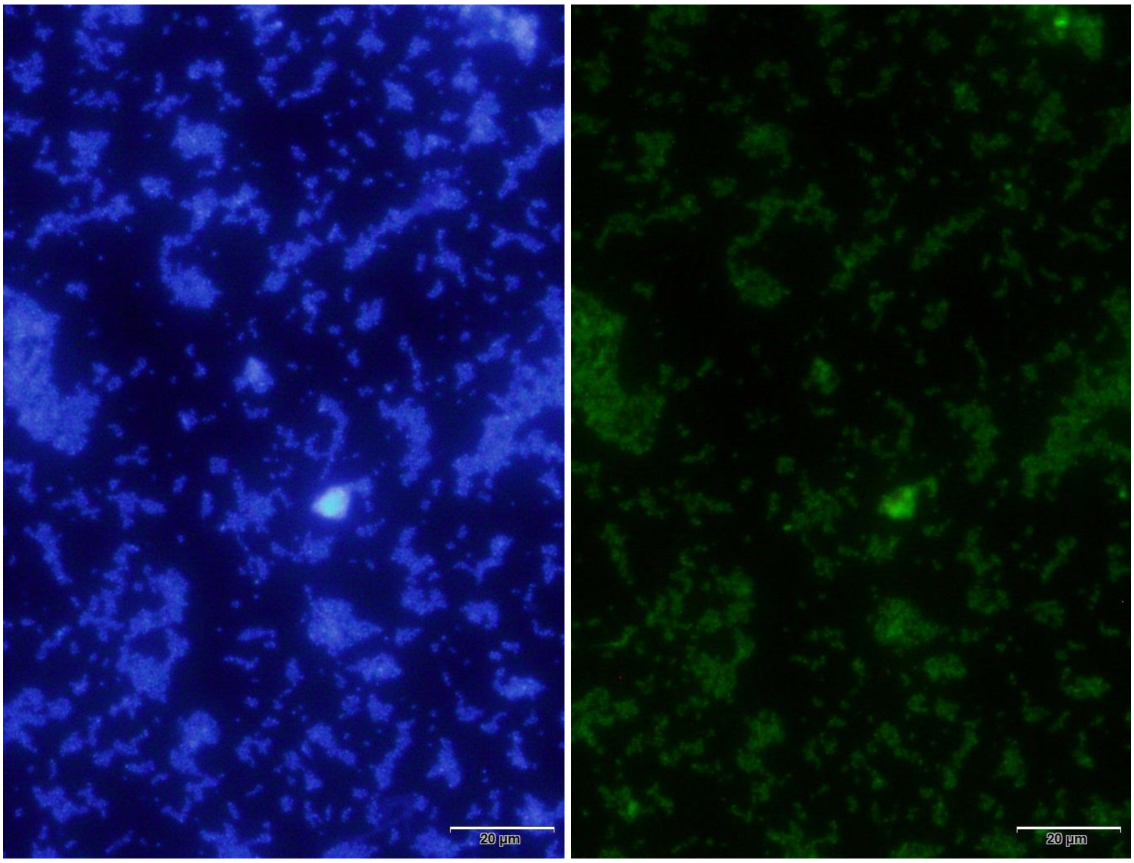

***Acinetobacter baumannii* CCUG 59798**

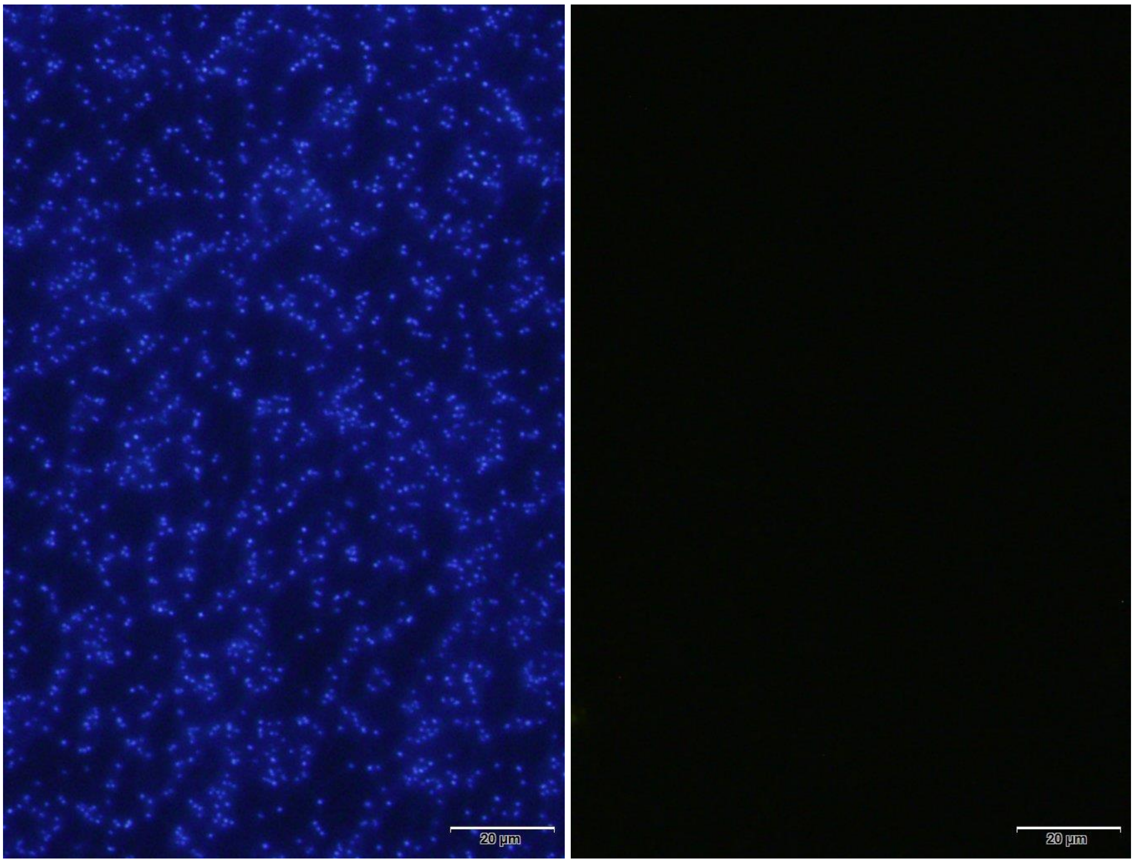

***Actinomyces neuii* UM067**

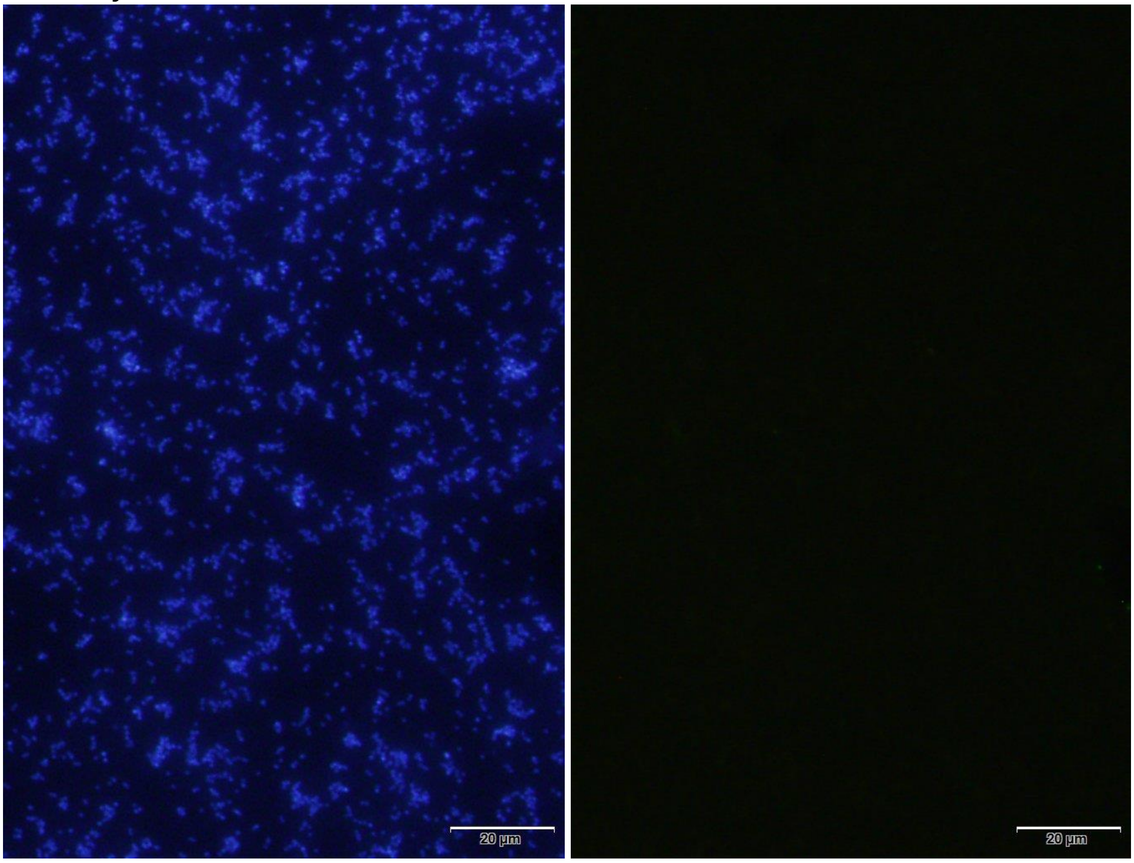

***Actinomyces urogenitalis* CCUG 44038**

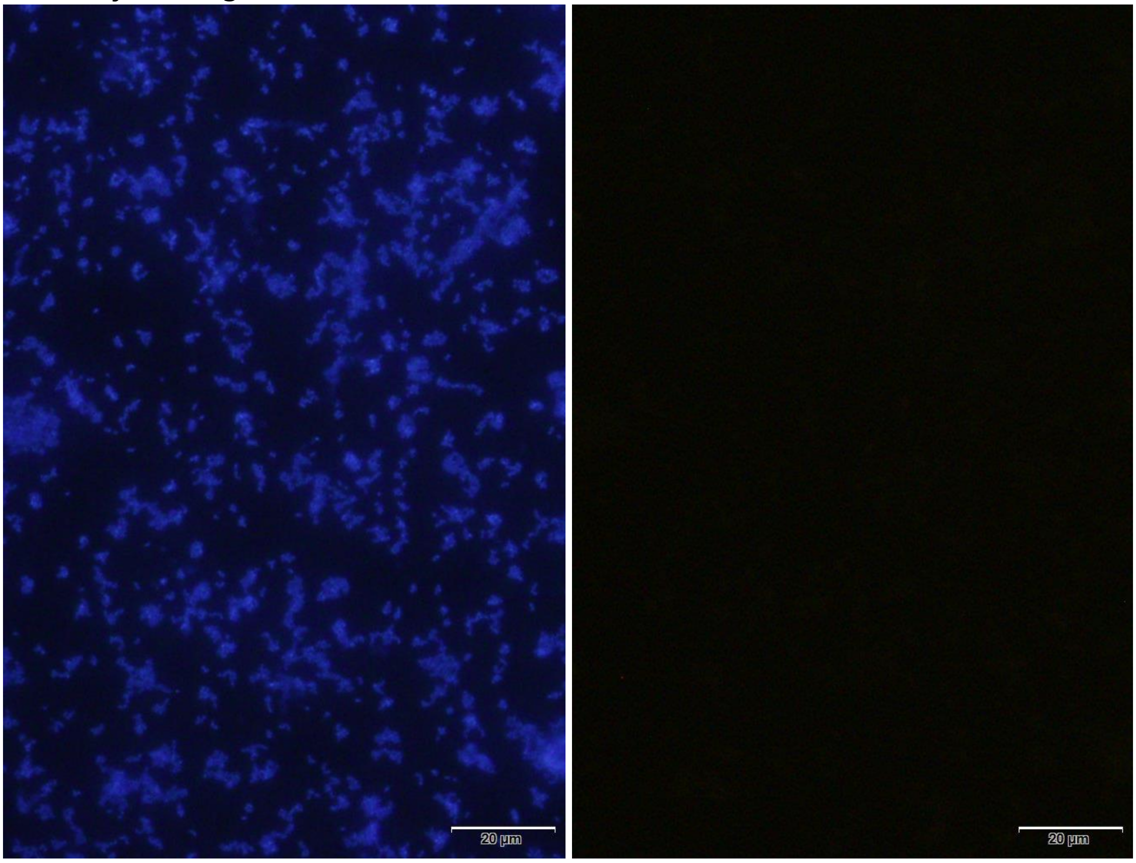

***Aerococcus christensenii* CCUG 28826**

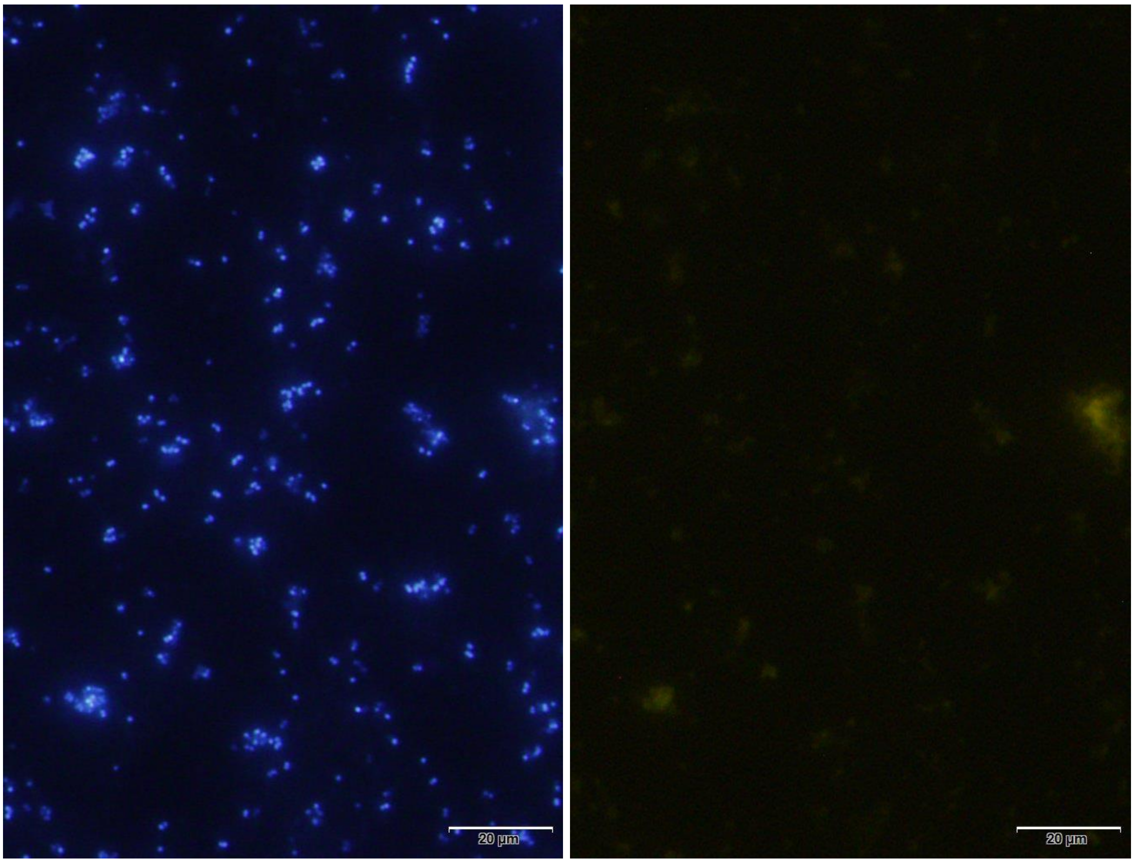

Bacillus firmus **UM034**

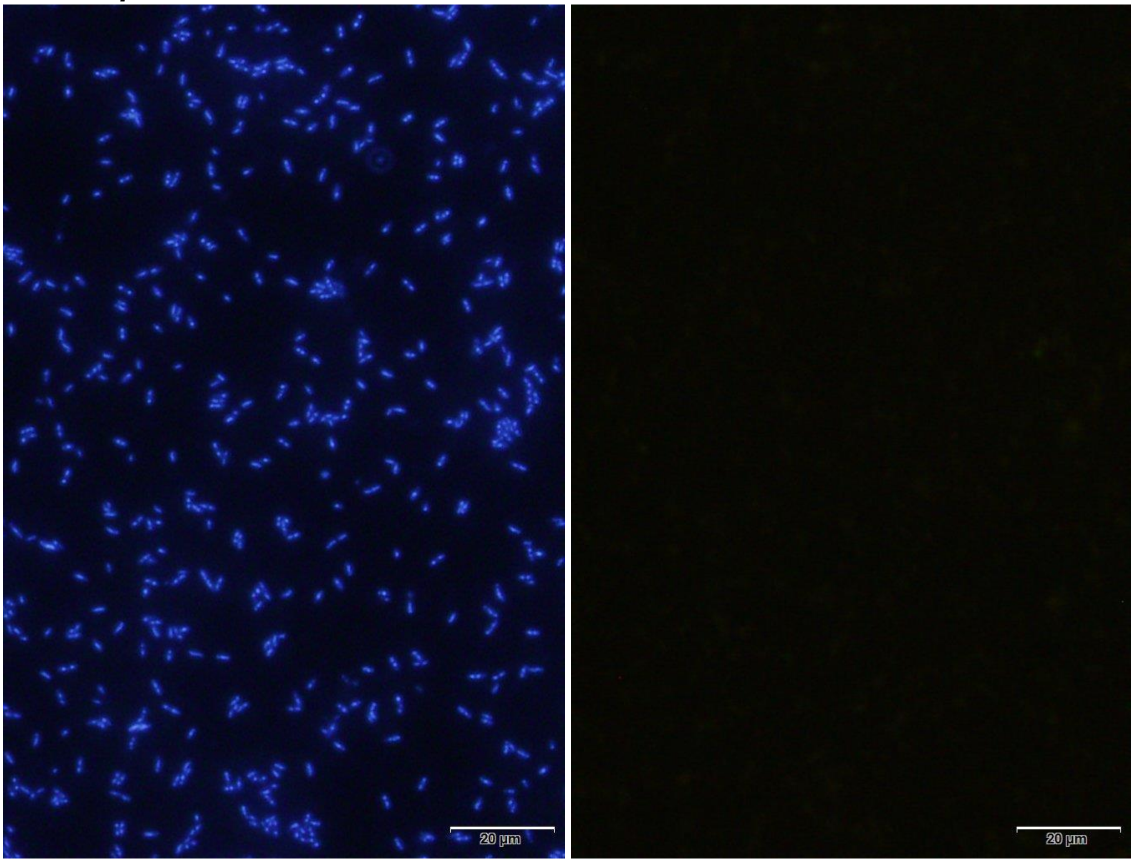

Bifidobacterium bifidum **CCUG 59492**

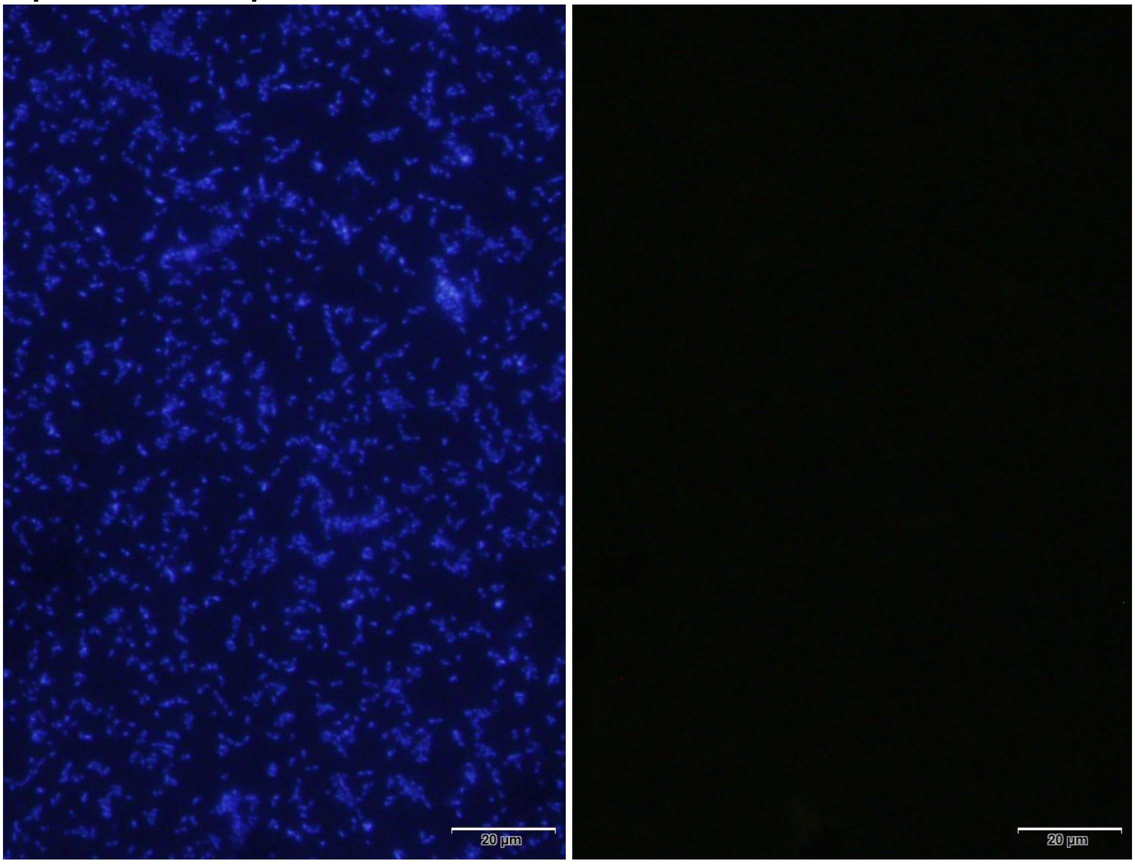

***Brevibacterium ravenspurgense* CCUG 42923**

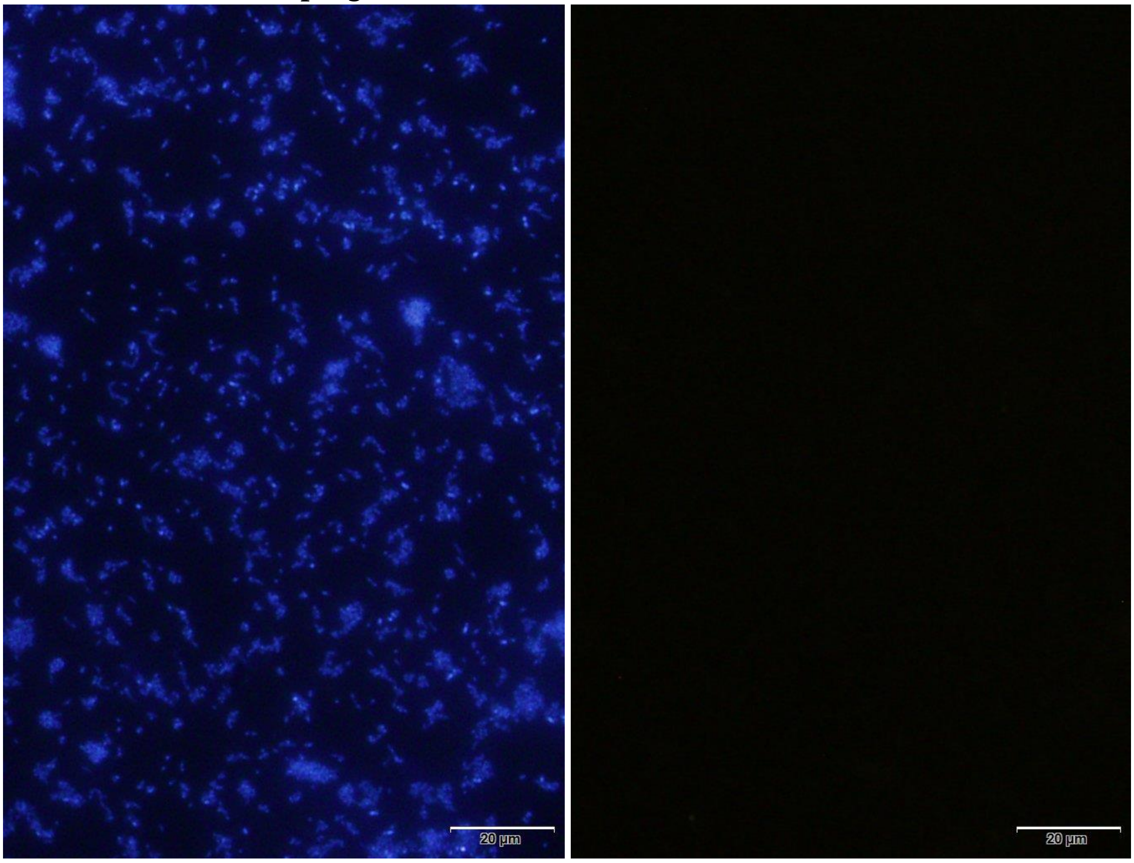

***Campylobacter ureolyticus* CCUG 44295**

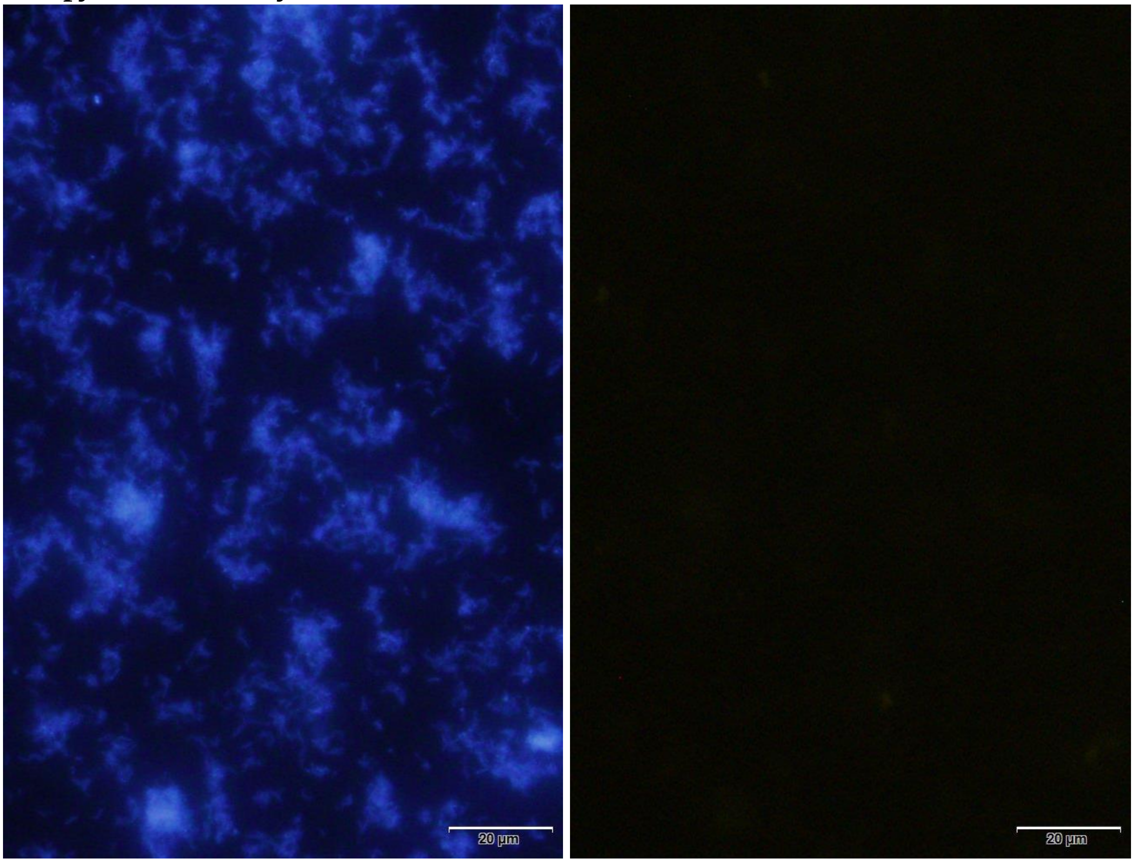

Corynebacterium tuscaniense UM137

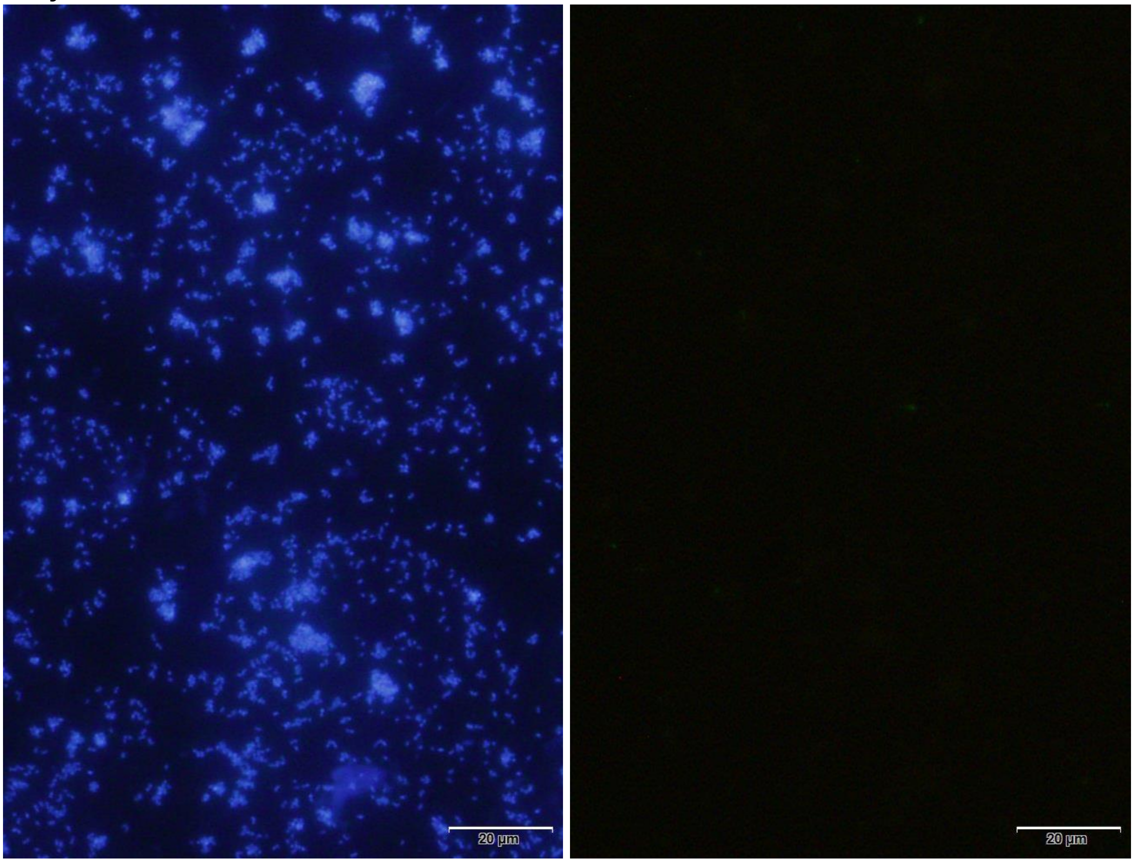

Enterococcus faecalis UM035

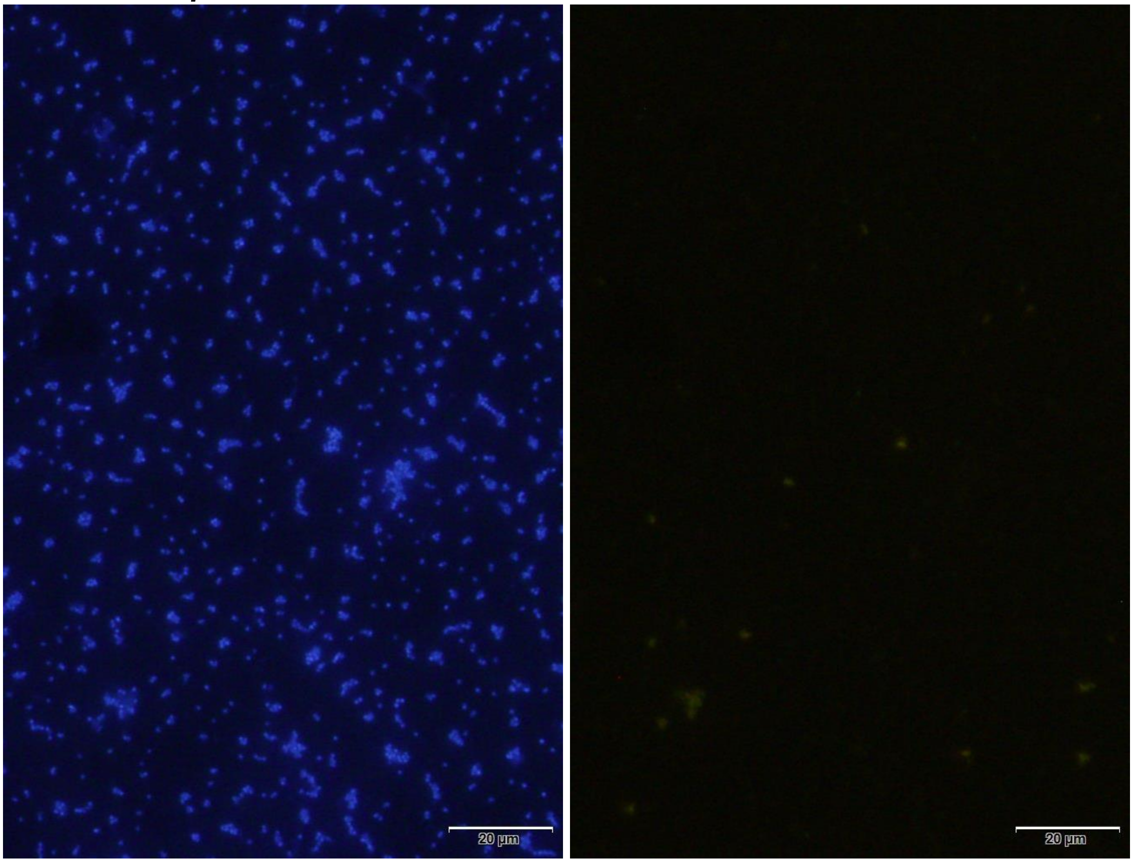

Escherichia coli UM056

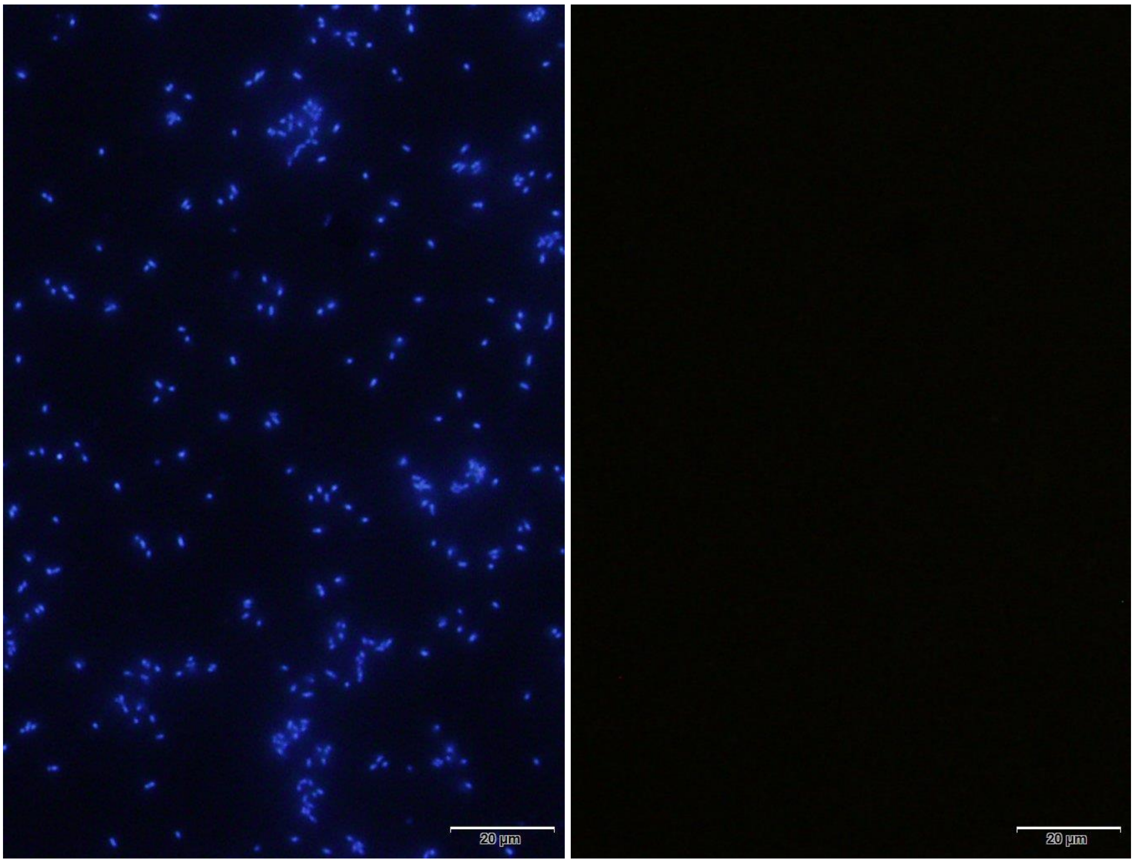

Gardnerella leopoldii UM034

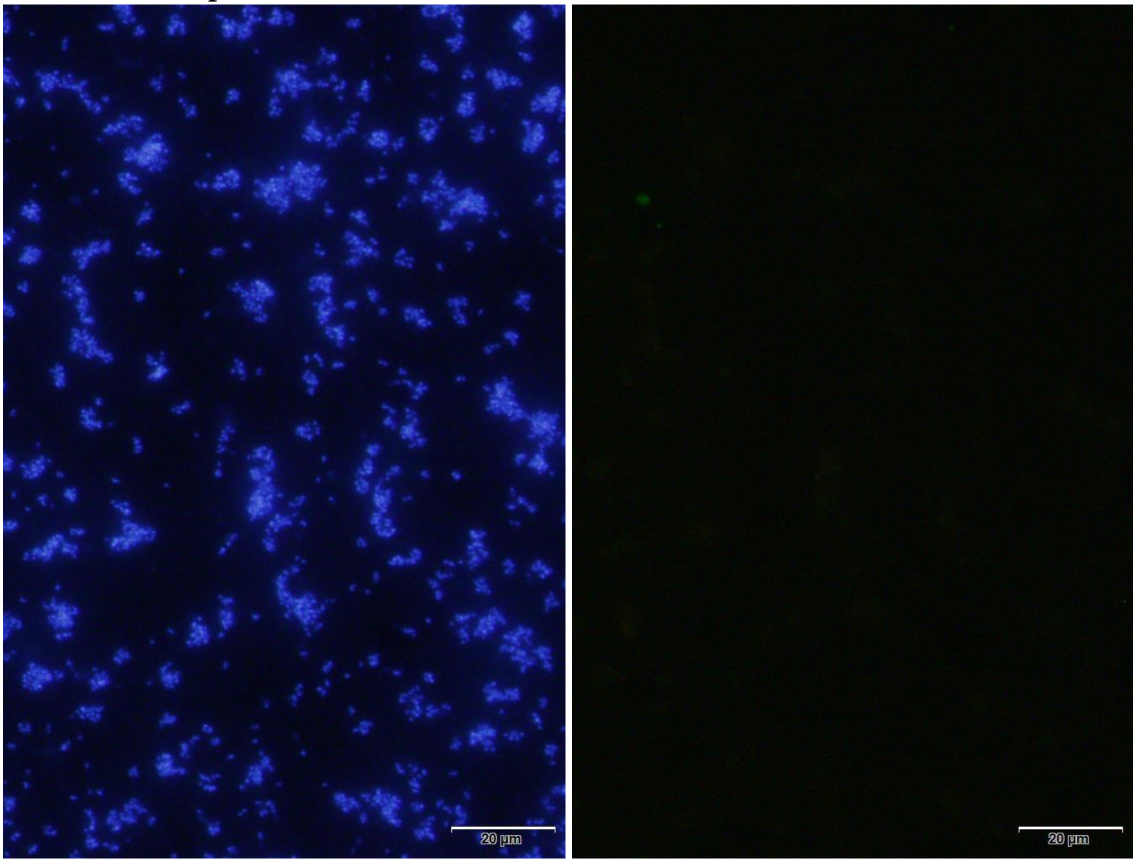

Gardnerella piotii UM035

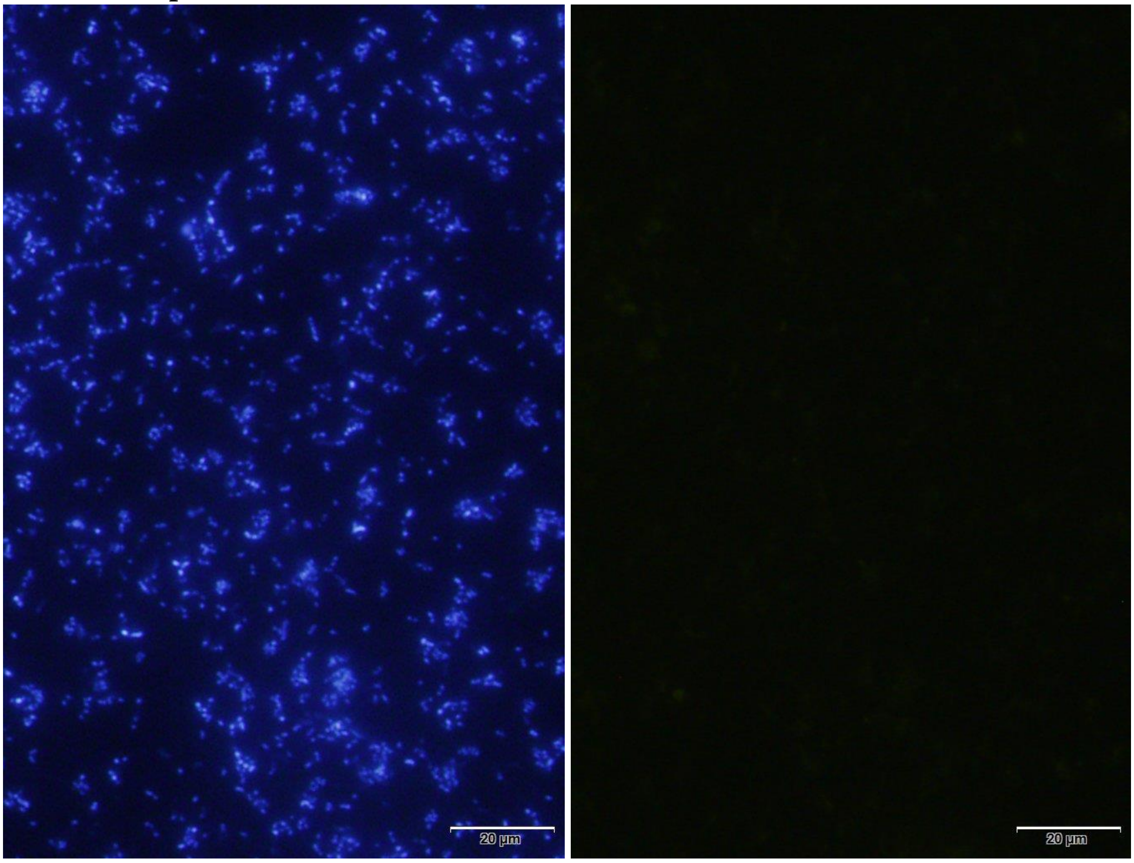

Gardnerella swidsinskii UM094

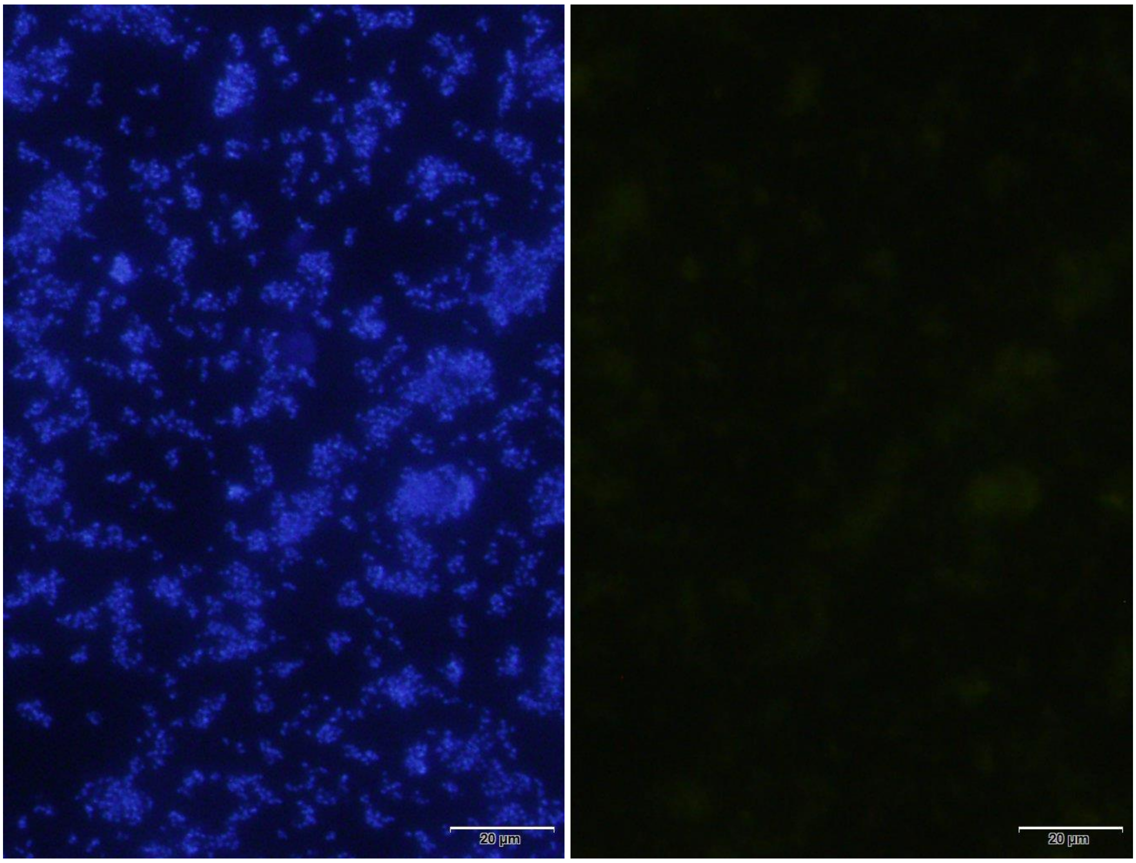

***Gemella haemolysans* UM034**

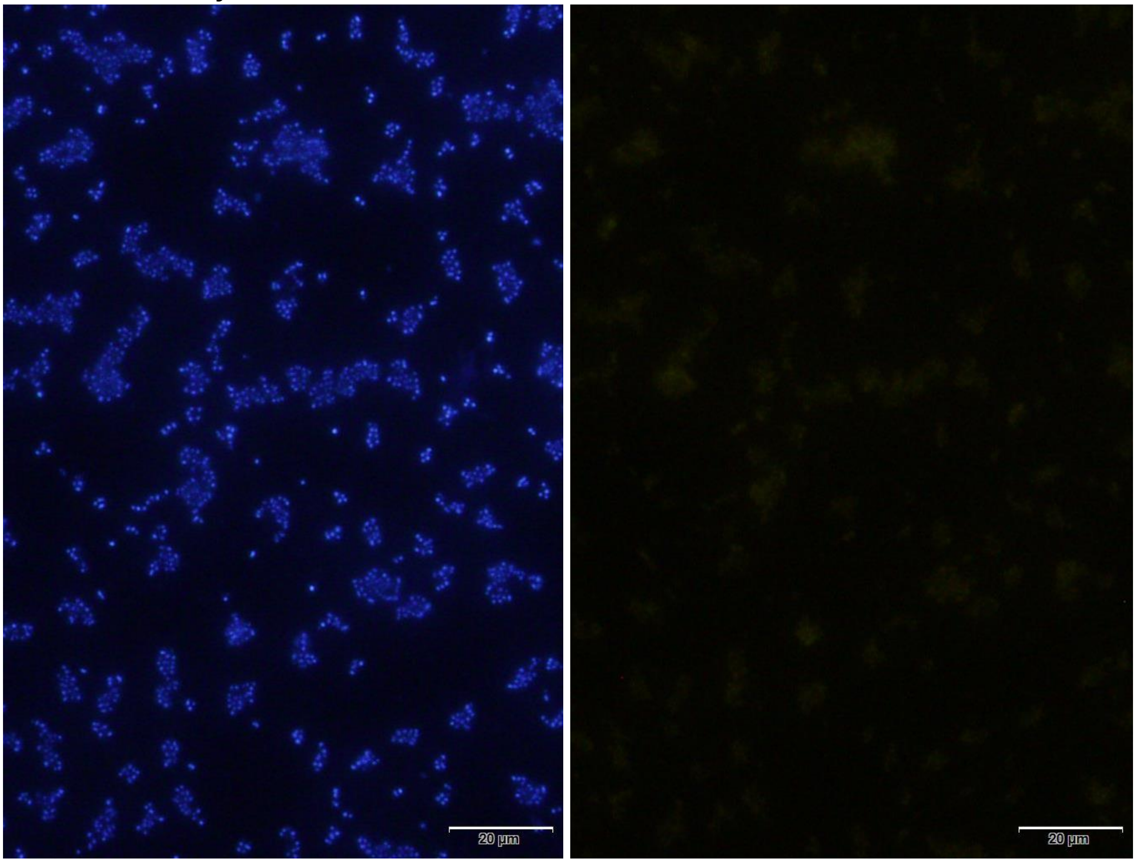

***Lactobacillus crispatus* EX533959VCO6**

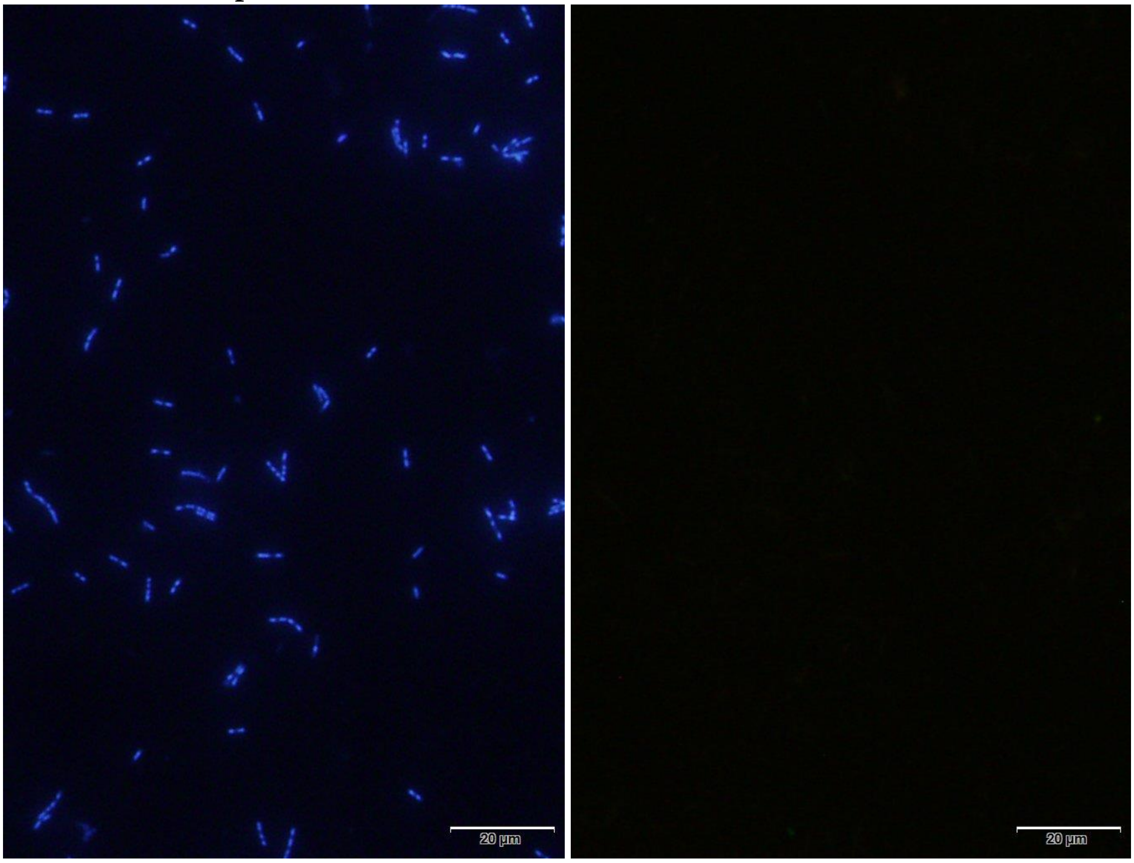

Lactobacillus gasseri **ATCC 9857**

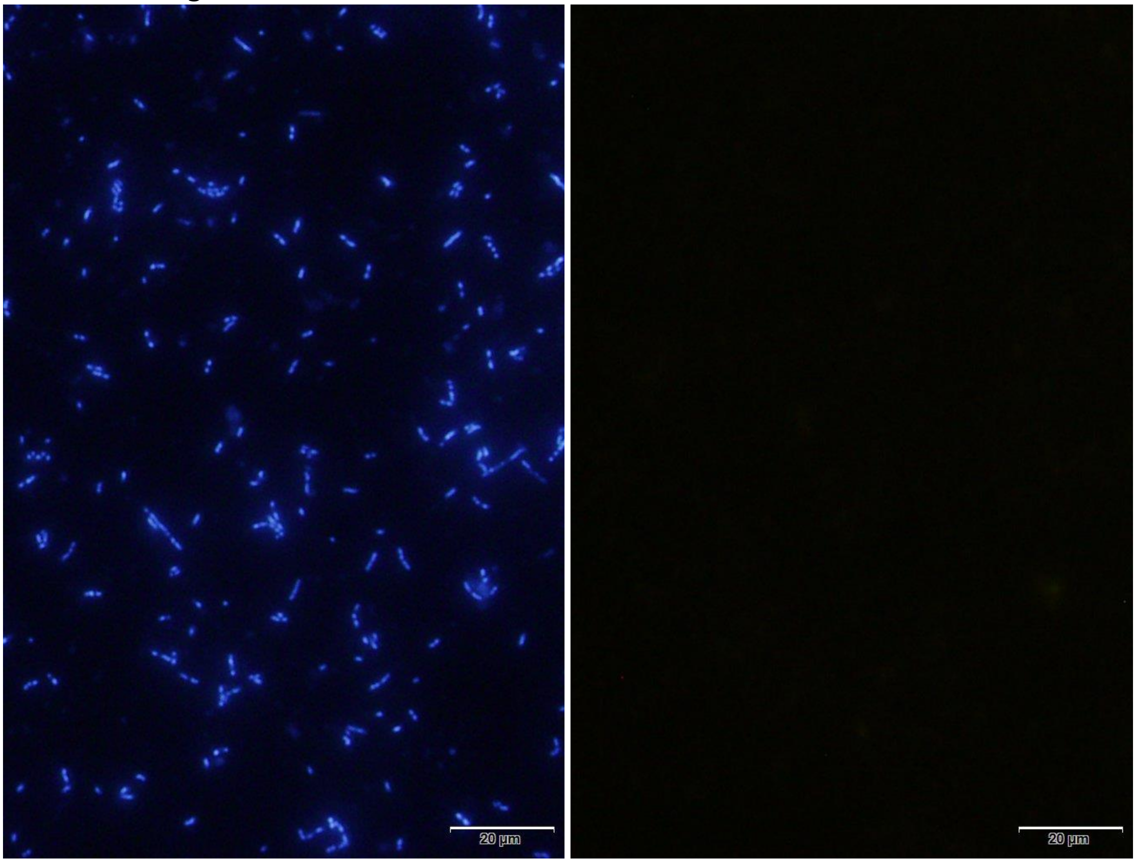

Lactobacillus iners **ATCC 55195**

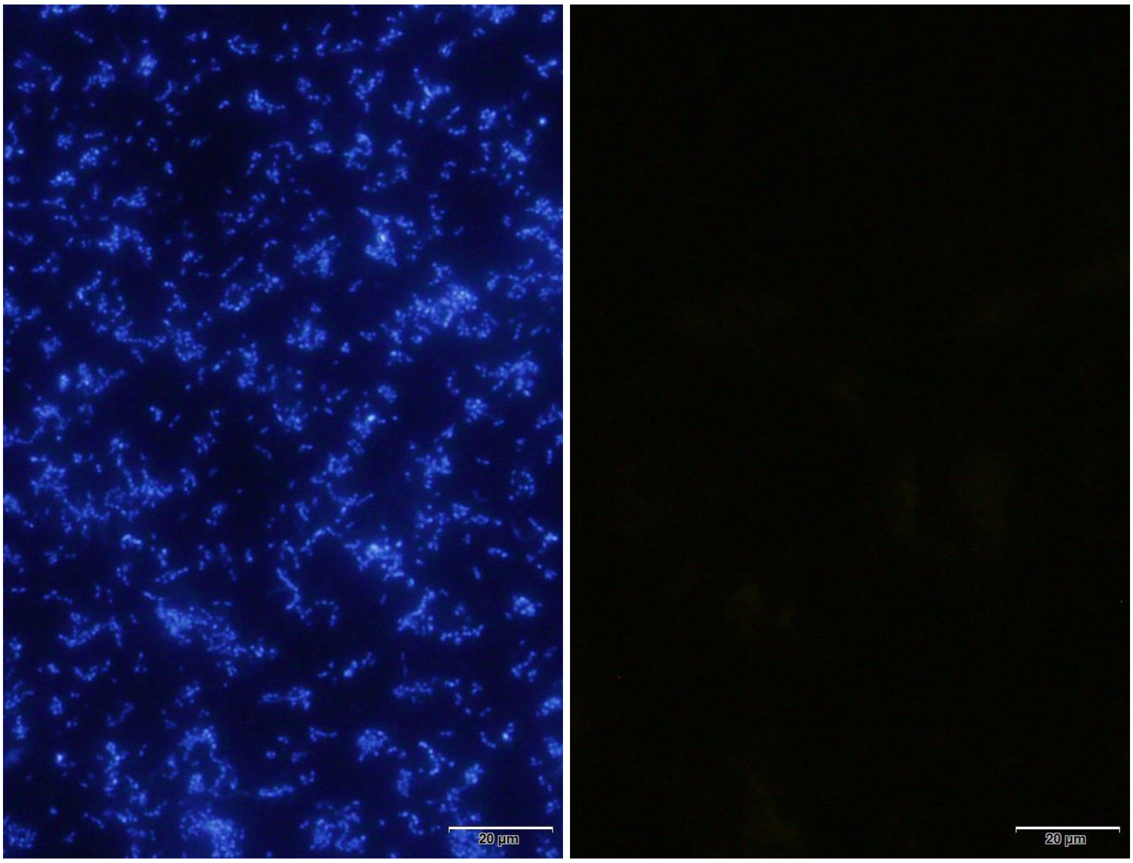

***Lactobacillus rhamnosus* CECT 288**

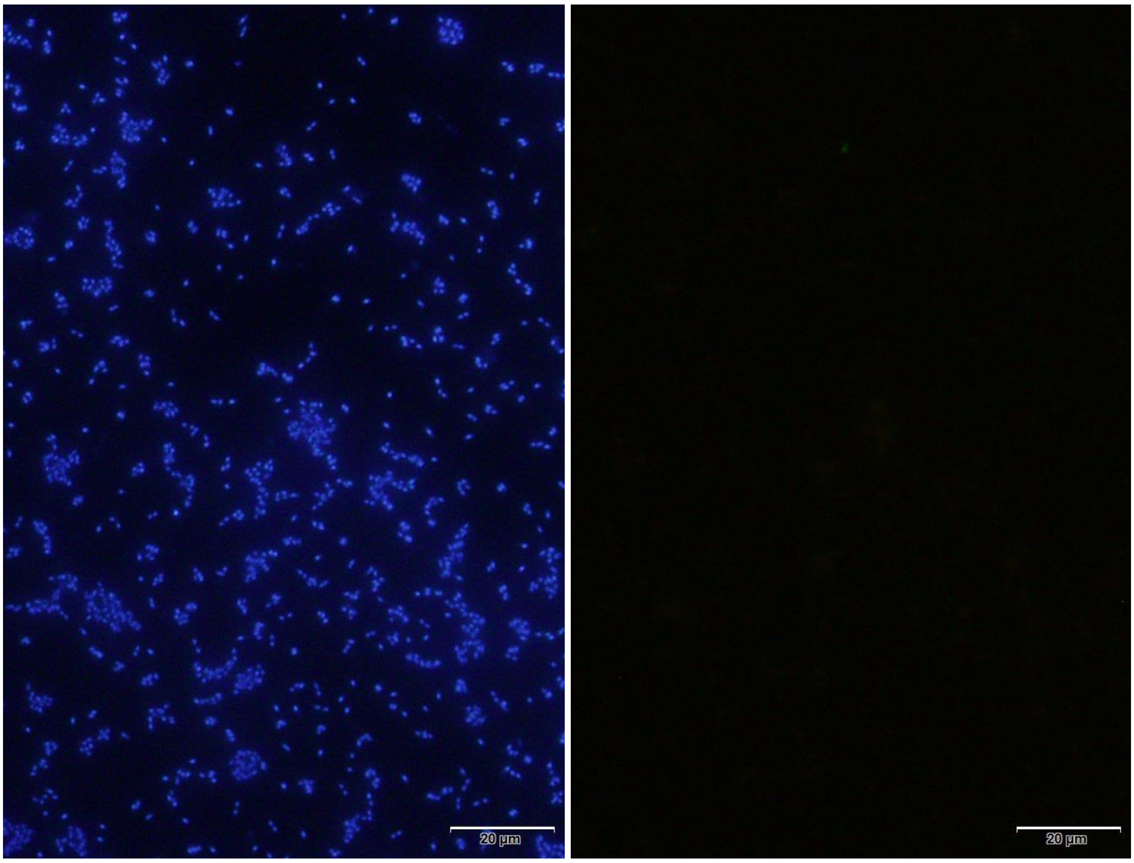

***Lactobacillus vaginalis* UM062**

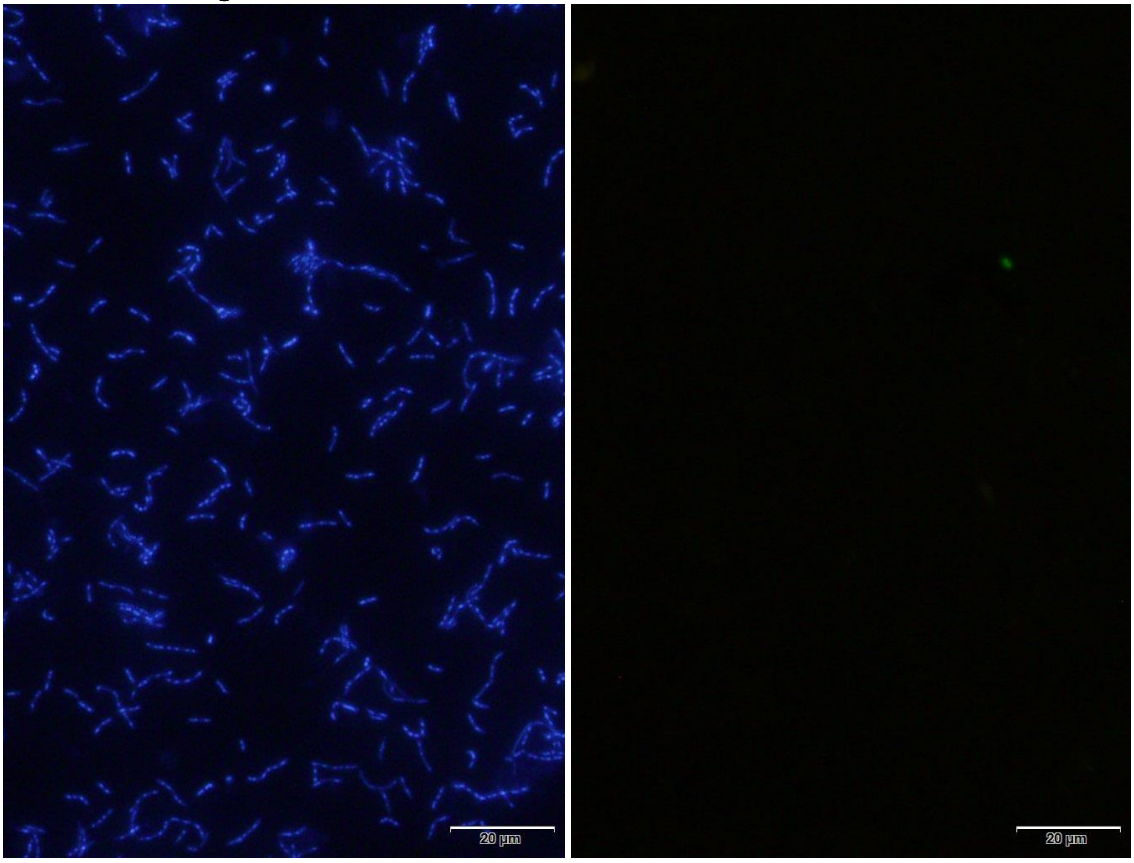

Megasphaera micronuciformis CCUG 45952

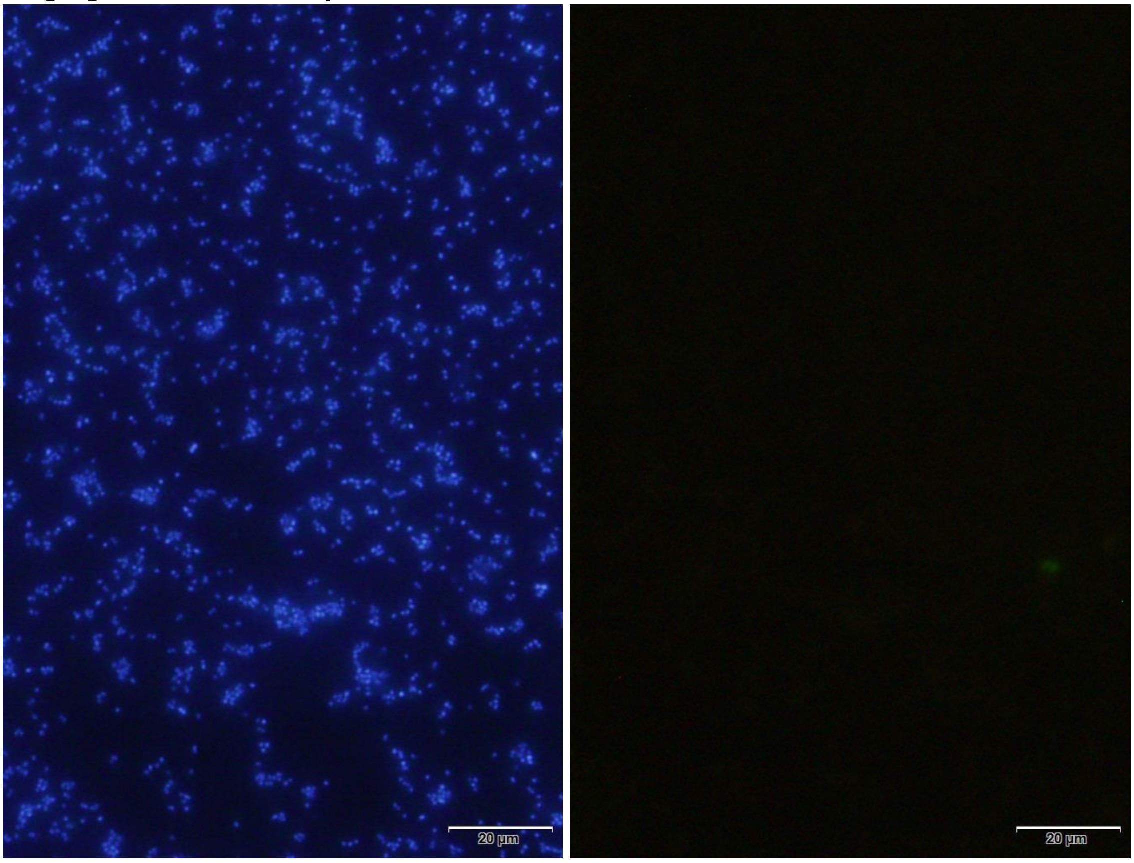

Mobiluncus curtisii ATCC 35241

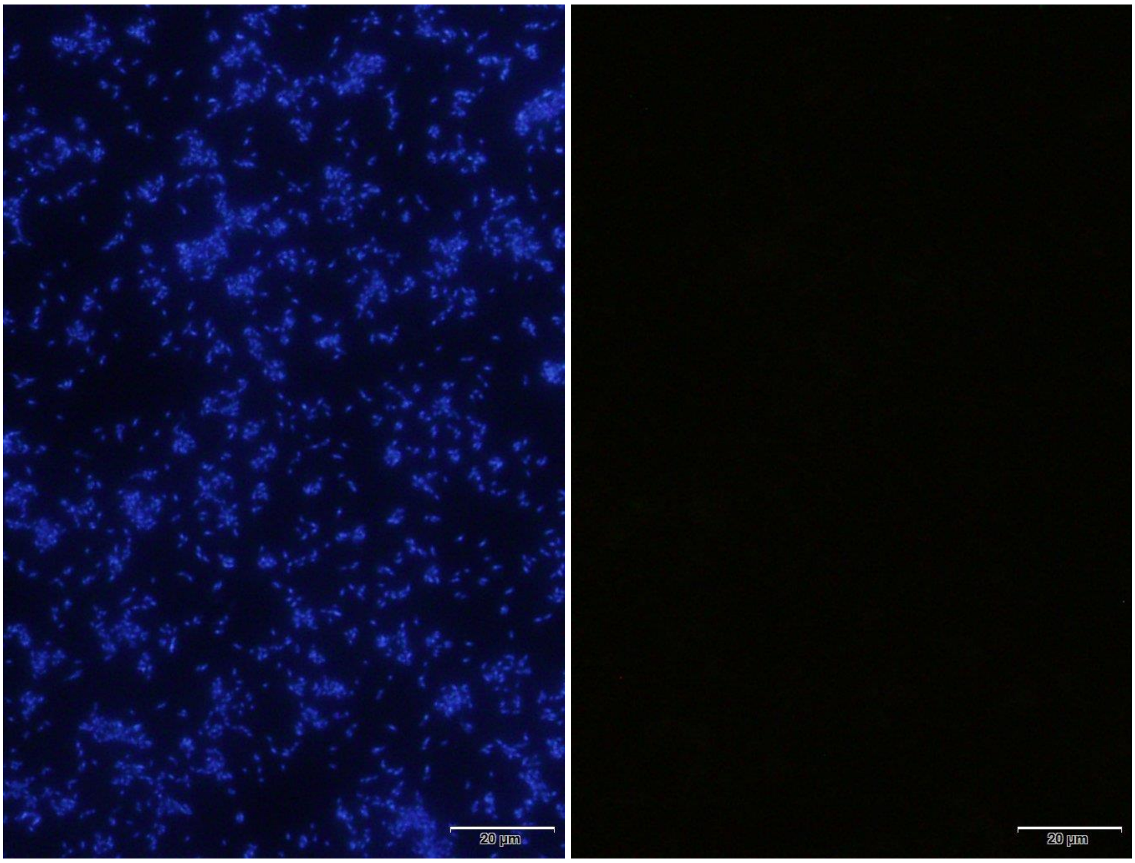

***Mobiluncus mulieris* ATCC 35239**

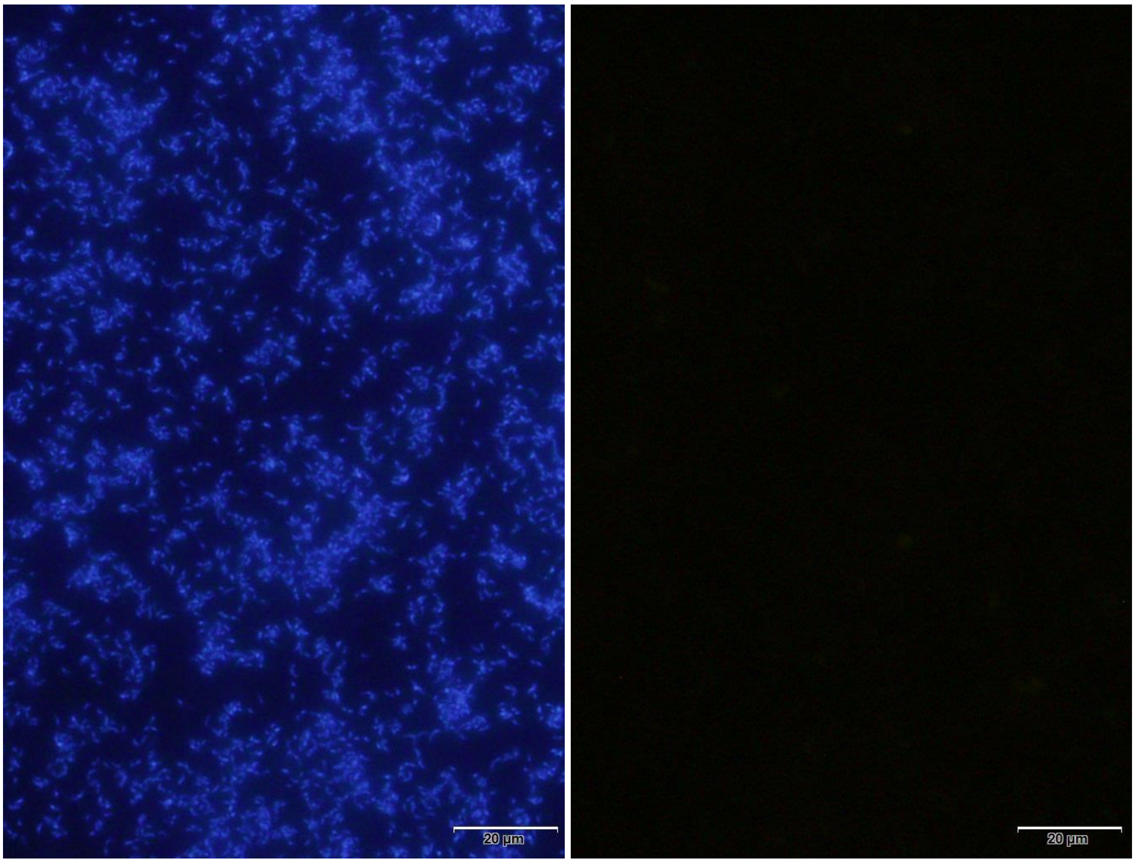

***Mycoplasma hominis* UM054**

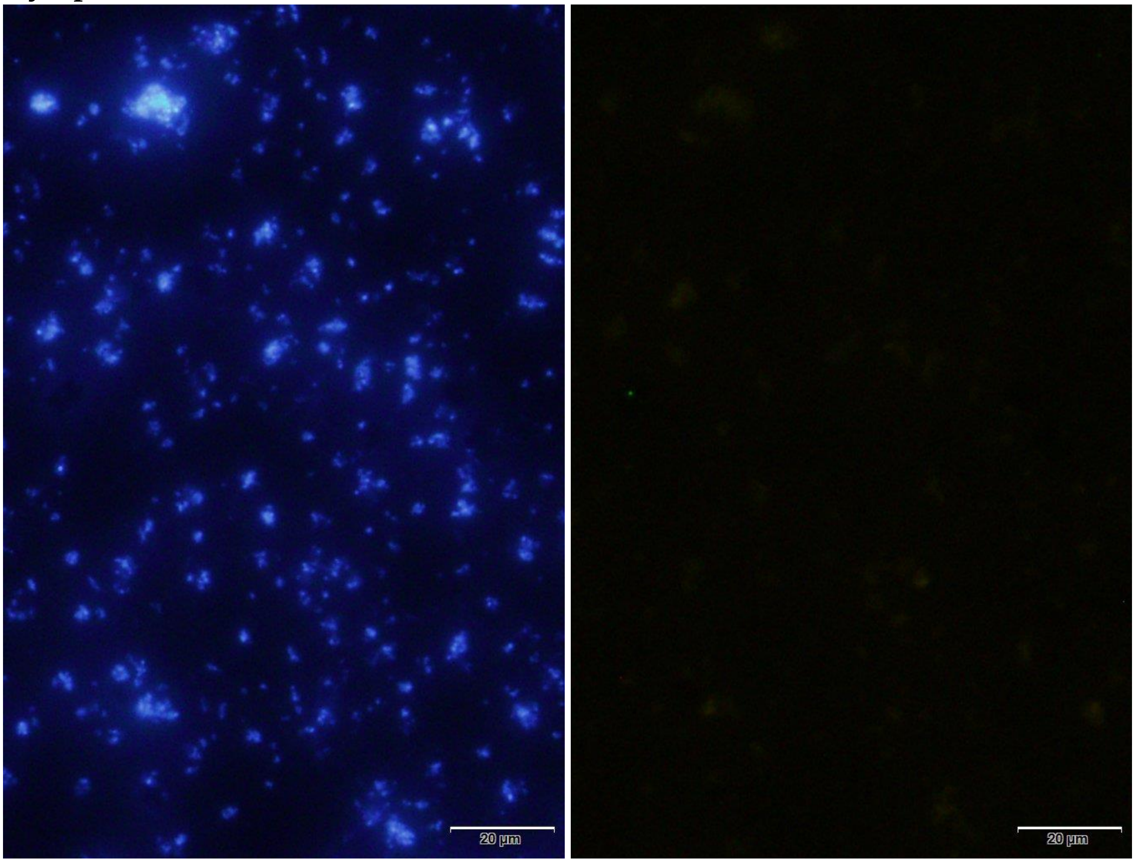

***Neisseria gonorrhoeae* CCUG 13281**

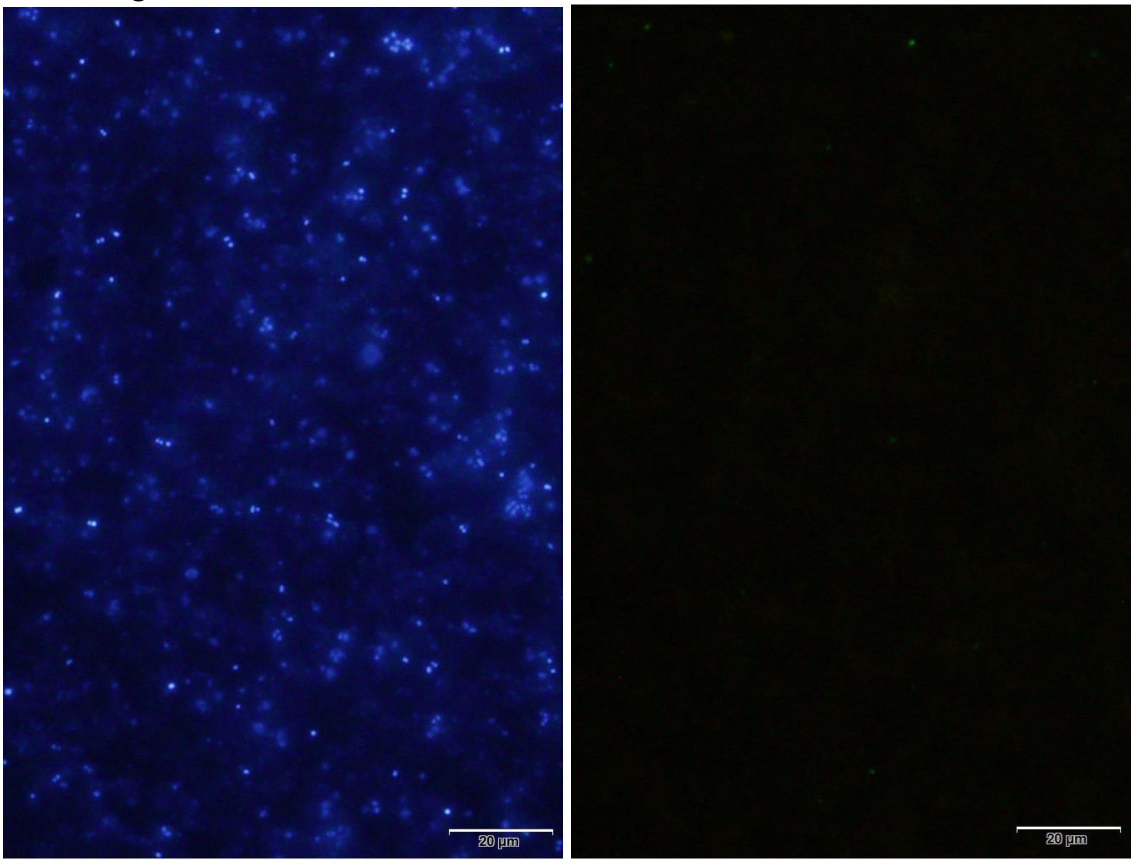

***Nosocomiicoccus ampullae* UM121**

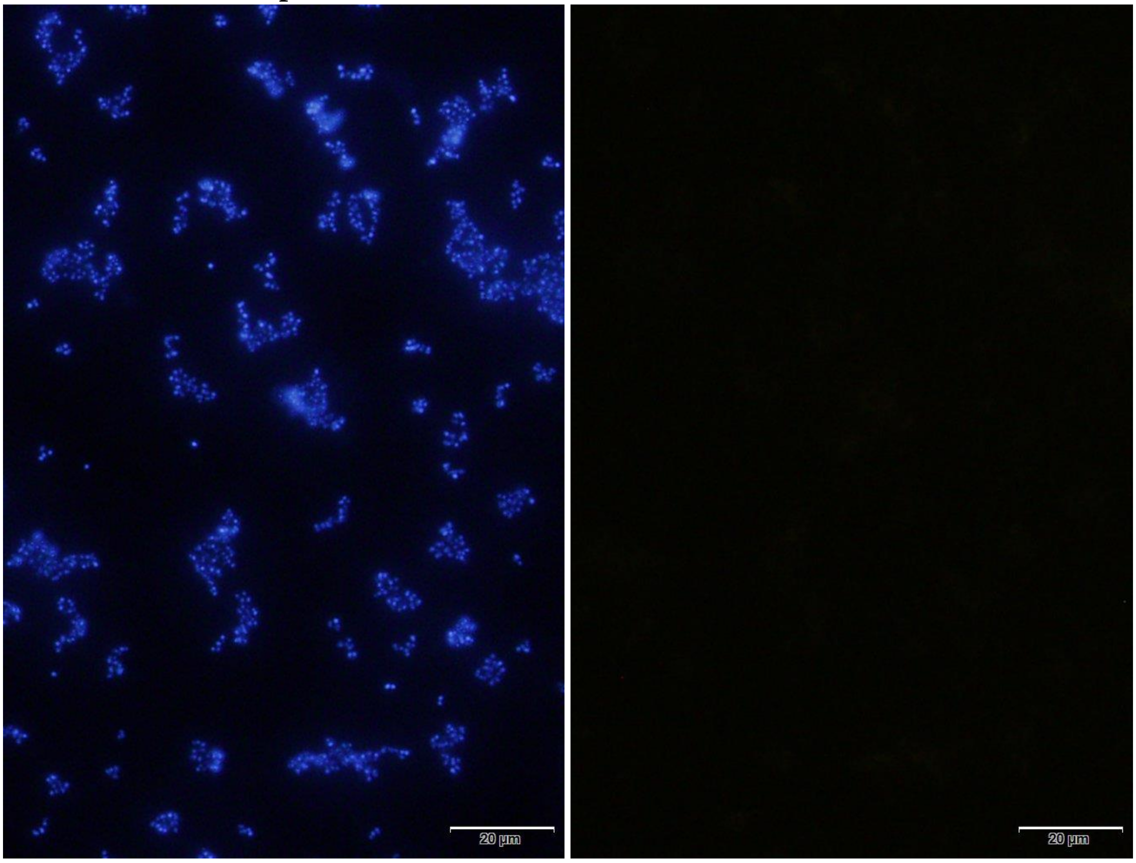

***Peptostreptococcus anaerobius* ATCC 27337**

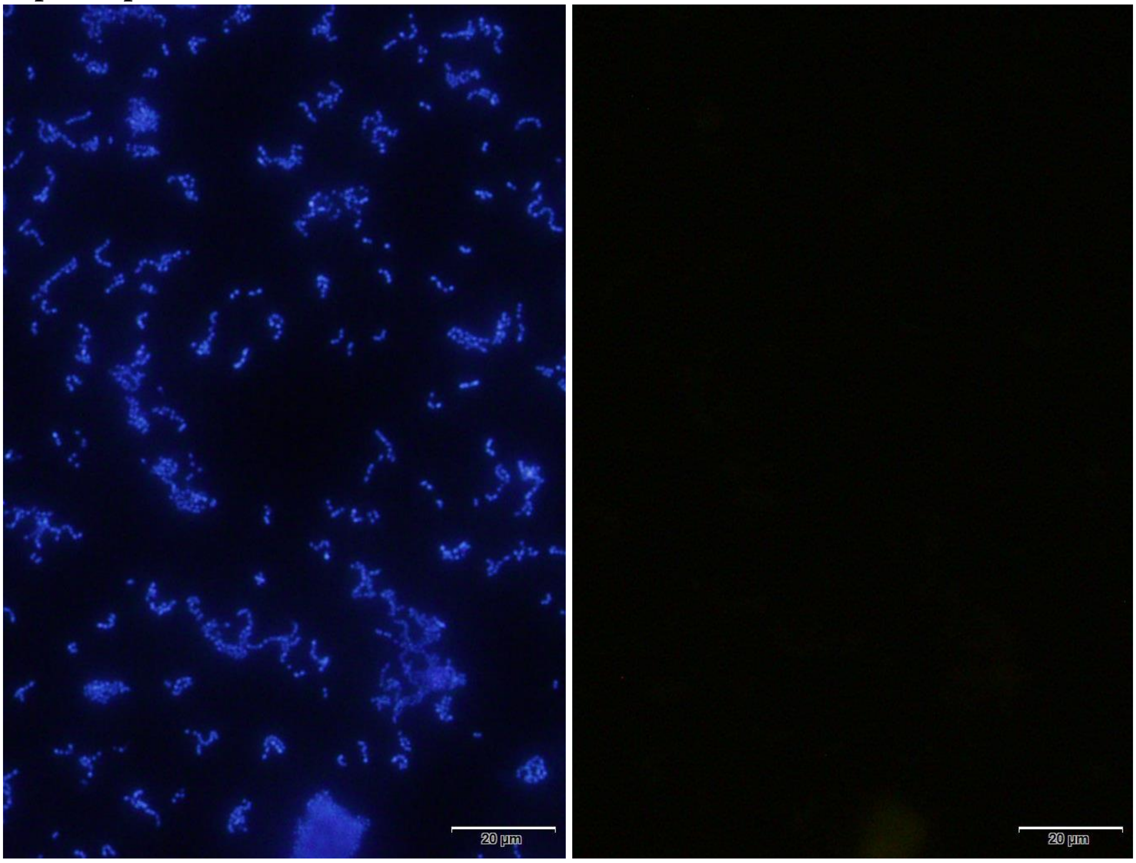

***Porphyromonas asaccharolytica* CCUG 7834**

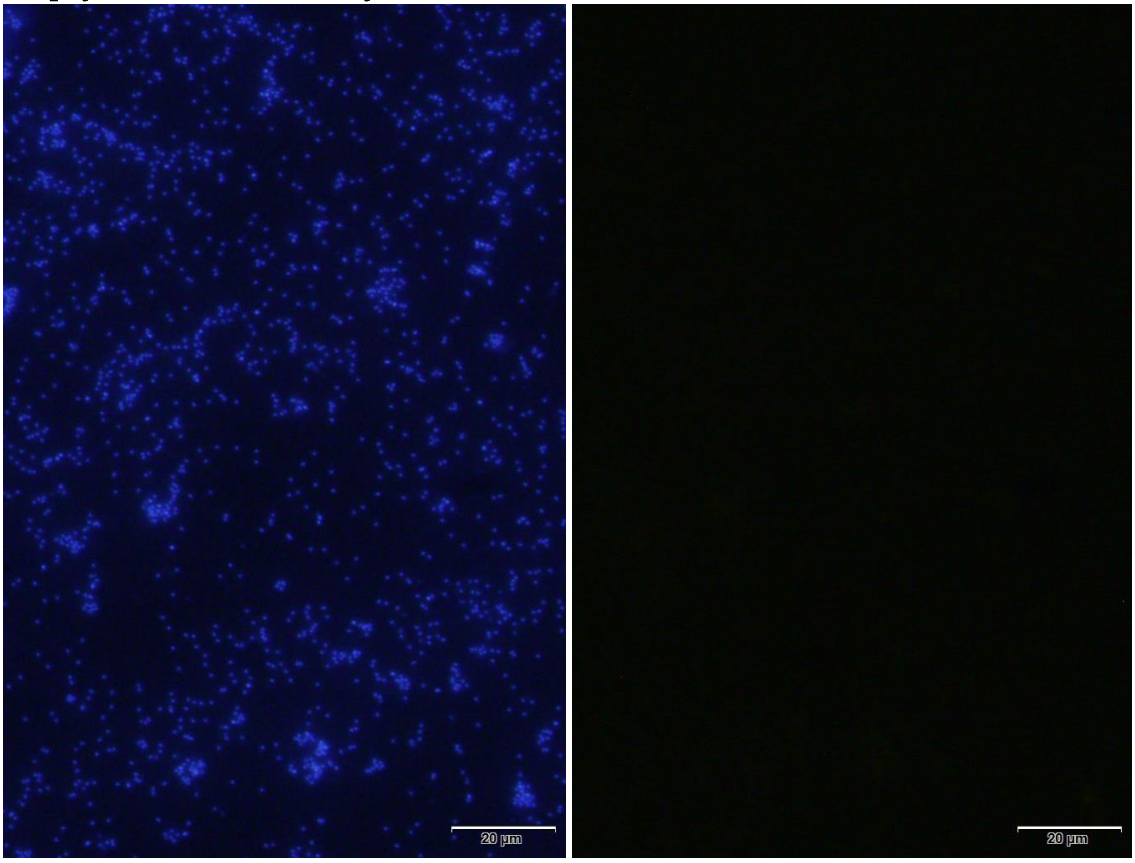

Prevotella buccalis **CCUG 44127**

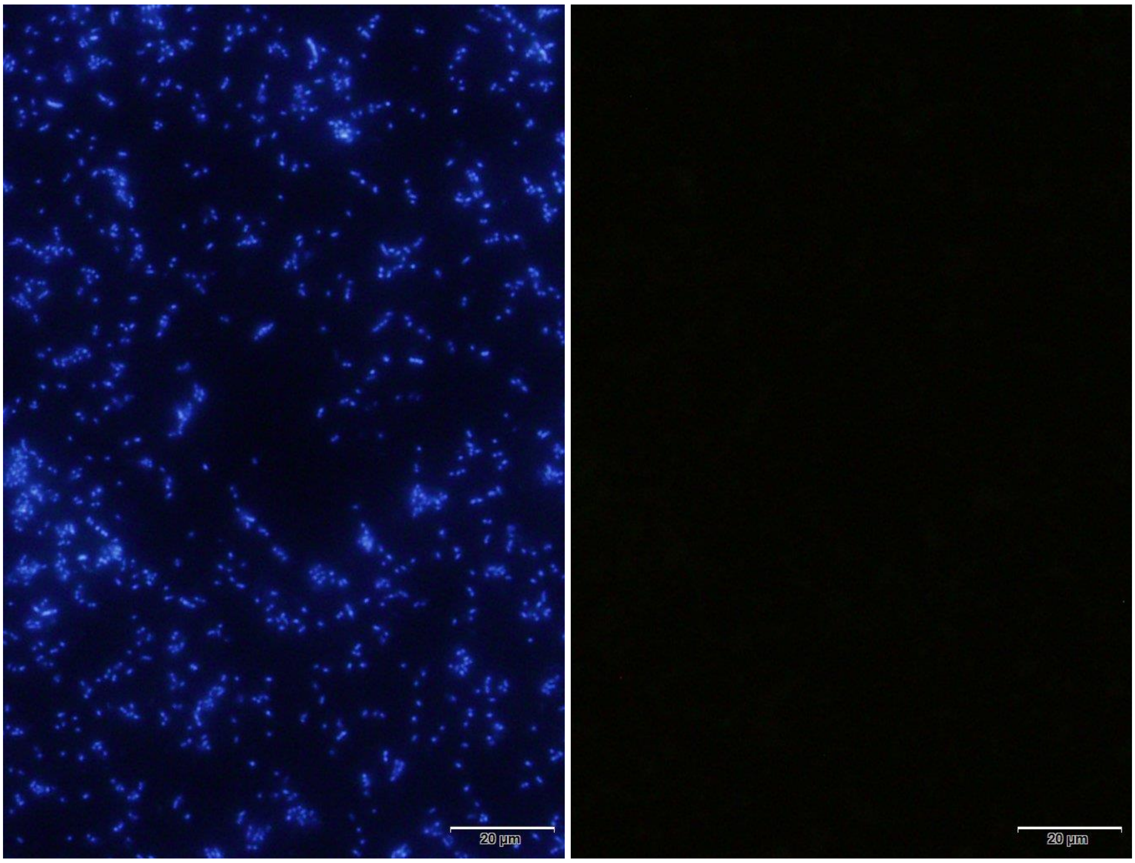

Prevotella copri **CCUG 58058T**

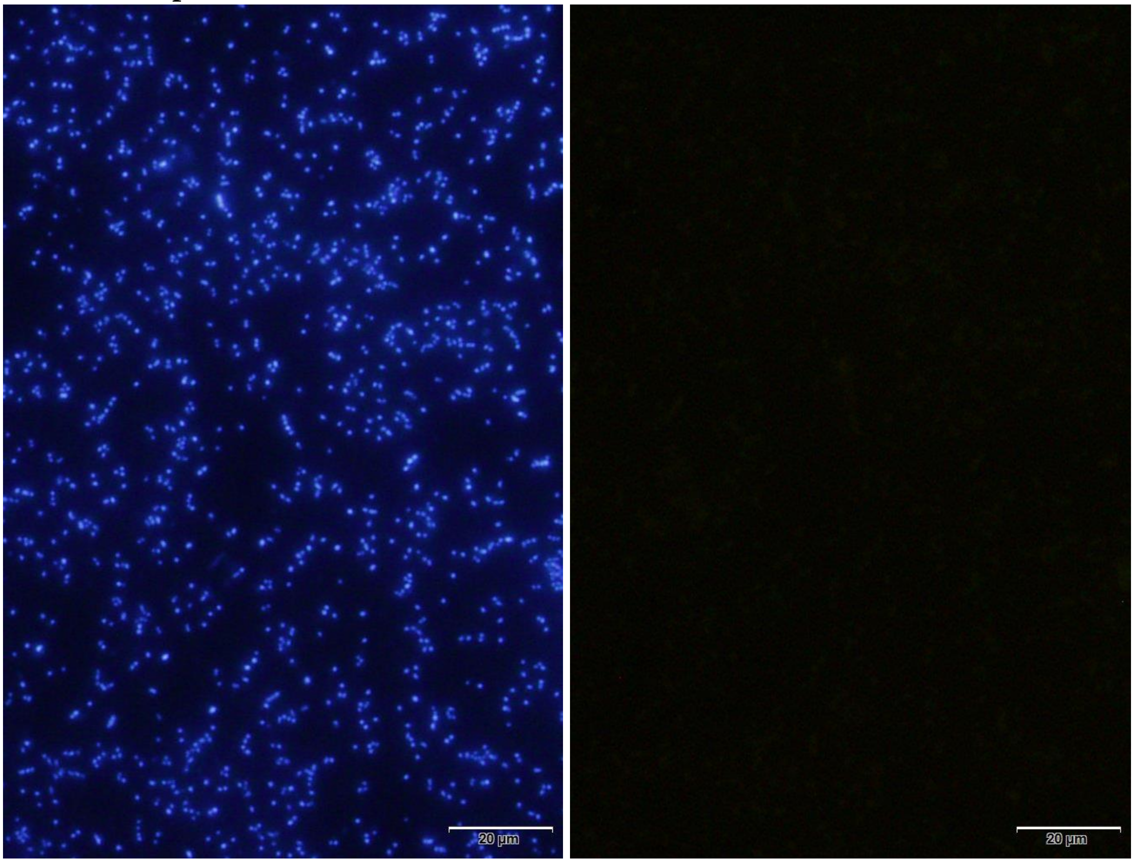

***Prevotella denticola* CCUG 29542T**

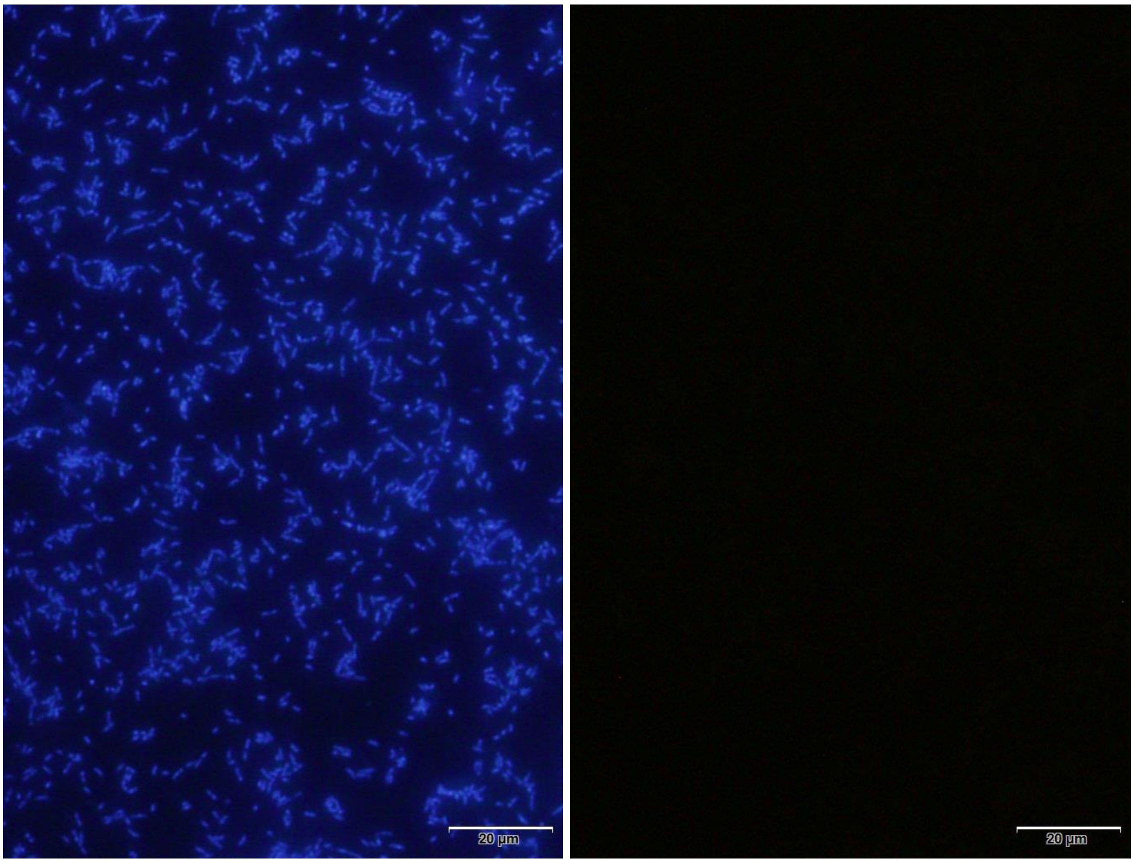

***Prevotella disiens* CCUG 59491**

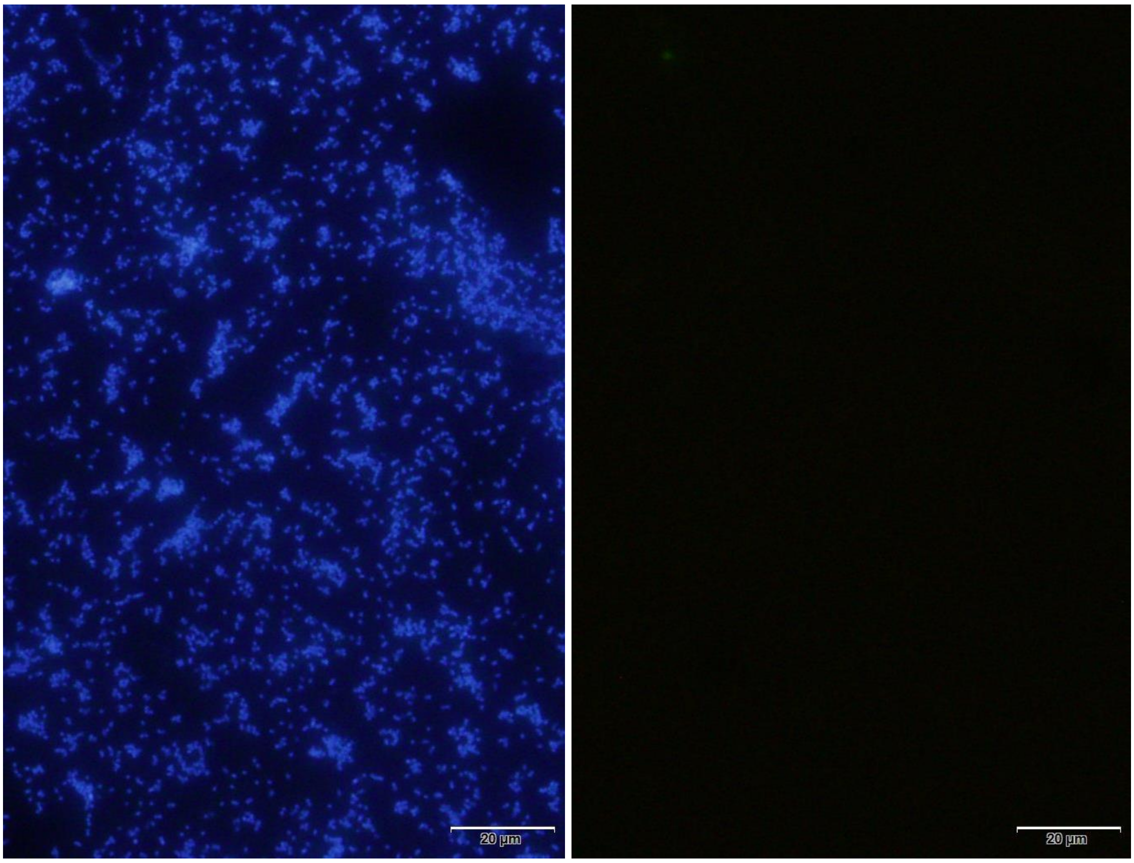

Prevotella intermedia CCUG 31410

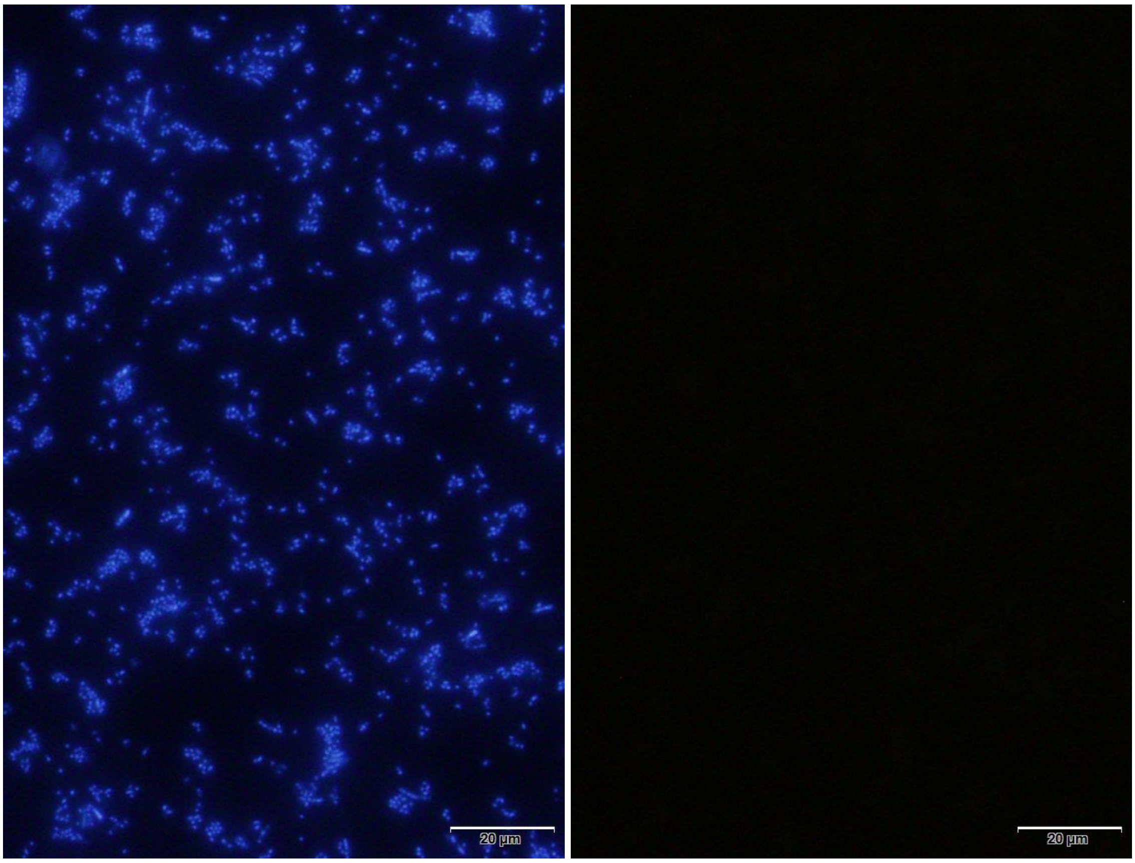

Prevotella melaninogenica CCUG 65141

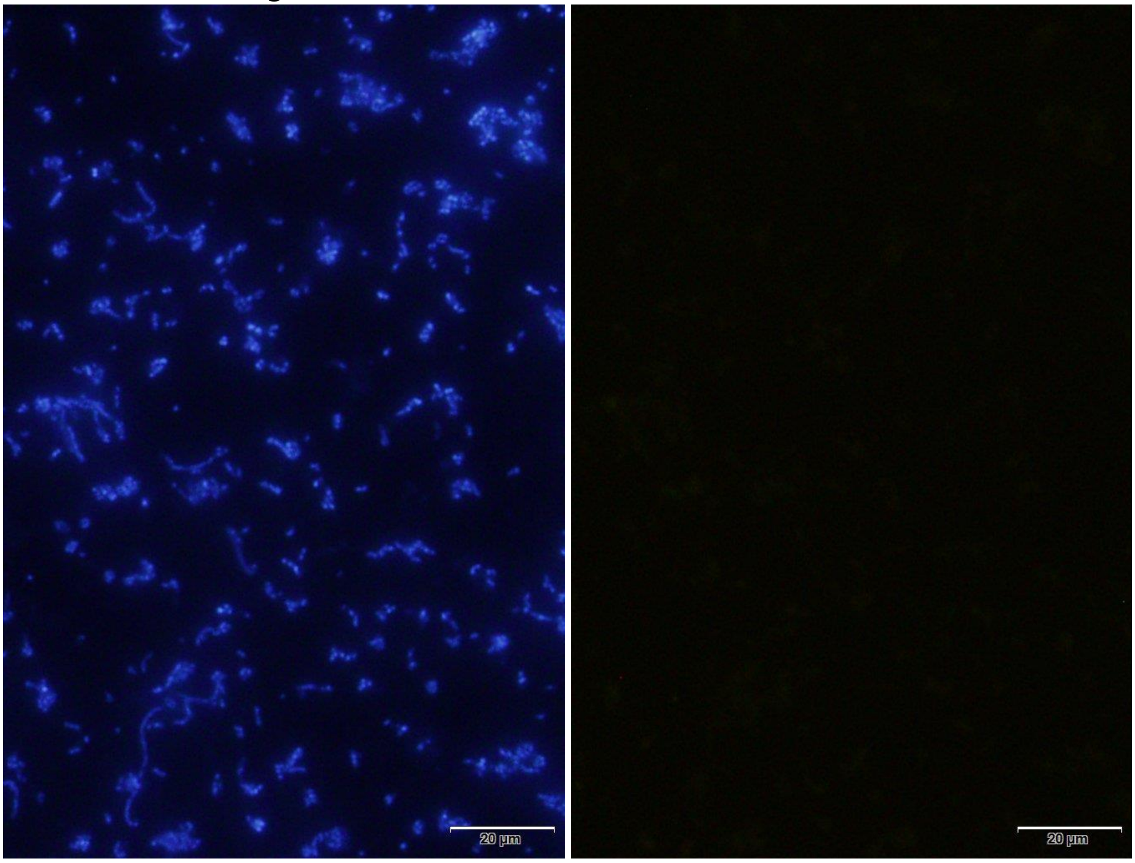

Prevotella nigrescens CCUG 25289

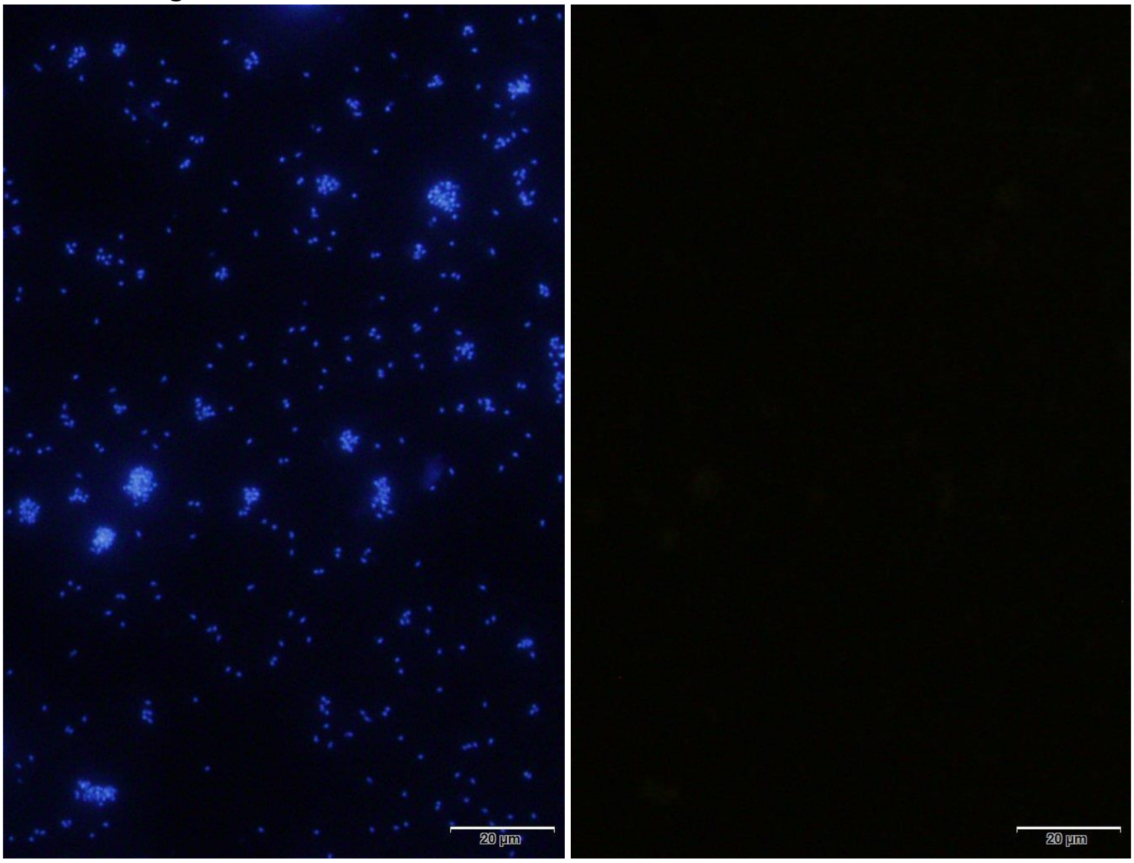

Prevotella timonensis CCUG 59487

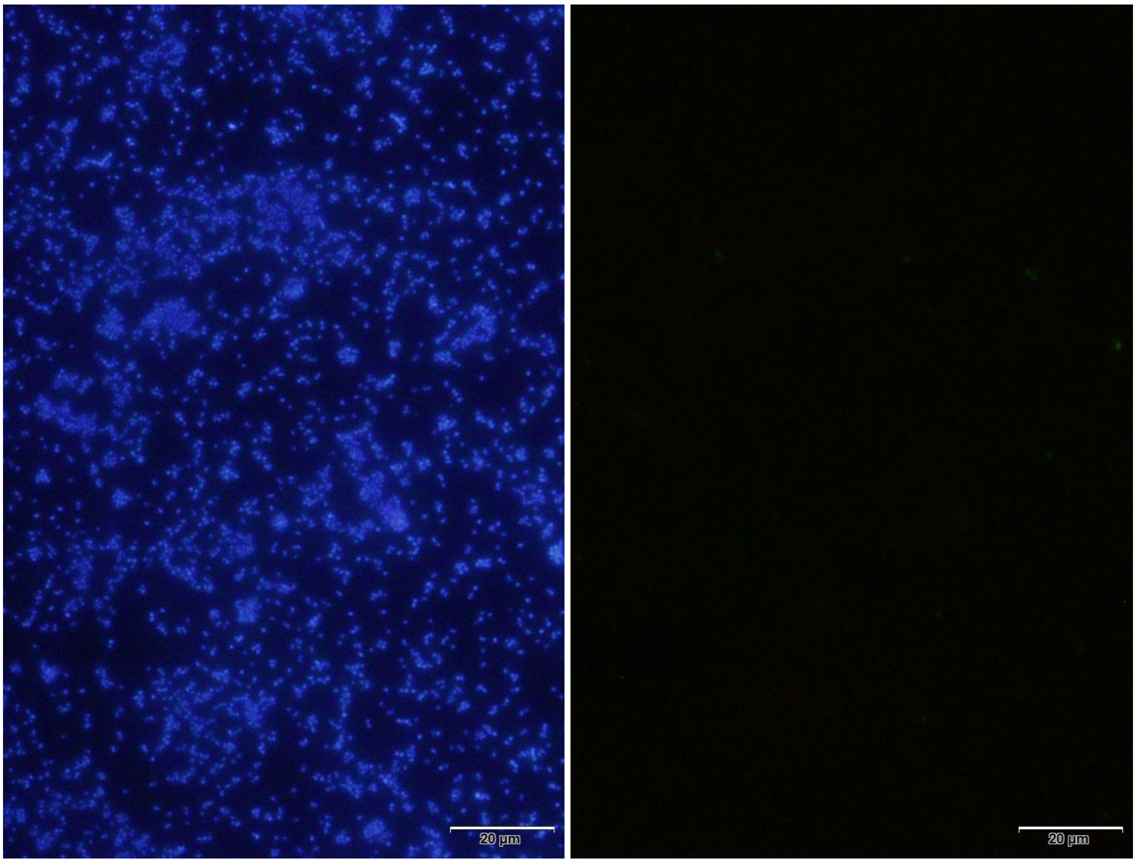

***Propionibacterium acnes* UM034**

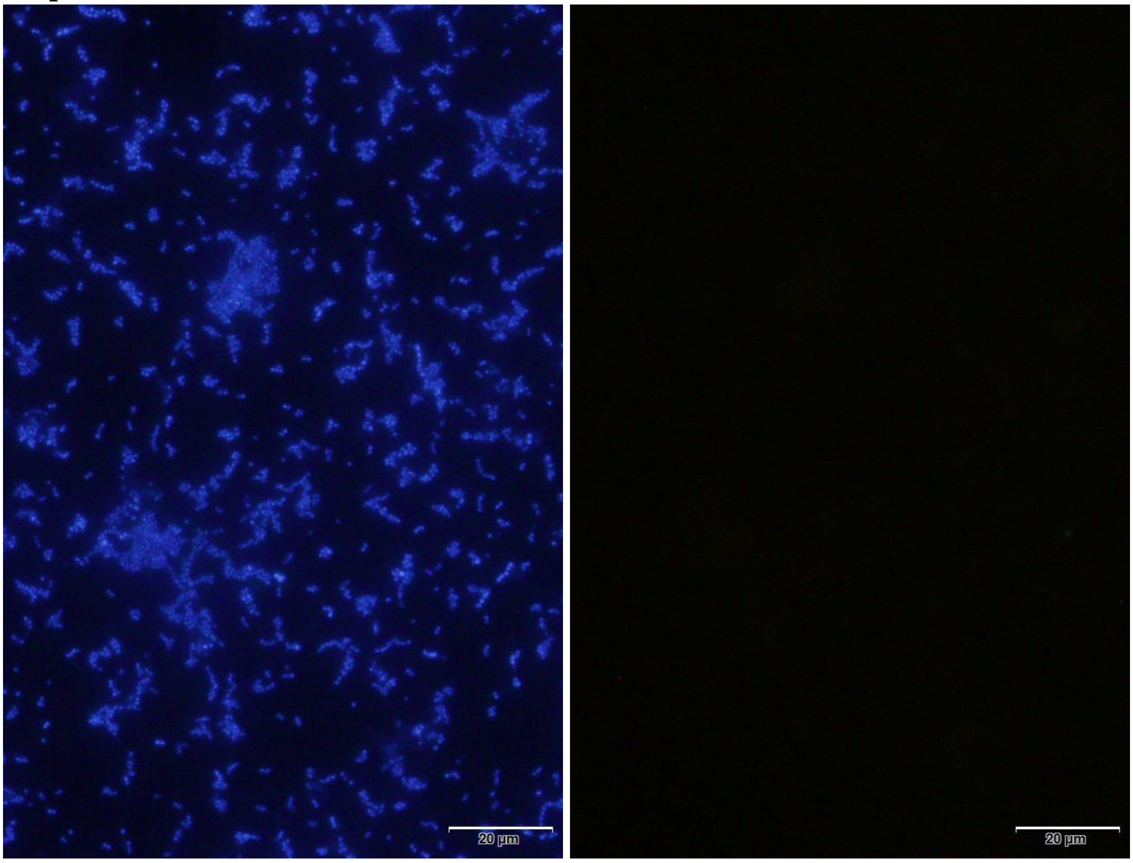

***Shigella* spp. UM137**

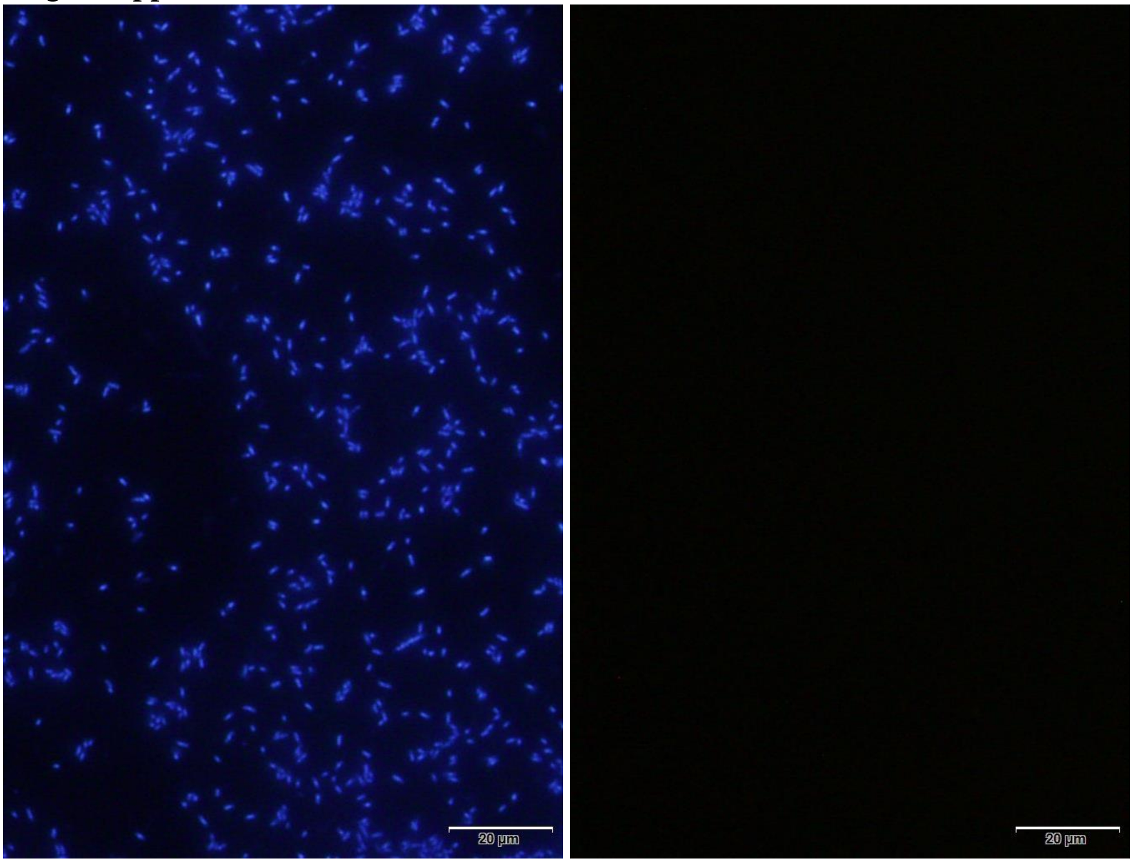

***Sneathia sanguinegens* CCUG 66076**

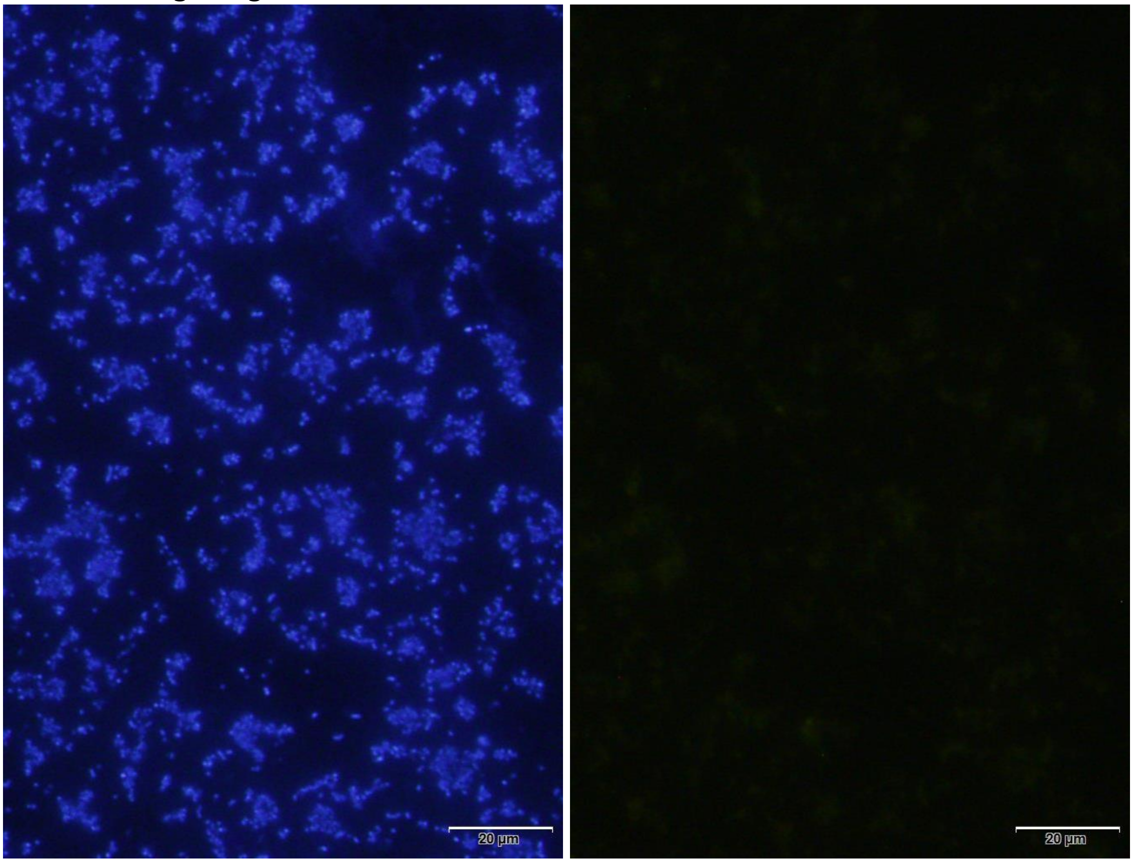

***Staphylococcus epidermidis* UM066**

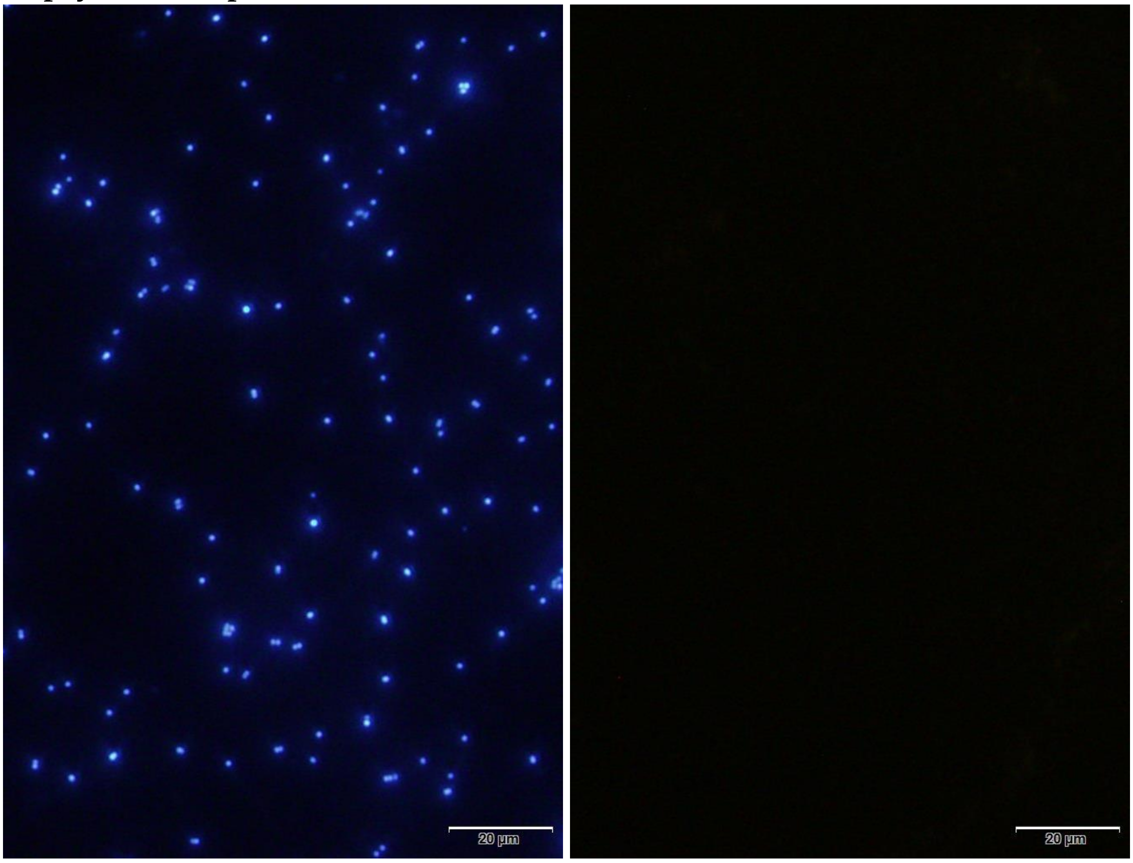

Staphylococcus haemolyticus **UM066**

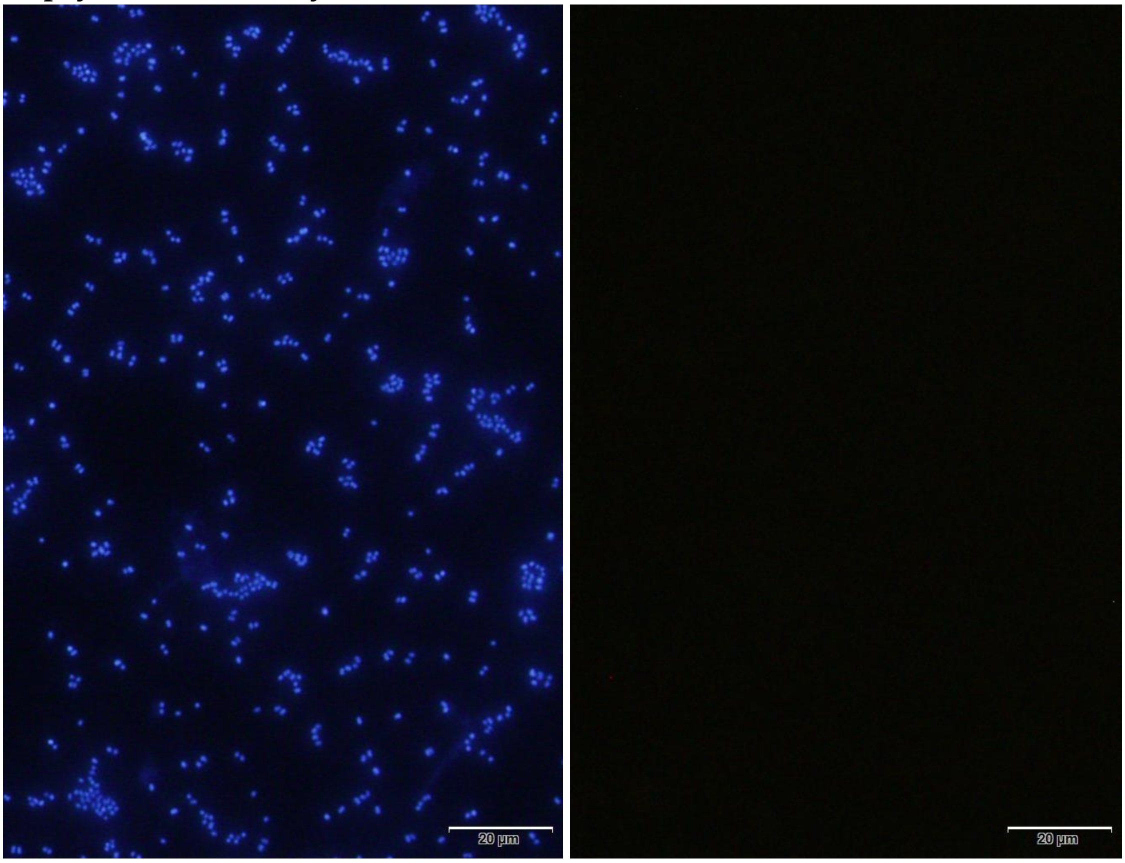

Staphylococcus hominis **UM224**

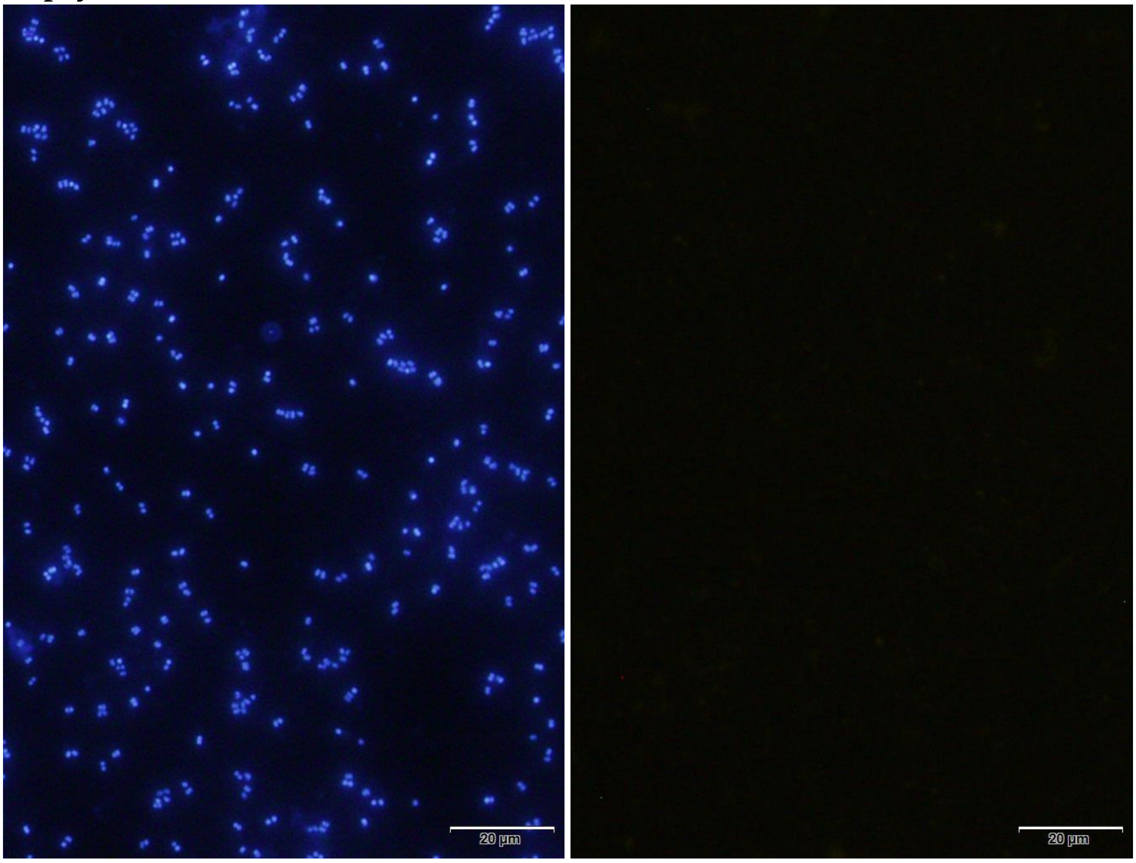

***Staphylococcus saprophyticus* UM121**

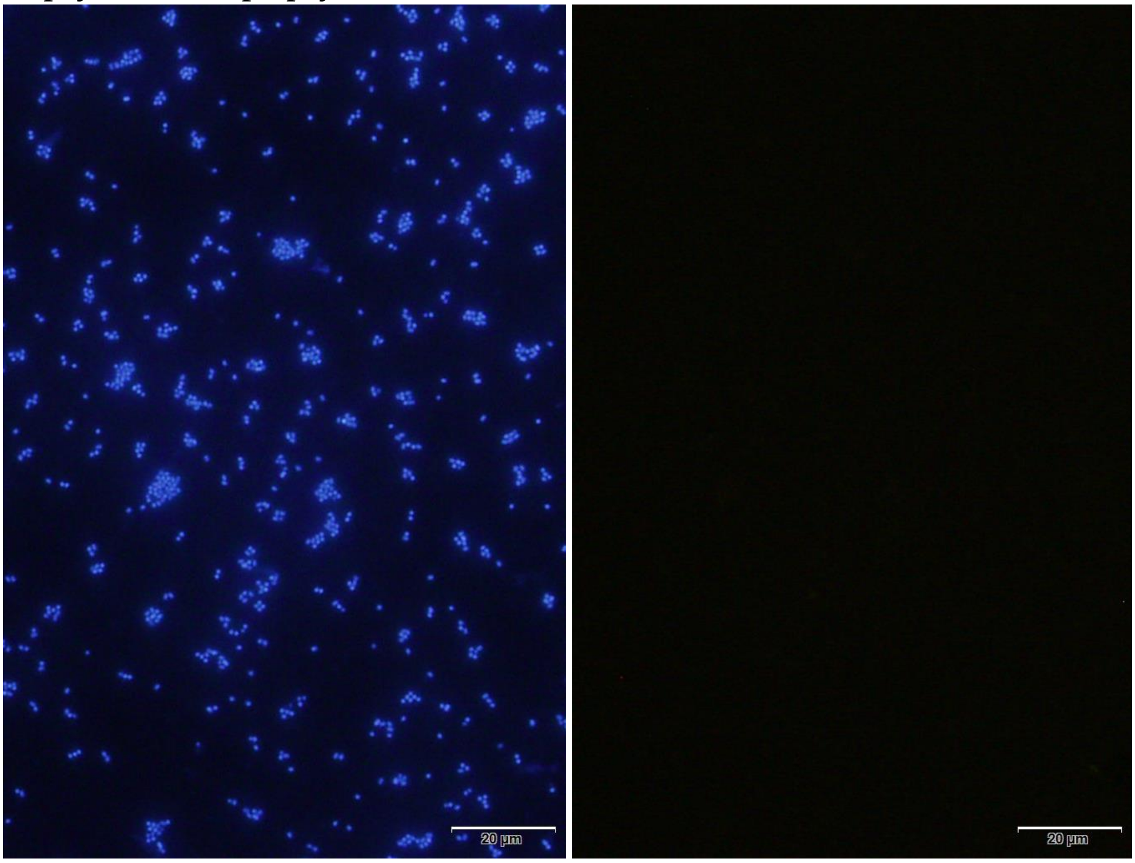

***Staphylococcus simulans* UM059**

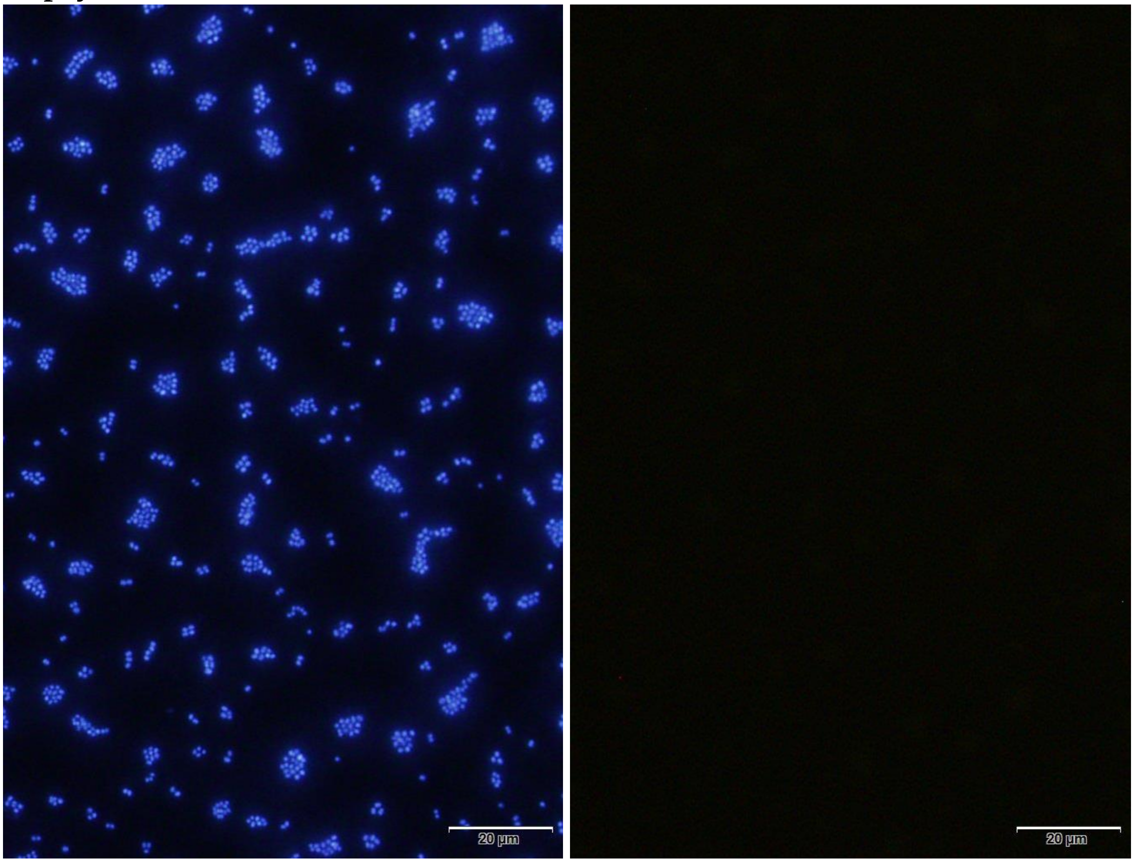

***Streptococcus agalactiae* UM035**

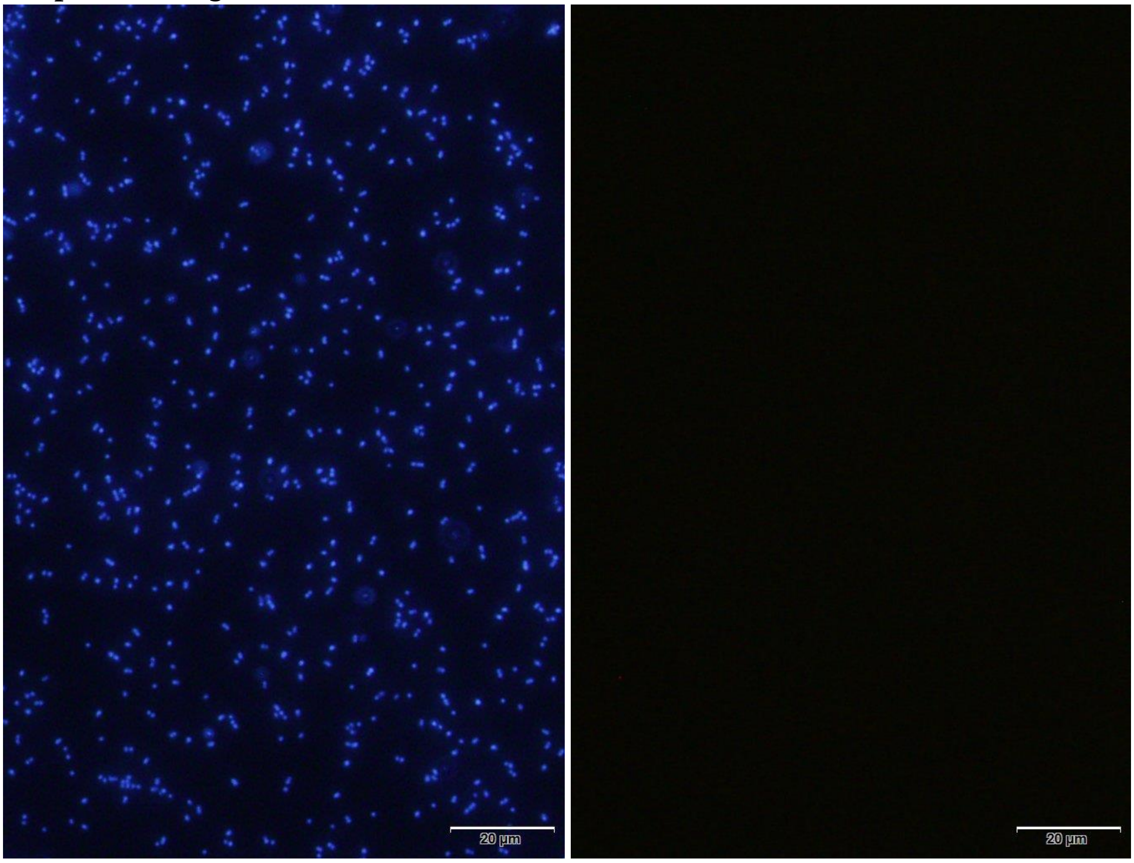

***Veillonella parvula* CCUG 59474**

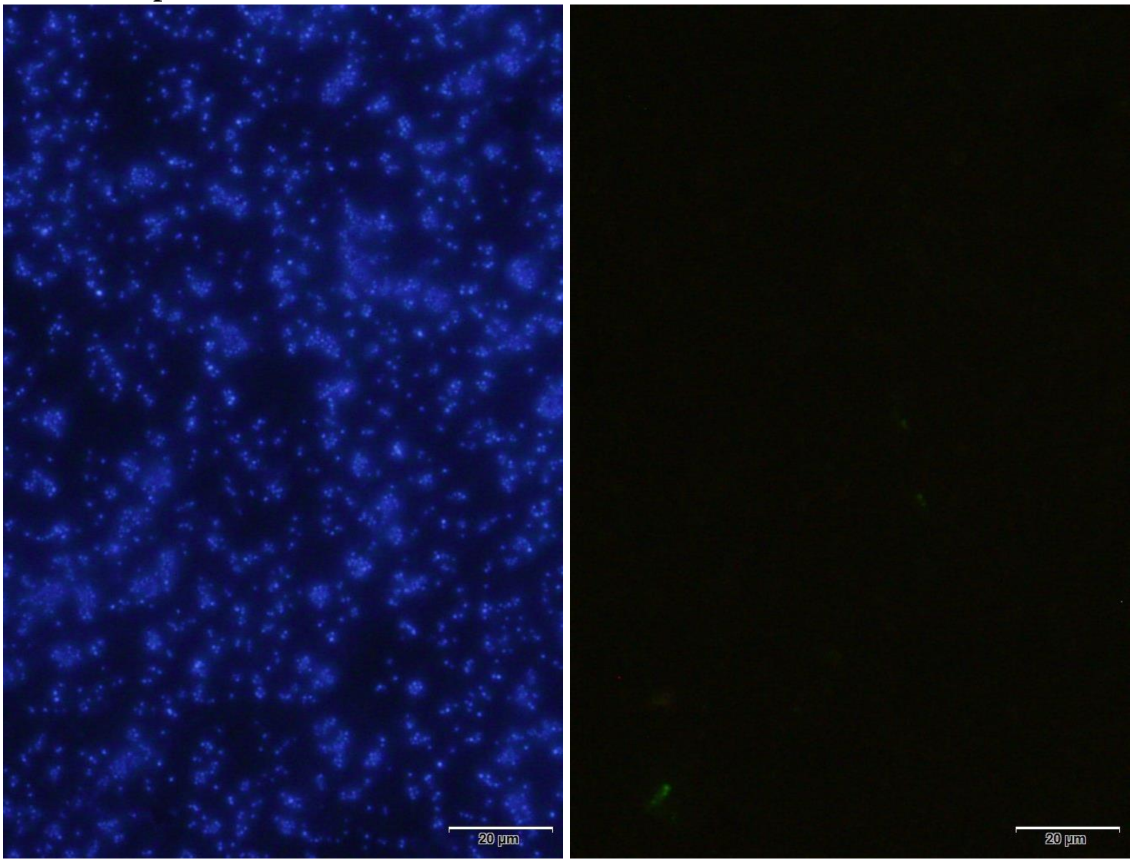

Supplementary Figure 4: The following images show the fluorescence microscopy results for some of the species that present autofluorescence. These species were observed without any probe added to the hybridization solution or without counterstaining (DAPI) to confirm if the autofluorescence signal is a result of non-specific hybridization of *P. bivia* PNA probe. The images were acquired on the DAPI filter (left image, blue) and FITC filter (right image, green), sensitive to the Alexa fluor 488, with a magnification of 400x; scale bars represent 20 µm.

Aerococcus christensenii CCUG 28826

Without PNA *P. bivia* probe

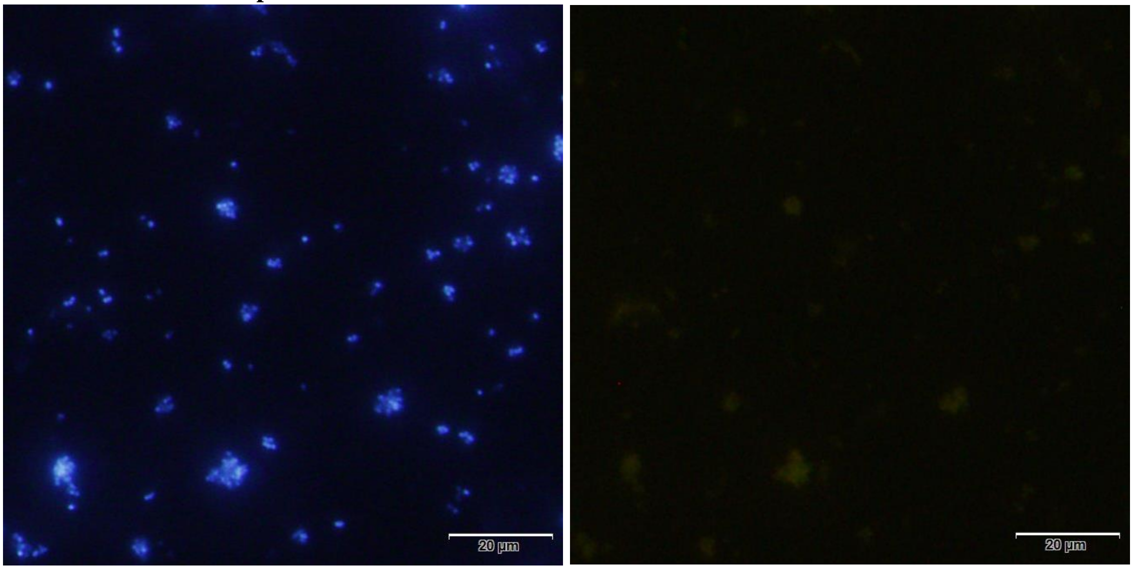

Without DAPI counterstaining

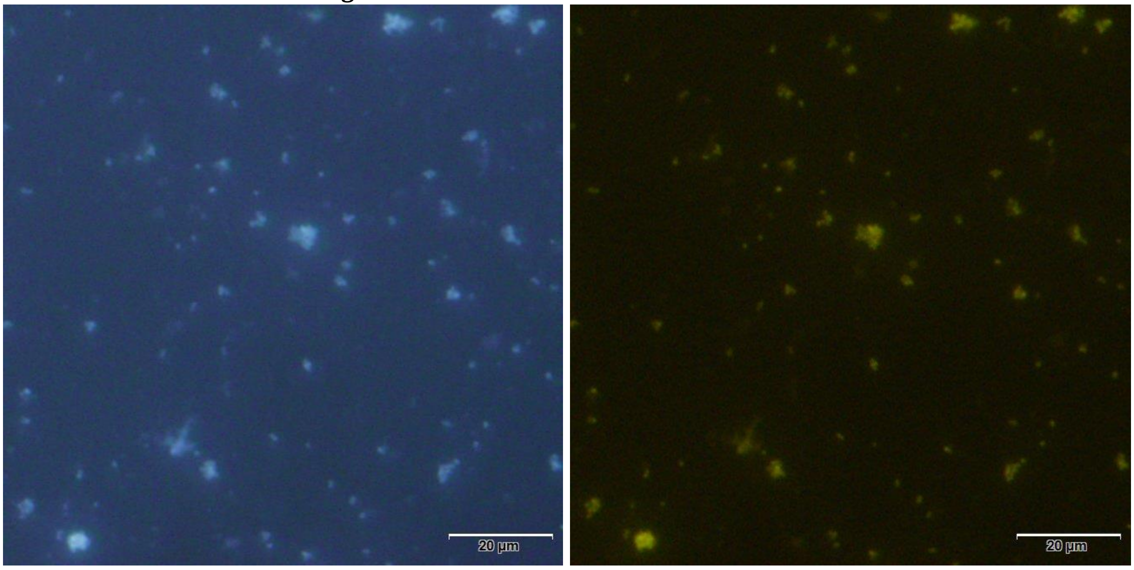

Enterococcus faecalis UM035

Without PNA *P. bivia* probe

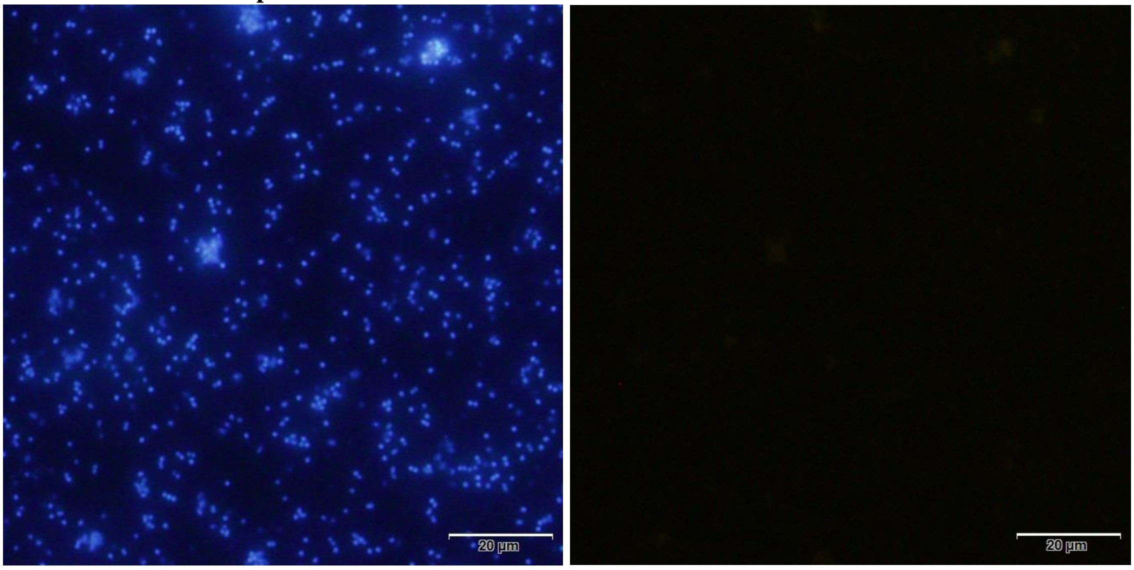

Without DAPI counterstaining

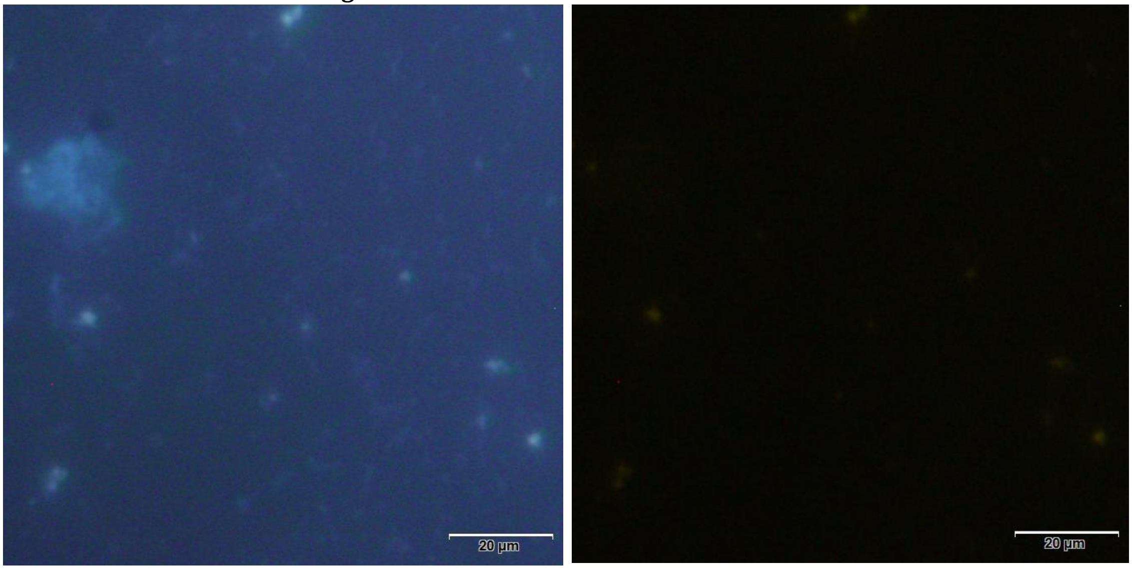

Gardnerella swidsinskii UM094

Without PNA *P. bivia* probe

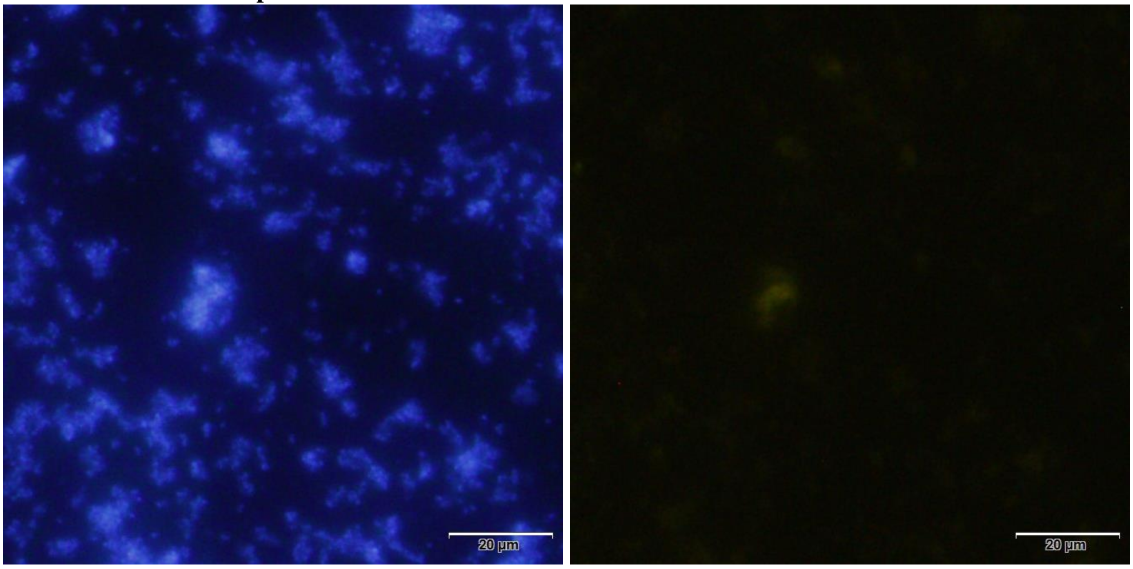

Without DAPI counterstaining

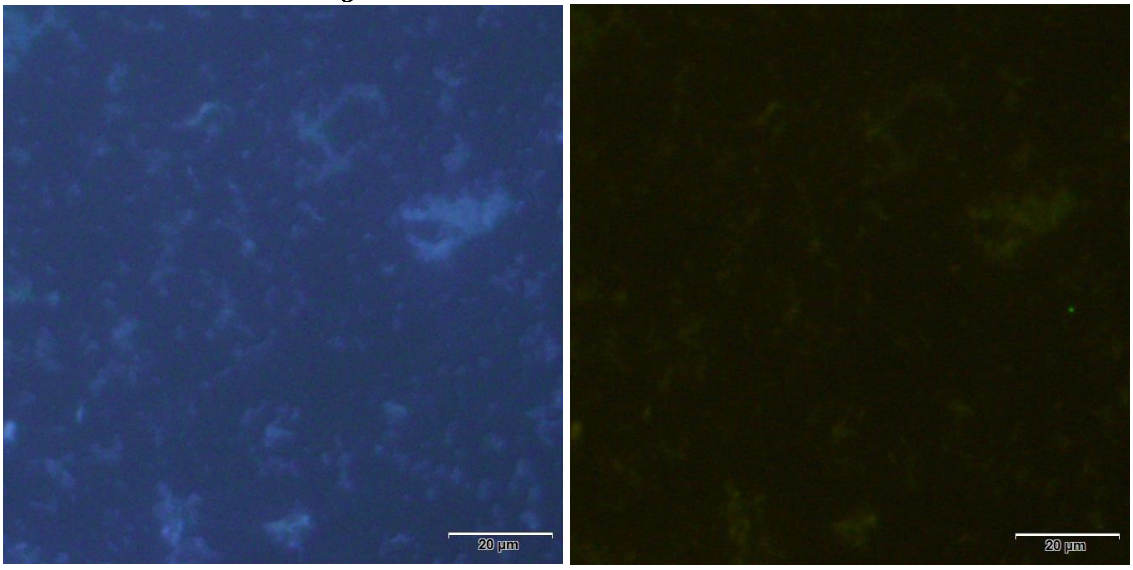

Gemella haemolysans UM034

Without PNA *P. bivia* probe

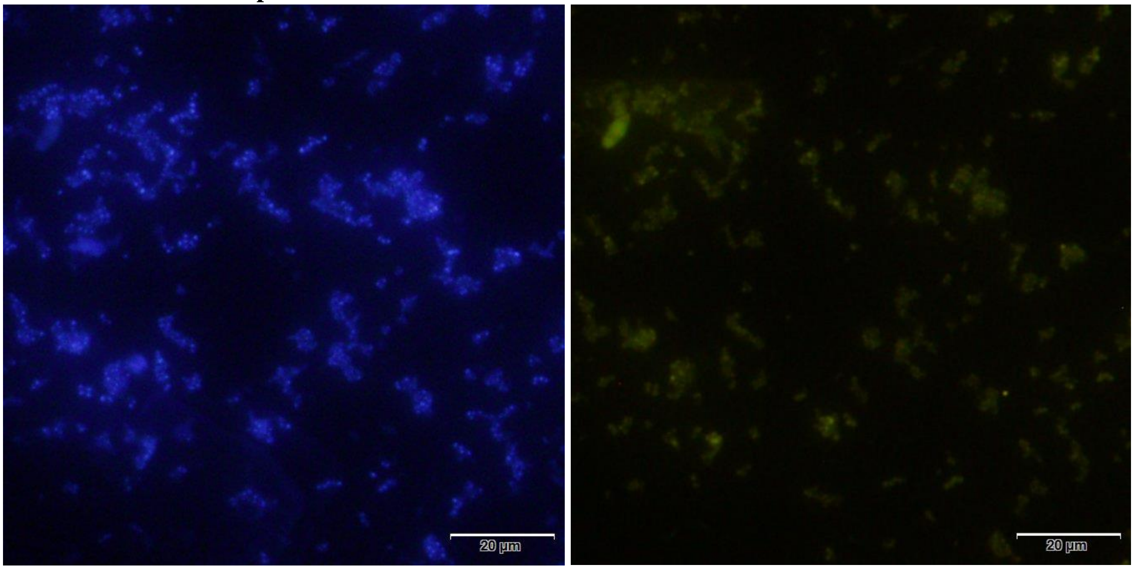

Without DAPI counterstaining

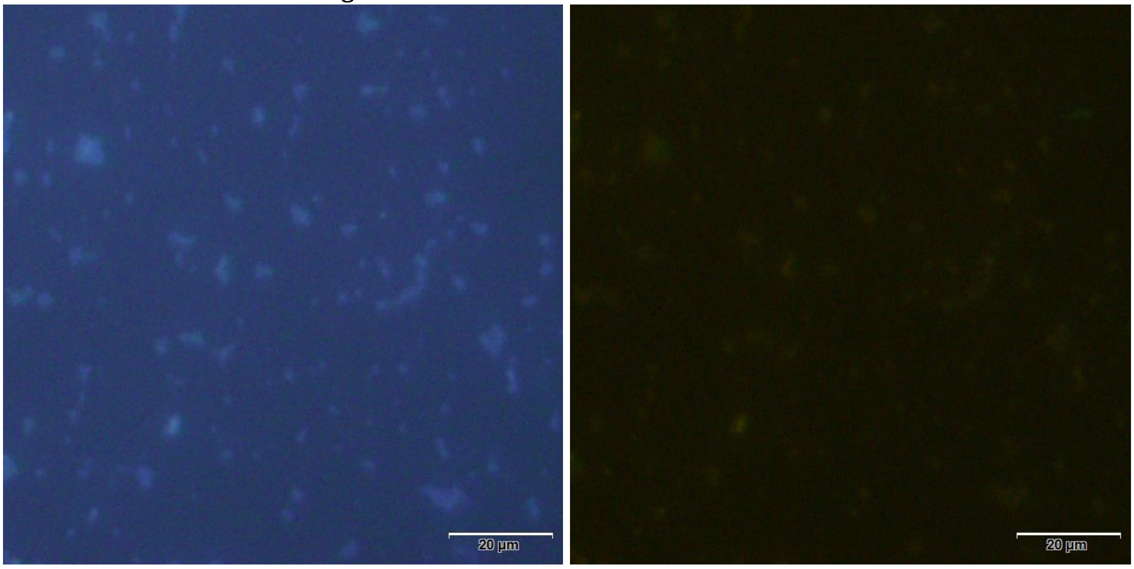

Sneathia sanguinegens CCUG 66076

Without PNA *P. bivia* probe

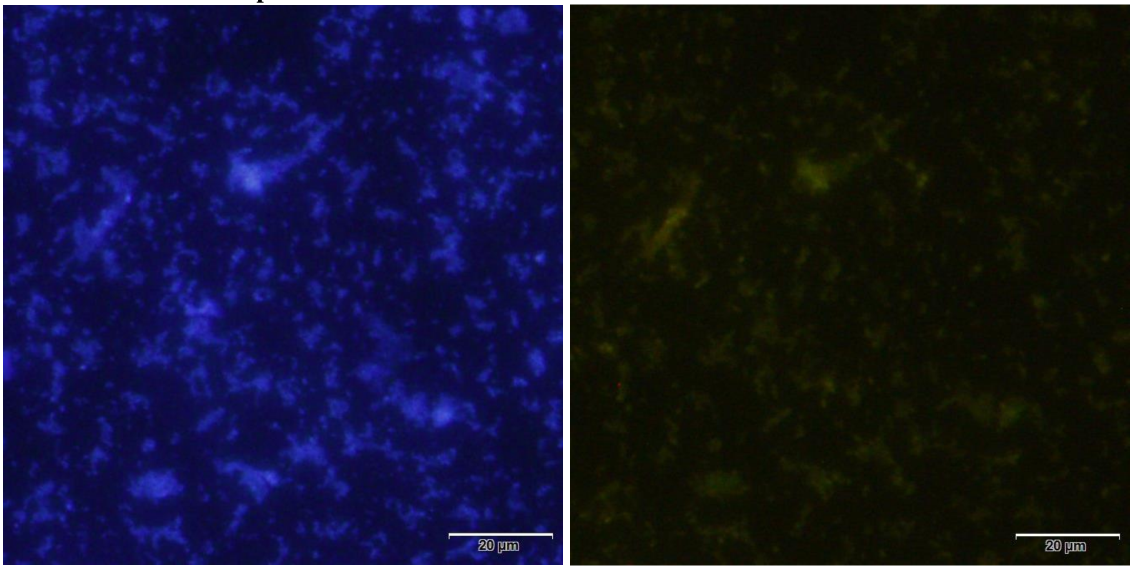

Without DAPI counterstaining

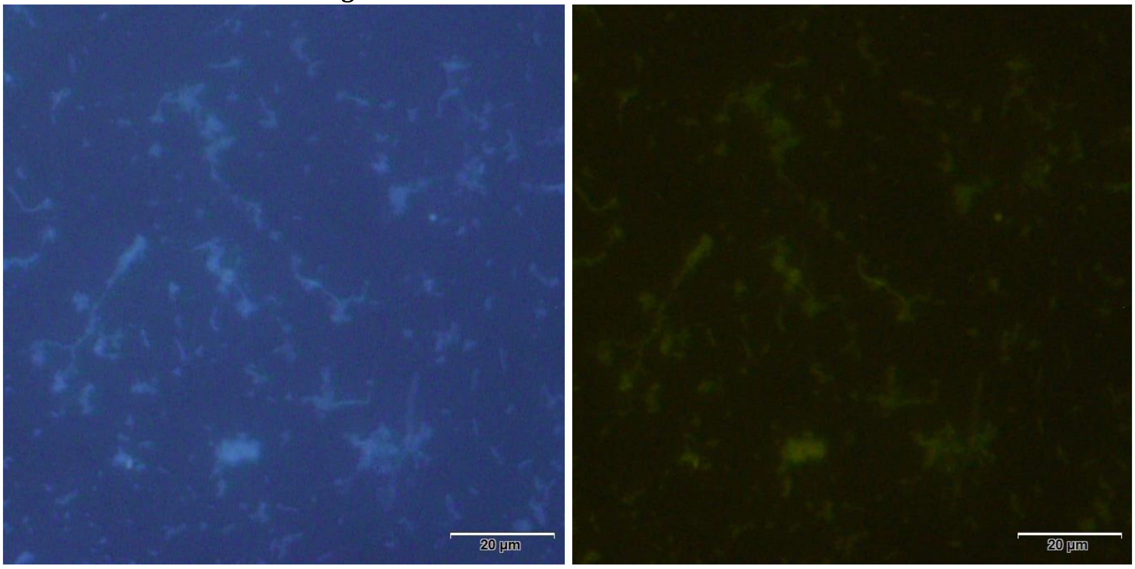

Supplement: Supplementary file 1 — Supplementary Information [file 41522_2023_411_MOESM1_ESM.pdf]
